# Polyploid QTL-seq revealed multiple QTLs controlling steamed tuber texture and starch gelatinization temperature in sweetpotato

**DOI:** 10.1270/jsbbs.23060

**Published:** 2024-02-29

**Authors:** Hiromoto Yamakawa, Tatsumi Mizubayashi, Masaru Tanaka

**Affiliations:** 1 Institute of Crop Science, National Agriculture and Food Research Organization (NARO), 2-1-2 Kannondai, Tsukuba, Ibaraki 305-8518, Japan; 2 Kyushu-Okinawa Agricultural Research Center, National Agriculture and Food Research Organization (NARO), 6651-2 Yokoichi-cho, Miyakonojo, Miyazaki 885-0091, Japan

**Keywords:** polyploid, QTL-seq, DNA marker, sweetpotato, flesh texture

## Abstract

Sweetpotato (*Ipomoea batatas*) includes diverse cultivars with flesh textures ranging from dry to moist. Moist-fleshed cultivars often contain starch with a lower gelatinization temperature (GT). To elucidate the genetic determinants of flesh texture and starch GT, we conducted a QTL analysis using F_1_ progenies obtained from a cross between dry-fleshed and moist-fleshed cultivars, ‘Benikomachi’ (BK) and ‘Amahazuki’ (AH), by using an updated polyploid QTL-seq pipeline. Flesh texture was assessed based on the wet area ratio (WAR) observed on the cut surface of steamed tubers, as progenies with dry and moist flesh exhibited low and high WAR values, respectively, demonstrating a strong correlation. Three QTLs were found to regulate the WAR. Notably, two AH-derived alleles at 4.30 Mb on Itr_chr05 and 21.01 Mb on Itr_chr07, along with a BK-derived allele at 2.89 Mb on Itr_chr15, were associated with increased WAR. Starch GT, which displayed no correlation with either flesh texture or WAR, was distinctly influenced by two QTLs: a GT-increasing BK-derived allele at 1.74 Mb on Itr_chr05 and a GT-decreasing AH-derived allele at 30.16 Mb on Itr_chr12. Consequently, we developed DNA markers linked to WAR, offering a promising avenue for the targeted breeding of sweetpotato with the desired flesh textures.

## Introduction

Sweetpotato (*Ipomoea batatas* L.) is a vital tuberous crop with a dominant production of approximately 85 million tons across Asian and African countries (FAOSTAT 2021). Beyond its role as a vegetable, sweetpotato finds application in the production of ‘shochu’ distilled liquor, starch industries, and processed food. In Japan, different cultivars showcasing varied textures of steamed tubers have been developed (Katayama *et al.* 2017). Cultivars such as ‘Beniazuma’ and ‘Benikomachi’ (BK) with dry textures are favored for traditional table use, whereas those with moist textures, such as ‘Beniharuka’ and ‘Amahazuki’ (AH), are gaining traction owing to their adhesive consistency and pronounced sweetness, which appeal to consumers.

Typically, sweetpotato tubers exhibit a dry texture and modest sweetness immediately after harvest. However, several weeks of storage induces a transition toward a moist texture and heightened sweetness, as evidenced by increased sucrose content (Nishinaka *et al.* 2020). This transformation from dry to moist texture, paralleled by augmented sweetness and Brix values, aligns with the conversion of storage starch into sugars. However, the pivotal determinants of the tuber texture remain unknown. AH has a unique attribute, displaying a moist texture and enhanced sweetness, even immediately after harvest (Nishinaka *et al.* 2022). This peculiarity makes the AH an ideal candidate for studying the underlying mechanisms dictating steamed tuber texture.

The conversion of storage starch into sugars is not only influenced by storage duration but also occurs during cooking processes involving heat, such as steaming. Sweetpotato tubers contain substantial quantities of β-amylase (Katayama *et al.* 2017). While the enzyme remains inert toward raw starch, it degrades starch to maltose after heat-induced gelatinization. This enzymatic reaction persists until enzyme denaturation occurs at elevated temperatures, contributing to increased sweetness during steaming. Varieties with lower tuber starch gelatinization temperatures (GT) initiate the conversion process earlier and prolong it, resulting in elevated maltose production and, consequently, sweeter sweetpotatoes. For instance, ‘Quick Sweet’, characterized by a GT 20°C lower than that of ‘Beniazuma’ (Katayama *et al.* 2002, 2004), has increased maltose after steaming (Nakamura *et al.* 2014). Thus, both starch GT and the extent and thermostability of β-amylase activity are pivotal for starch-to-sugar conversion and potentially affect steamed tuber texture. Notably, the moist cultivar ‘Beniharuka’ consists of starch with a lower GT and elevated β-amylase activity compared to the dry cultivar ‘Beniazuma’ (Nakamura *et al.* 2018, Nishinaka *et al.* 2020). The reduced GT of ‘Quick Sweet’ results from a recessive locus (Katayama *et al.* 2015), and transgenic tubers with suppressed expression of the *starch synthase II* (*SSII*) gene exhibit decreased GT, similar to that of ‘Quick Sweet’ tubers (Kitahara *et al.* 2011, Takahata *et al.* 2010). Therefore, GT appeared to be modulated by starch synthase II. Nonetheless, the genetic factors orchestrating steamed tuber texture remain unclear.

Sweetpotato possesses a hexaploid genome and intricate inheritance patterns, posing challenges in deciphering the genetic mechanisms underlying tuber quality traits such as steamed tuber texture. Recently, a whole-genome sequencing-based quantitative trait locus (QTL) analysis method called polyploid QTL-seq was developed (Yamakawa *et al.* 2021). This technique facilitates genetic mapping using unique simplex single nucleotide polymorphisms (SNPs) specific to each homoeologous chromosome, enabling the identification of genomic regions linked to purple flesh. Using this approach, we aimed to unravel the genetic basis of tuber texture by investigating the F_1_ progenies derived from a cross between the moist-fleshed AH and dry-fleshed BK cultivars. In this study, we identified three distinct QTLs governing steamed tuber texture, separate from those influencing starch GT, and present DNA markers for the genetic control of steamed tuber texture.

## Materials and Methods

### Plant materials

The F_1_ progenies, resulting from crosses between *I. batatas* cv. ‘Amahazuki’ (AH; moist flesh texture) and cv. ‘Benikomachi’ (BK; dry flesh texture), were cultivated in the field. Genomic DNA was extracted from the unexpanded leaves of 126 progenies and their parents. Tuberous roots from the same individuals were collected and stored at around 15°C for approximately one week before starch preparation and about six weeks before the assessment of steamed tubers. Tubers for amylase activity determination were sampled after three months of storage and stored at –80°C.

### Evaluation of steamed tuber texture

Tubers were washed and steamed for 2 h using a GK-14 steamer (Goka, Tokyo, Japan). Transverse cuts were made on the steamed tubers, and the section surfaces were photographed and visually inspected to determine the wet area ratio (WAR), which represents the ratio of the wet area to the total area. The texture was graded based on the following criteria: 1 (dry), 2 (moderately dry), 3 (intermediate), 4 (moderately wet), and 5 (wet).

### Preparation of tuber starch and measurement of gelatinization temperature

Approximately 50 g of sweetpotato tubers were used for starch preparation. The tubers were then washed and diced into small pieces. The diced samples (50 g) were homogenized in 200 mL of distilled water in a mixer (SM-KM39; SANYO, Osaka, Japan). The resulting slurry was filtered through a 150 μm metallic sieve to allow most starch granules to pass. The starch suspension was allowed to stand for 2 h, after which the starch granules were recovered by decantation. The remaining starch pellets were washed thrice with distilled water and dried under vacuum. Purified starch samples were stored at 4°C for subsequent analysis.

The gelatinization temperature (GT) of starch was measured using a differential scanning calorimeter (DSC-60; Shimadzu, Kyoto, Japan). Purified starch (5–10 mg) was mixed with distilled water (4 volumes), and the mixture (20 μL) was subjected to heating from 35 to 100°C at a rate of 5°C per minute. The peak temperature (*T*p) was considered to be the GT.

### Genomic DNA extraction, bulking, and sequencing

Genomic DNA was extracted from the unexpanded leaves of the shoot apex using ISOSPIN Plant DNA (Nippon Gene, Tokyo, Japan). Bulked DNA samples were prepared by combining equal amounts of DNA from the F_1_ individuals. Sequencing libraries were constructed using a TruSeq DNA PCR-Free Sample Preparation Kit (Illumina, San Diego, CA, USA) and subjected to 150-bp paired-end sequencing using an Illumina NovaSeq6000 DNA sequencer. FASTQ sequence data are available from the DDBJ Sequence Read Archive under the accession number DRA016029.

### Polyploid QTL-seq analysis

Polyploid QTL-seq analysis was conducted using the polyploid QTL-seq pipeline ver 1.0.0, which is available on GitHub (https://github.com/TatsumiMizubayashi/PolyploidQtlSeq). The analysis was performed on an iMac Pro computer equipped with a 36-thread Intel Xeon W processor, 128 GB RAM, and 4 TB HDD. The pipeline utilizes the following programs: .NET6 version 6.0.400, fastp version 0.23.2, bwa version 0.7.17-r1188, samtools version 1.15.1, bcftools version 1.15.1, bgzip, tabix, and snpEff version 5.0. Quality control of reads for both parents and moist and dry bulks followed the default settings, whereas the QTL-seq analysis with simplex variants used modified configuration parameters as follows: minMQ = 30, adjustMQ = 70, p2SnpIndexRange = 0.08–0.25, minDepth = 50, and ploidy = 6. In the case of the analyses with duplex and triplex variants, p2SnpIndexRange parameter was changed to 0.22–0.45 and 0.38–0.62, while NPlex parameter was set to 2 and 3, respectively. The reference genome used was the chromosome-scale genome sequence of the highly homozygous accession Mx23Hm of the diploid *I. trifida* ITR_r2.2 (Hirakawa *et al.* 2015, https://doi.org/10.1101/2022.12.25.521700). The analysis was repeated with the P1 and P2 parents swapped to detect QTLs derived from both parents. Counts of variants employed in each QTL analysis were listed in Supplemental Table 1. QTL candidate regions were identified when the region met both of the following criteria: 1) the QTL variant count, that is the number of QTL-deduced variants in the 100-kb sliding window, was more than 20 over a 2 Mb region in one direction of QTL effect but not in the other direction, and 2) SNPs whose SNP-index were 0 (indicated by dark green or orange dots in the SNP-index graphs) were clustered over a 2 Mb region in one bulk but not in the other bulk. A detailed protocol is available in the user manual on GitHub (https://github.com/TatsumiMizubayashi/PolyploidQtlSeq).

### Preparation of linked DNA markers and genotyping of F_1_ individuals

PCR primers were designed for SNP sites in the middle of the QTL-seq-identified candidate regions (Supplemental Table 6) to evaluate the effects of the QTLs on the traits. PCR mixtures (total 10 μL) containing 1× GoTaq Colorless Master Mix, 10 ng of genomic DNA from each F_1_ individual, and 2 pmol each of forward and reverse primers (or those for *SSII* as a control; Supplemental Table 6) were used for PCR. The cycling parameters were: initial denaturation at 95°C for 2 min; 30 cycles of denaturation at 95°C for 30 s, annealing at 55°C for 30 s, and extension at 72°C for 30 s (1 min for *SSII*); and a final extension at 72°C for 2 min. The amplicons were verified by electrophoresis on a 1.5% agarose gel.

### Determination of starch-lytic amylase activities

Quantitative determination of α-amylase activity was conducted using a maltopentaose-based colorimetric substrate kit (Kikkoman Biochemifa Company, Tokyo, Japan) following the manufacturer’s protocol. Flesh sections (2 g) from the central region of sweetpotato tubers were homogenized in extraction buffer (10 mM acetate-Na, 0.5% NaCl, pH 5.0) using a Polytron homogenizer (PT-10; Kinematica, Malters, Switzerland), and the suspension was incubated at 24°C for 1 h with occasional vertical mixing. After centrifugation at 20,000 × *g* for 5 min at 4°C, the supernatant was used for α-amylase activity determination. The reaction was initiated by mixing 10 μL of the 20-fold diluted extract with 50 μL of the substrate solution and 50 μL of the enzyme solution, conducted by incubation at 37°C for 10 min, and then terminated by adding 200 μL of the stop solution. The activity was determined by measuring the absorbance at 400 nm using a Multiskan GO microplate reader (Thermo Fisher Scientific, Waltham, MA, USA). One unit of α-amylase activity was defined as the amount of enzyme required to release 1 μmol 2-chloro-4-nitrophenol (CNP) from N3-G5-β-CNP per min.

Activity of β-amylase was quantitatively determined using a maltotriose-based colorimetric substrate kit (Megazyme, Wicklow, Ireland) according to the manufacturer’s protocol. Briefly, flesh sections (1 g) from the central region of sweetpotato tubers were homogenized in 10 mL of extraction buffer (50 mM Tris-HCl, 100 mM cysteine, 1 mM EDTA, pH 8.0) using a Polytron homogenizer, and the suspension was incubated at 24°C for 1 h with occasional vertical mixing. Following centrifugation at 20,000 × *g* for 5 min at 4°C, the supernatant was diluted 20 times with dilution buffer (100 mM MES, 1 mM EDTA, 1 mg/ml BSA, pH 6.2) and used to determine β-amylase activity. The reaction was started by mixing 20 μL of the diluted extract with 20 μL of the substrate solution, conducted by incubation at 40°C for 10 min, and then terminated by adding 300 μL of 1% Tris-HCl, pH 8.5. The activity was determined by measuring the absorbance at 400 nm using a Multiskan GO microplate reader. One unit of β-amylase activity was defined as the amount of enzyme required to release 1 μmol *p*-nitrophenol (PNP) from PNPβ-G3 per min. To verify thermostability, an aliquot of diluted β-amylase was incubated at the indicated temperature for 10 min prior to the assay.

### Accession numbers

FASTQ sequence data are available from the DDBJ Sequence Read Archive under the accession number DRA016029.

## Results

### Flesh texture of AH, BK, and their F_1_ progenies

**Fig. 1.**
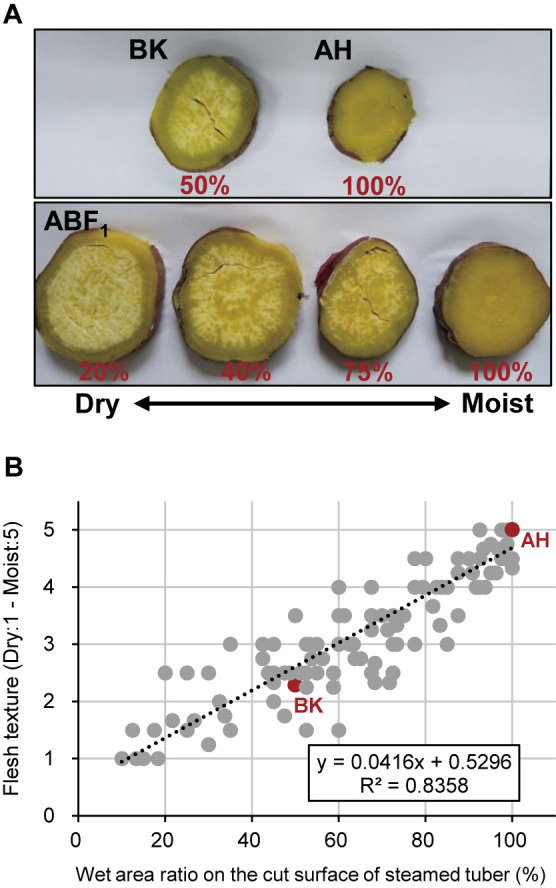
Appearance of steamed tuber flesh surfaces and correlation of wet area ratio (WAR) to flesh texture. (A) Surface appearance of steamed tuber sections for ‘Benikomachi’ (BK), ‘Amahazuki’ (AH), and F_1_ individuals (ABF_1_). Corresponding WAR values determined visually are provided beneath each sample. (B) Correlation between WAR on steamed tuber surfaces and flesh texture grades, ranging from dry (1) to moist (5).

Steamed tubers from the parental cultivars BK and AH exhibited moderately dry and moist textures, respectively. Conventionally, tuber flesh texture is assessed by tasting and is scored on a scale from 1 (dry) to 5 (wet). However, this approach requires trained evaluators for an accurate grading. As an alternative, the wet area ratio (WAR), defined as the ratio of the wet surface area to the total area of the transverse section of the steamed tubers, was determined via visual inspection. Among BK, AH, and their F_1_ progenies (ABF_1_), WAR exhibited a robust correlation (*R*^2^ = 0.84) with flesh texture, as assessed by tasting (Fig. 1). Therefore, in subsequent analyses, flesh texture was quantified using WAR.

**Fig. 2.**
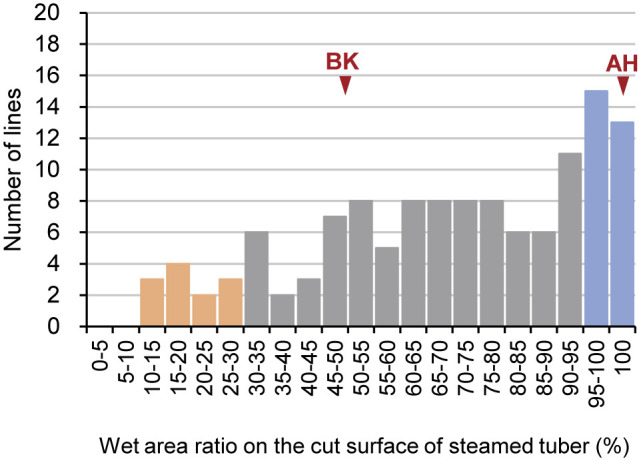
Wet area ratio (WAR) on the cut surface of steamed tubers for BK, AH, and ABF_1_ progenies. WAR averages for tests conducted in 2021 and 2022 are presented. The number of ABF_1_ lines with specific WAR values is indicated. Red arrowheads highlight WAR values of 50% for BK and 100% for AH. Criteria for defining dry and moist bulks for QTL-seq analysis are denoted in orange and blue, respectively.

An evaluation spanning two years (2021 and 2022) revealed that WAR presented a wide continuum, ranging from 12.5% to 100%, averaging 68.7%, among the 126 ABF_1_ individual lines. In contrast, BK and AH exhibited WAR values of 50% and 100%, respectively (Fig. 2). A noteworthy observation was that a substantial proportion (31%) of the ABF_1_ lines exhibited moist textures (WAR >90%), similar to AH. Intriguingly, 24% of the progenies displayed textures that were drier than those of BK (WAR below 50%), indicating that multiple genetic loci govern flesh texture.

### Starch GT of AH, BK, and their F_1_ progenies

**Fig. 3.**
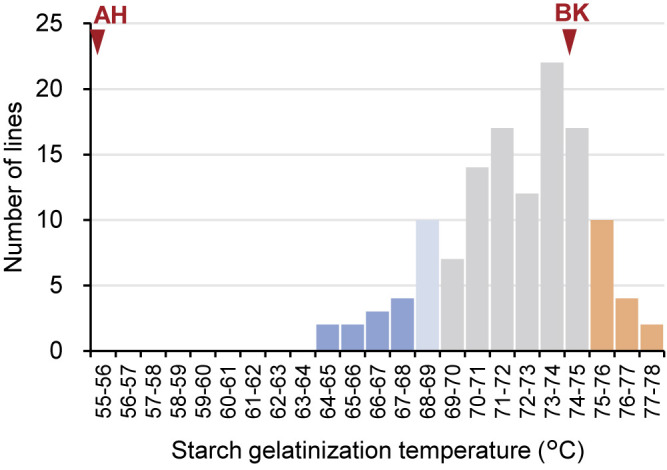
Gelatinization temperature (GT) of tuber starch for BK, AH, and ABF_1_ progenies. The number of ABF_1_ lines with specific temperatures is shown. Red arrowheads indicate temperatures of 74.1°C for BK and 55.4°C for AH. Criteria for low- and high-temperature bulks for QTL-seq analysis are denoted in blue (with light blue signifying partial line utilization) and orange, respectively.

**Fig. 4.**
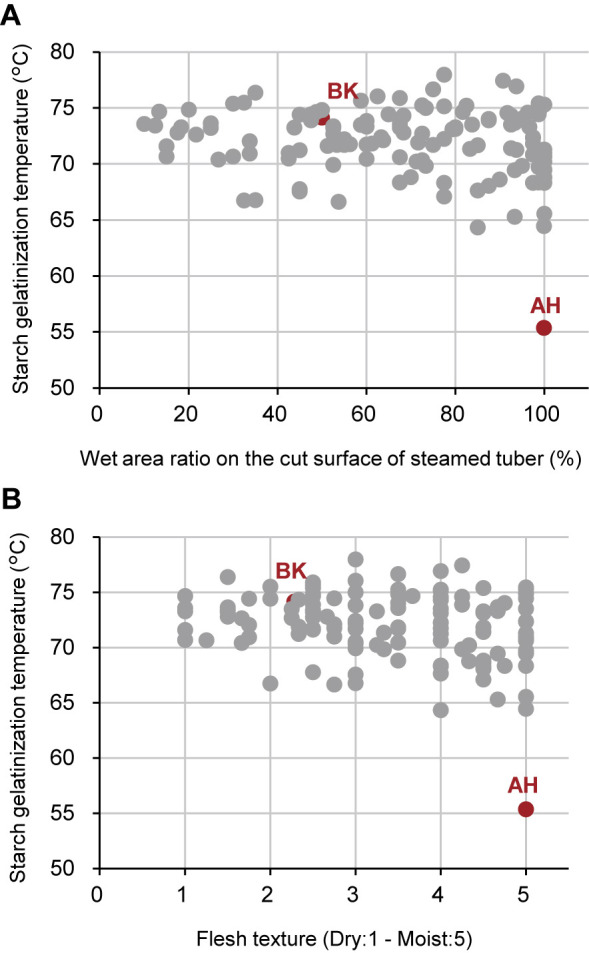
Relationship between steamed tuber texture and starch GT for BK, AH, and ABF_1_ progenies. (A) Relationship between WAR and starch GT. (B) Relationship between flesh texture and starch GT. Values represent averages from tests in 2021 and 2022. ABF_1_ individual lines are depicted as gray dots, while BK and AH are shown as red dots.

The GT of tuber starch was analyzed to explore the relationship between the flesh texture and starch properties. Although AH, a moist-fleshed parent, harbored starch with a low GT of 55.4°C, none of the ABF_1_ progenies possessed starch with such a low temperature. Instead, their GT ranged from 64.3°C to 78.0°C, averaging 72.0°C (Fig. 3). Among the 126 progenies, 31 lines (equivalent to 25%) exhibited GT values surpassing that of BK (74.1°C), yet none of the lines featured temperatures lower than that of AH (55.4°C). Upon investigating the relationships between WAR, flesh texture, and starch GT within ABF_1_ progenies, it was evident that even progenies with elevated WAR and moist flesh texture displayed moderately high GT values. Consequently, starch GT emerged as an independent trait, exhibiting very weak correlations with both WAR and flesh texture (*R*^2^ = 0.03 and *R*^2^ = 0.07, respectively) (Fig. 4).

### Polyploid QTL-seq analysis of WAR and starch GT

A QTL analysis for WAR was conducted to unravel the genetic determinants underlying flesh texture. Recently, we devised a polyploid QTL-seq methodology for QTL detection in polyploid species such as potato and sweetpotato (Yamakawa *et al.* 2021). However, this method entails labor-intensive manual steps to merge the SNP lists. In this study, we introduced a new pipeline, PolyploidQtlSeq, designed to streamline the analysis process by executing a few commands. PolyploidQtlSeq is an extension of the original QTL-seq program (Sugihara *et al.* 2022, Takagi *et al.* 2013), tailored for polyploid F_1_ populations. It accommodates a plexity-adapted simulation based on an F_1_ null distribution, assuming the absence of QTLs, to compute *P* values. The program generates SNP-index and ΔSNP-index plots, along with a list of QTL-deduced variants.

**Fig. 5.**
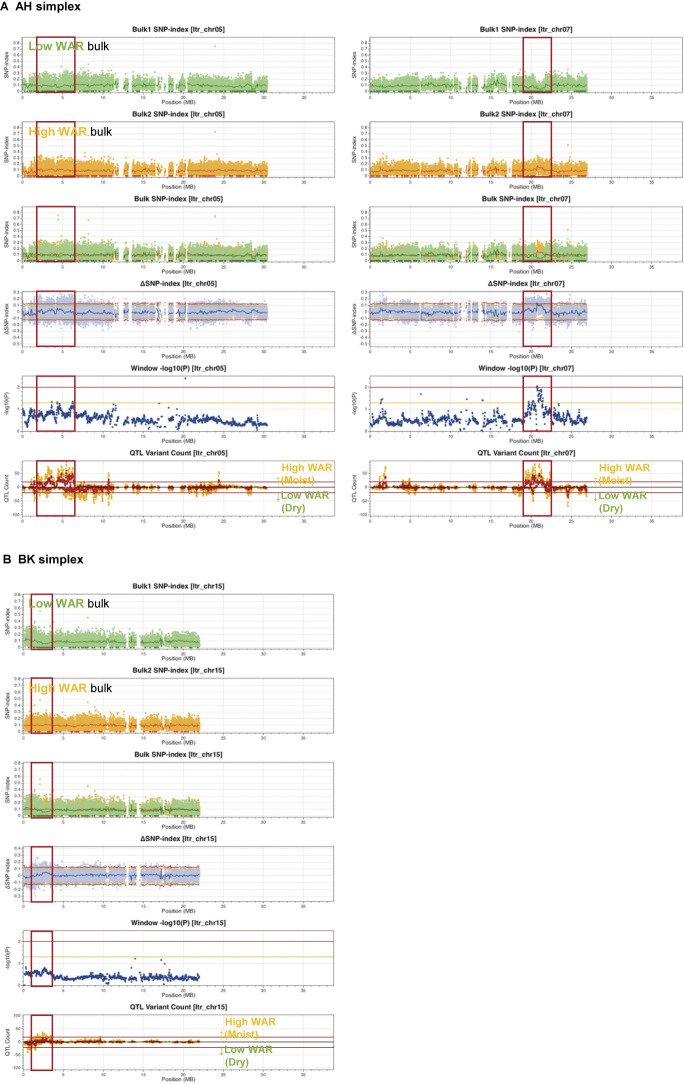
Genomic regions associated with WAR in ABF_1_ progenies. (A) Polyploid QTL-seq analysis using AH-derived simplex variants. (B) Polyploid QTL-seq analysis using BK-derived simplex variants. SNP-index plots of low WAR bulk and high WAR bulk, their superimposed plot, ΔSNP-index plot, window -log_10_*P* plot, and QTL variant count plot are depicted. Dark green and orange dots in the SNP-index plots indicate variants with an SNP-index of 0. Green, red, and blue lines indicate the sliding window average of a 100 kb interval with a 20 kb increment for SNP-index and ΔSNP-index. Window -log_10_*P* plot is the graph plotting average of -log_10_*P* values of all variants included in the sliding window. Orange and red lines on the ΔSNP-index plot and the window -log_10_*P* plot delineate 95% and 99% statistical confidence thresholds under the null hypothesis of no QTLs, respectively. QTL variant count plot is the graph plotting the number of QTL-deduced variants in the sliding window. Orange and red dots on the QTL variant count plot show the number of variants deduced as QTL with 95% and 99% statistical confidence, respectively, and red lines indicate the threshold of 20 for the criteria for the determination of QTL candidate regions. In order to divide variants by the direction of the effect of QTLs, those with positive and negative ΔSNP-index values are plotted upward and downward on the QTL variant count plot, respectively. Candidate regions deduced as QTLs are indicated by red frames. The graphs are depicted for Itr_chr05 and Itr_chr07 in the AH-derived simplex analysis and for Itr_chr15 in the BK-derived simplex analysis. Additional chromosomes are presented in Supplemental Fig. 1.

**Fig. 6.**
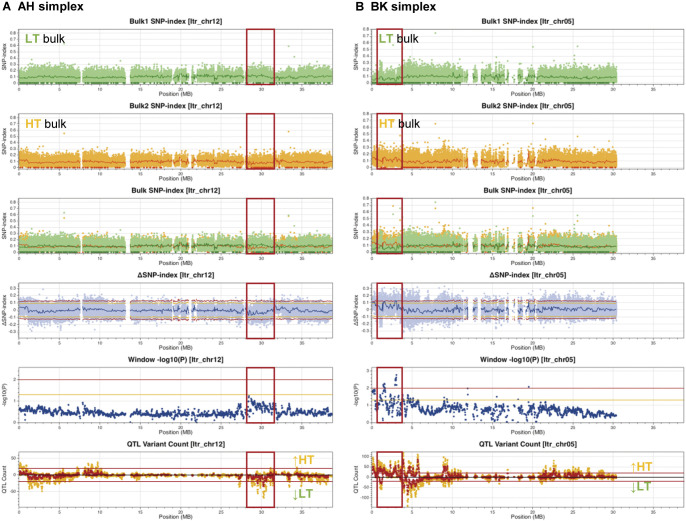
Genomic regions influencing starch GT in ABF_1_ progenies. (A) Polyploid QTL-seq analysis using AH-derived simplex variants. (B) Polyploid QTL-seq analysis using BK-derived simplex variants. SNP-index plots of low-temperature (LT) bulk and high-temperature (HT) bulk, their superimposed plot, ΔSNP-index plot, window -log_10_*P* plot, and QTL variant count plot are depicted similarly to Fig. 5. Red frames indicate candidate regions deduced as QTLs. The graphs are depicted for Itr_chr12 in the AH-derived simplex analysis and for Itr_chr05 in the BK-derived simplex analysis. Additional chromosomes are shown in Supplemental Fig. 2.

For QTL analysis, 10 lines with WARs below 30% and 21 lines with WARs above 95% were utilized to generate low and high WAR bulks, respectively, since the former consisted of lines with flesh texture scores below 2.5 and the latter consisted of those with the scores above 4 (Fig. 1). Polyploid QTL-seq analysis identifies and uses variants, such as SNPs and short In/Dels (less than 72 bp), that are uniquely located on one of the six homoeologous chromosomes in sweetpotato. These variants were designated simplex variants. The progeny resulting from a cross between a parent possessing a simplex variant and another parent devoid of the polymorphism, referred to as a nulliplex, inherits the polymorphism in a 1:1 ratio. Because the SNP indices for the simplex and nulliplex variants are 0.167 and 0, respectively, the expected SNP-index for both progeny bulks in the regions not linked to target genes should be approximately 0.083. However, the presence of a QTL in a region leads to a skewing of the SNP-index for one bulk toward 0.167 owing to the enrichment of simplex progenies, whereas the other bulk’s SNP-index approaches 0 owing to an increased number of nulliplex progenies for the homoeologous chromosome harboring the causative gene. This gives rise to an increase or decrease in the ΔSNP-index from 0 to 0.167 or –0.167. Even in cases where skewed distribution occurs for one homoeologous chromosome, the other chromosomes, which do not harbor the causative gene, are transferred to the progenies without any selection, and SNP-indexes for the variants on those chromosomes are approximately 0.083 for both bulks, resulting in numerous dots of the ΔSNP-index remaining around 0. Because the SNP-index fluctuates upward and downward from the expected value owing to the random reading of homoeologous chromosomes, a certain number of SNPs show SNP-index values exceeding statistical confidence thresholds in the region where an extremely large number of SNPs exist. In these regions, numerous ΔSNP-index dots are dispersed symmetrically above the upper threshold and below the lower threshold. In contrast, in the presence of a QTL, the SNP-index of one bulk is elevated while that of the other bulk approaches 0, leading to ΔSNP-index dots exceeding the threshold solely on one side. Considering such an asymmetrical distribution of ΔSNP-index dots, QTL candidate regions were identified when the region met both of the following criteria: 1) the number of QTL-deduced variants in the 100-kb sliding window, depicted as the QTL variant count graph (Figs. 5, 6), was more than 20 over a 2 Mb region in one direction of QTL effect but not in the other direction, and 2) SNPs whose SNP-index were 0 (indicated by dark green or orange dots in the SNP-index graphs) were clustered over a 2 Mb region in one bulk but not in the other bulk. By applying these criteria, the candidate regions were proposed for Itr_chr05 (2–6 Mb) and Itr_chr07 (19–22 Mb) in the analysis using AH-derived simplexes (Fig. 5A, left panels of Supplemental Fig. 1A). Within these regions, the high-WAR bulk exhibited SNPs with elevated SNP-indexes compared to that of the low-WAR bulk. Furthermore, the low-WAR bulk sample displayed an intense cluster of SNPs with an SNP-index of 0 (Supplemental Table 2). Notably, an asymmetrical distribution of ΔSNP-index dots was observed predominantly above the upper threshold in the positive direction, indicating that these QTLs were likely to increase WAR. Similarly, analysis using BK-derived simplexes indicated a candidate region for Itr_chr15 (2–3 Mb) (Fig. 5B, left panels of Supplemental Fig. 1B). This region coincided with a dense cluster of SNPs featuring an SNP-index of 0 in the low-WAR bulk (Supplemental Table 3). An asymmetrical distribution of ΔSNP-index dots was observed, exceeding the upper threshold asymmetrically, implying that another QTL contributed to the increased WAR.

A QTL analysis was performed for starch GT to elucidate whether starch GT follows a regulatory pattern similar to that of flesh texture. In this analysis, 15 lines with starch GT below 68.5°C and another 15 lines with starch GT above 75°C were selected to generate low-temperature (LT) and high-temperature (HT) bulks, respectively. By applying the same criteria for QTL identification, the analysis of AH-derived simplexes suggested a candidate region for Itr_chr12 (28–31.5 Mb) (Fig. 6A, left panels of Supplemental Fig. 2A). Within this region, the LT bulk exhibited SNPs with higher SNP-indexes compared to the HT bulk, while the HT bulk featured a concentrated cluster of SNPs with an SNP-index of 0 (Supplemental Table 4). An asymmetrical distribution of ΔSNP-index dots was predominantly observed below the lower threshold in the negative direction, indicating the presence of a QTL associated with decreased GT. Although SNPs whose SNP-index is 0 existed in the both bulks similarly, an asymmetrical downward distribution of ΔSNP-index dots was observed around 2 Mb of Itr_chr08 (Supplemental Fig. 2A). Similarly, the analysis utilizing BK-derived simplexes indicated a candidate region for Itr_chr05 (1–4 Mb) (Fig. 6B, left panels of Supplemental Fig. 2B). This region coincided with a dense cluster of SNPs featuring an SNP-index of 0 in the LT bulk (Supplemental Table 5). An asymmetrical distribution of ΔSNP-index dots was observed, exceeding the upper threshold asymmetrically, suggesting a QTL contributing to increased GT. Similarly, an asymmetrical upward distribution of ΔSNP-index dots was observed around 2.5 Mb of Itr_chr06 (Supplemental Fig. 2B).

### Development of DNA markers linked to WAR and starch GT

Given the identification of candidate regions for both WAR and starch GT, linked PCR markers were created at the SNP sites, where SNP indices were close to 0 for one bulk and around 0.167, the values expected for the simplex, for the other bulk (Supplemental Table 6), and the ABF_1_ progenies were genotyped by PCR and agarose gel electrophoresis (Supplemental Fig. 3).

**Fig. 7.**
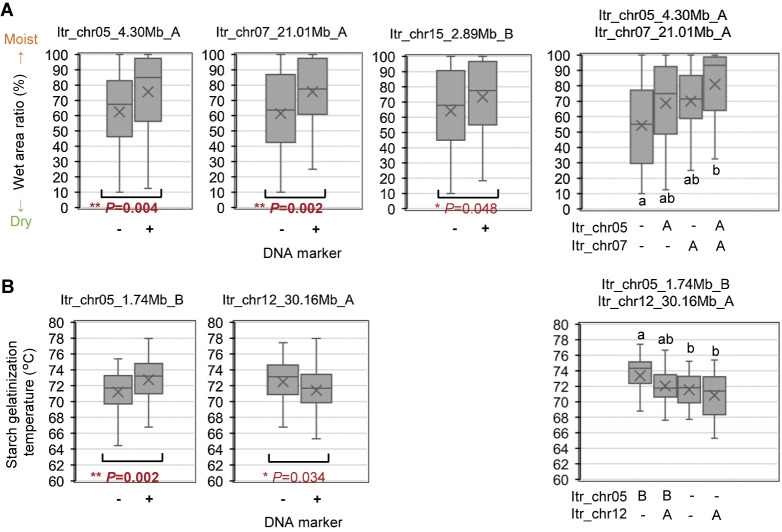
Genotyping using developed DNA markers. (A) Evaluation of WAR-linked SNP markers derived from AH (Itr_chr05_4.30Mb_A, Itr_chr07_21.01Mb_A) and BK (Itr_chr15_2.89Mb_B) simplex variants. Relationships between marker genotypes (+; presence or –; absence) and WAR are shown for the ABF_1_ population (*n* = 123). The effect of the combination of AH markers is indicated. (B) Evaluation of starch GT-linked SNP markers from BK (Itr_chr05_1.74Mb_B) and AH (Itr_chr12_30.16Mb_A) simplex variants. Relationships between marker genotypes and starch GT are presented. The effect of the combination of the markers is shown. The box plots show the minimum, first quartile, median, third quartile and maximum values. The ‘x’ marks in the box plots are the mean values. Amplification with control *SSII* primers was verified. A: AH-derived simplex, B: BK-derived simplex, -: Absence of respective simplex. Different lower-case letters denote significant differences at *P* < 0.05.

Three markers linked to the WAR were developed: Itr_chr05:4298933/G; Itr_chr07:21010941/A; and Itr_chr15:2888152/T. These markers targeted the AH-specific A to G SNP on chromosome Itr_chr05 at 4,298,933 bp, the AH-specific C to A SNP on chromosome Itr_chr07 at 21,010,941 bp, and the BK-specific G to T SNP on chromosome Itr_chr15 at 2,888,152 bp (Supplemental Tables 2, 3). These markers yielded distinctive fragments in the intended cultivar but not in the other cultivar. These were used to genotype 123 ABF_1_ progenies. Individuals carrying each AH-derived allele detected using Itr_chr05:4298933/G (Itr_chr05_4.30Mb_A) and Itr_chr07:21010941/A (Itr_chr07_21.01Mb_A) displayed significantly higher WAR than those lacking these alleles (Fig. 7A, Supplemental Table 7). Interestingly, although BK exhibited a drier flesh texture and lower WAR than AH (Fig. 1), individuals retaining a BK-derived allele detected using Itr_chr15:2888152/T (Itr_chr15_2.89Mb_B) showed a higher WAR than those without the allele. Furthermore, an additive effect of Itr_chr05_4.30Mb_A and Itr_chr07_21.01Mb_A was observed. In the absence of these alleles, the average WAR is 54.4%. Incorporating either allele increased it to approximately 70%, and incorporating both alleles further increased it to 81.0%.

Four markers linked to starch GT were developed: Itr_chr05:1741028/A; Itr_chr06:2413793/G; Itr_chr08:2039510/T; and Itr_chr12:30157377/G. These markers targeted the BK-specific G to A SNP on chromosome Itr_chr05 at 1,741,028 bp, the BK-specific C to G SNP on chromosome Itr_chr06 at 2,413,793 bp, the AH-specific C to T SNP on chromosome Itr_chr08 at 2,039,510 bp, and the AH-specific A to G SNP on chromosome Itr_chr12 at 30,157,377 bp (Supplemental Tables 4, 5). Similar to the WAR-linked markers, these markers yielded specific fragments in the intended cultivar but not in the other cultivar and were used for genotyping 123 ABF_1_ progenies. Individuals harboring a BK-derived allele detected using Itr_chr05:1741028/A (Itr_chr05_1.74Mb_B) exhibited significantly higher GT than those lacking this allele (Fig. 7B, Supplemental Table 7). Conversely, individuals retaining an AH-derived allele detected using Itr_chr12:30157377/G (Itr_chr12_30.16Mb_A) showed a lower GT than those without the allele. However, the effects of the other two alleles, Itr_chr06:2413793/G (Itr_chr06_2.41Mb_B) and Itr_chr08:2039510/T (Itr_chr08_2.04Mb_A), were not statistically significant (Supplemental Fig. 4). Moreover, the combined effects of Itr_chr05_1.74Mb_B and Itr_chr12_30.16Mb_A were explored. The presence of Itr_chr05_1.74Mb_B raised GT to 73.4°C in the absence of Itr_chr12_30.16Mb_A. However, the incorporation of Itr_chr12_30.16Mb_A attenuated this GT-elevating effect. Similarly, the presence of Itr_chr12_30.16Mb_A lowered GT to 70.8°C in the absence of Itr_chr05_1.74Mb_B. Retaining Itr_chr05_1.74Mb_B mitigated this GT-reducing effect.

### Starch-lytic enzyme activities of AH, BK, and their F_1_ progenies

Given the connection between the conversion of flesh texture from dry to moist and increased sugar content during tuber storage (Nishinaka *et al.* 2020), the activities of the enzymes involved in degrading storage starch into sugars, α-amylase and β-amylase, were compared between AH and BK. The decomposition of storage starch into sugars accompanied by the increased activity of α-amylase was reported at 138 days after harvest (Takahata *et al.* 1995), and the expression of an α-amylase gene was up-regulated in the moist cultivar ‘Beniharuka’ after 12 weeks of storage (Suematsu *et al.* 2019). Therefore, the activities were determined after three months of storage. While the moist cultivar AH exhibited 1.5-fold higher activity for both enzymes compared to the dry cultivar BK, the heat stability of β-amylase was 65°C in both cultivars (Supplemental Fig. 5). This disparity suggests that differences in the extent of starch-lytic enzyme activity contribute to variance in WAR and flesh texture. Nevertheless, among the ABF_1_ lines, the activity levels of these enzymes remained consistent regardless of the presence or absence of WAR-linked alleles, such as Itr_chr05_4.30Mb_A and Itr_chr07_21.01Mb_A (Supplemental Fig. 6). Moreover, it is noteworthy that the activities of α-amylase and β-amylase did not exhibit significant correlations with either WAR (*R*^2^ = 0.008 and *R*^2^ = 0.008, respectively) or flesh texture (*R*^2^ = 0.011 and *R*^2^ = 0.008, respectively). Consequently, it appears that the flesh texture is not directly linked to starch-lytic enzyme activities.

## Discussion

In the present study, we developed a novel pipeline for polyploid QTL-seq. The polyploid QTL-seq method enables the swift identification of QTLs in polyploid species, such as sweetpotato and potato (Yamakawa *et al.* 2021). However, the original protocol relied on a single bulk search for clusters of SNPs with an SNP-index of 0 to identify candidate regions. This approach may not be suitable for multigenic traits, as SNP-indexes for various QTL regions may not always be 0. In contrast, the new pipeline used an ΔSNP-index, which reflects the SNP-indexes of both bulks and searches for the clusters of SNPs whose SNP-indexes are significantly different between the two bulks, enabling the identification of multiple QTLs simultaneously.

Using this new pipeline, we investigated the genomic regions responsible for sweetpotato tuber flesh texture and starch GT. Given the challenge of consistently evaluating flesh texture owing to the requirement for trained gustatory senses, we visually inspected the WAR of the steamed tuber section surface, which exhibited a strong correlation with flesh texture (Fig. 1). The ABF_1_ progeny generated by crossing the moist-fleshed cultivar AH and the dry-fleshed cultivar BK demonstrated a broad spectrum of flesh texture and WAR (Fig. 2). Through polyploid QTL-seq analysis and subsequent genotyping with allele-specific DNA markers, we identified three QTLs associated with WAR on chromosomes Itr_chr05, Itr_chr07, and Itr_chr15 (Fig. 5). Interestingly, all identified alleles were associated with an increase in WAR, including two alleles (Itr_chr05_4.30Mb_A and Itr_chr07_21.01Mb_A) in AH and one allele (Itr_chr15_2.89Mb_B) in BK (Fig. 7). Conversely, the ABF_1_ population displayed a narrower distribution of starch GT skewed toward the value of BK (Fig. 3). Although AH displayed starch with a GT approximately 20°C lower than that of BK, none of the F_1_ progenies displayed such reduced GT. Instead, they exhibited moderate-to-high GT values. Furthermore, starch GT exhibited no correlation with either WAR or flesh texture (Fig. 4). Correspondingly, a distinct set of QTLs was detected for the starch GT on chromosomes Itr_chr05 and Itr_chr12 (Fig. 6). Notably, the Itr_chr05_1.74Mb_B allele from BK increased GT, whereas the Itr_chr12_30.16Mb_A allele from AH decreased GT (Fig. 7). Consequently, it is evident that flesh texture and starch GT are governed by different genetic factors. We successfully identified the effective alleles for the major QTLs and developed DNA markers linked to both WAR and starch GT (Supplemental Table 6). Utilizing these WAR-linked markers, particularly Itr_chr05:4298933/G and Itr_chr07:21010941/A in combination, enabled immediate selection of dry- or moist-fleshed progenies from the ABF_1_ population without the need for extensive flesh texture testing.

Starch GT has previously been associated with the starch synthase gene *SSII* (Kitahara *et al.* 2011, Takahata *et al.* 2010), and genetic studies demonstrated that null mutation of a single recessive locus is required to achieve a 20°C reduction in GT (Katayama *et al.* 2015). However, the reduction in GT observed in the ABF_1_ progenies was less than 10°C compared with BK (Figs. 3, 4). This discrepancy is consistent with observations from progenies derived from a cross between ‘Quick Sweet’ and ‘Koganesengan’, where ‘Quick Sweet’ possesses a null allele, and ‘Koganesengan’ has more than tetraplex of active alleles (Katayama *et al.* 2015). This suggests that the fluctuation in GT within the present population may be influenced by factors other than *SSII*. A gene homologous to sweetpotato *SSII* (AF068834; Takahata *et al.* 2010), Itr_chr05CG05970, is located at 4.2 Mb (4,203,246 to 4,208,182 bp) on chromosome Itr_chr05 in the *I. trifida* reference genome used here (https://doi.org/10.1101/2022.12.25.521700). AH harbors a 2-bp insertion in its 8th exon at 4,203,819 bp, similar to the case observed in ‘Quick Sweet’ (Takahata *et al.* 2010). Considering its SNP-index of 0.43 (89 reads with insertion among the total of 207 mapped reads) on the NGS read of AH, AH harbors the deleterious mutation in the duplex or triplex, while BK does not (no reads with insertion among the total of 172 mapped reads). Such multiplex polymorphisms are not encompassed within our polyploid QTL-seq analysis, but this mutation was not enriched in the low GT bulks (with SNP-indexes of 0.23 [38 reads with insertion among the total of 166 mapped reads] and 0.30 [54 reads with insertion among the total of 178 mapped reads] for low and high GT bulks, respectively). Consequently, the QTL identified on chromosome Itr_chr05 in the present study may involve alleles other than *SSII* that exert moderate effects on GT. Alternatively, it is plausible that BK-derived *SSII* alleles with varying levels of activity or expression present in more than duplex might collectively contribute in a dose-dependent manner.

To investigate the factors underlying flesh texture, we examined the activities of the starch-lytic enzymes α-amylase and β-amylase, which are encoded by at least 19 and 34 genes in the sweetpotato genome, respectively (https://doi.org/10.1101/2022.12.25.521700). This investigation was prompted by the observation that the conversion of the flesh texture from dry to moist during storage coincided with an increase in sugar content (Nishinaka *et al.* 2020). Although AH, the moist-fleshed parent exhibited 1.5-fold higher activities of both α-amylase and β-amylase than the dry-fleshed parent BK (Supplemental Fig. 5), the activities of these enzymes were not linked to the presence or absence of WAR-linked alleles such as Itr_chr05_4.30Mb_A and Itr_chr07_21.01Mb_A (Supplemental Fig. 6). Consequently, flesh texture appears not directly related to starch-lytic enzyme activities. However, since the activities were determined after three months of storage in the present study, the involvement of amylases before and immediately after harvest is not excluded.

Nakamura *et al.* (2010, 2015, 2018) suggested a correlation between the flesh texture of steamed tubers and the starch content of raw tubers. Higher starch contents tended to result in dry textures after steaming, whereas lower starch contents led to moist textures. Haque *et al.* (2023) conducted QTL analysis for tuber starch content using F_1_ progenies from a cross between a high-starch content cultivar, Konaishin, and a low-starch content cultivar, Akemurasaki, and identified a QTL near the starch synthase gene *IbGBSSI* on chromosome Itr_chr15. Moreover, they found that starch content correlated with the expression level of *IbGBSSI*. In our study, a QTL for tuber texture was detected on the same chromosome (Figs. 5, 7). However, the candidate region was located at 2–3 Mb on the chromosome, and the identified allele, Itr_chr15_2.89Mb_B, was slightly different from the corresponding *GBSS* gene, Itr_chr15CG07050 (5,991,292–5,994,559 bp of the *I. trifida* reference genome). Notably, by comparing the mapped read data between AH and BK, we did not identify any polymorphism causing detrimental effects on the encoded peptide, such as nonsense mutations, codon frameshifts, or splicing acceptor/donor site mutations within the starch synthase gene, although 5 missense and 22 silent SNPs were found. The precise relationship between the WAR-linked alleles and tuber starch content requires further investigation.

In the present study, simplex variants were solely utilized for QTL analyses. Therefore, alleles retained in other than simplex, such as duplex and triplex are not included in the analyses. For both WAR and GT cases, the transgressive segregation was detected (Figs. 2, 3). In the WAR population, 24% of the progenies displayed lower WAR than BK. This result indicated that there should be at least one QTL that decreases WAR derived from the other parent, AH. However, all QTLs detected in the analysis with simplex variants have effects on increasing WAR. In the GT population, 25% of the progenies exhibited higher GT values than BK, which indicated there must be at least one QTL that increases GT derived from the other parent, AH. However, such QTL was not detected in the analysis with simplex variants. To explain these transgressive segregations, the same datasets were analyzed with parameters adjusted for QTL detection with each of duplex and triplex SNPs (Supplemental Figs. 1, 2; middle and right panels). Possible candidate regions of WAR-decreasing QTL, as determined by an asymmetric distribution of ΔSNP-index dots, were detected at 23 Mb of Itr_chr07 and 23 Mb of Itr_chr09 in the analyses with AH-derived duplex and triplex variants (Supplemental Fig. 1). Similarly, a possible candidate region of GT-increasing QTL was detected at 6 Mb of Itr_chr15 in the analyses with AH-derived duplex and triplex variants (Supplemental Fig. 2). Although the distinction between duplex and triplex variants is not perfect with the sequence data amounts in the present study due to the overlapped parameter setting, these duplexed or triplexed regions might be involved in the transgressive segregation. To determine the effect of these QTLs, their allele dosages should be verified in each of the F_1_ progenies by quantitative genotyping methods such as amplicon sequence, KASP assay, fragment analysis, or digital PCR, that might lead to the development of additional DNA markers for WAR and GT.

In conclusion, we refined the polyploid QTL-seq pipeline to identify multiple quantitative QTLs in polyploid crops. The pipeline is accessible at GitHub (https://github.com/TatsumiMizubayashi/PolyploidQtlSeq). Applying this approach to sweetpotato, we identified QTLs governing the flesh texture-correlated trait, WAR, and developed linked DNA markers. These findings provide a path toward breeding sweetpotato cultivars with desired flesh textures.

## Author Contribution Statement

HY conceived the research project and designed the experiments. MT created the sweetpotato F_1_ population. TM developed the polyploid QTL-seq pipeline. HY conducted all the experiments, polyploid QTL-seq, and DNA marker analyses and wrote the manuscript.

## Supplementary Material

Supplemental Figures

Supplemental Table 1

Supplemental Table 2

Supplemental Table 3

Supplemental Table 4

Supplemental Table 5

Supplemental Table 6

Supplemental Table 7
