# Supplementary material for: Polyploid QTL-seq revealed multiple QTLs controlling steamed tuber texture and starch gelatinization temperature in sweetpotato
Source: Breed Sci. 2024 Feb 29;74(2):103–13. doi: 10.1270/jsbbs.23060 (PMC11442106; doi:10.1270/jsbbs.23060)
Supplement: Supplementary file 1 — Supplemental Figures [file 74_103_s1.pdf]

doi: 10.1270/jsbbs.23060

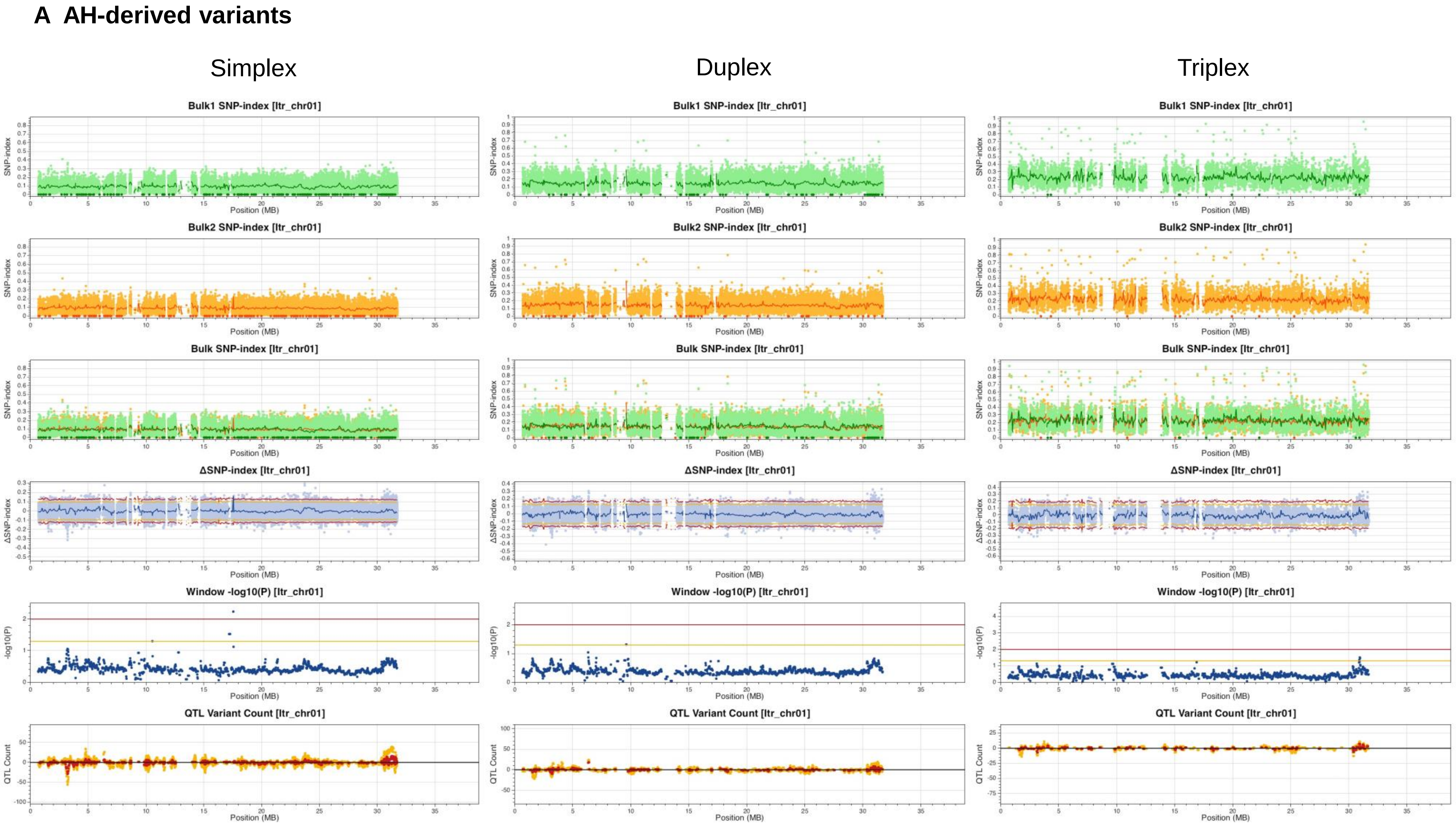

Supplemental Fig. 1. Genomic regions governing WAR in ABF1 progenies. (A) Polyploid QTL-seq analysis using each of AH-derived simplex, duplex and triplex variants. (B) Polyploid QTL-seq analysis using each of BK-derived simplex, duplex and triplex variants. SNP-index plots of low WAR bulk and high WAR bulk, their superimposed plot, ΔSNP-index plot, window -log$_{10}P$ plot, and QTL variant count plot are depicted similarly to that in Fig. 5. Red frames indicate candidate regions for QTLs.

A AH-derived variants

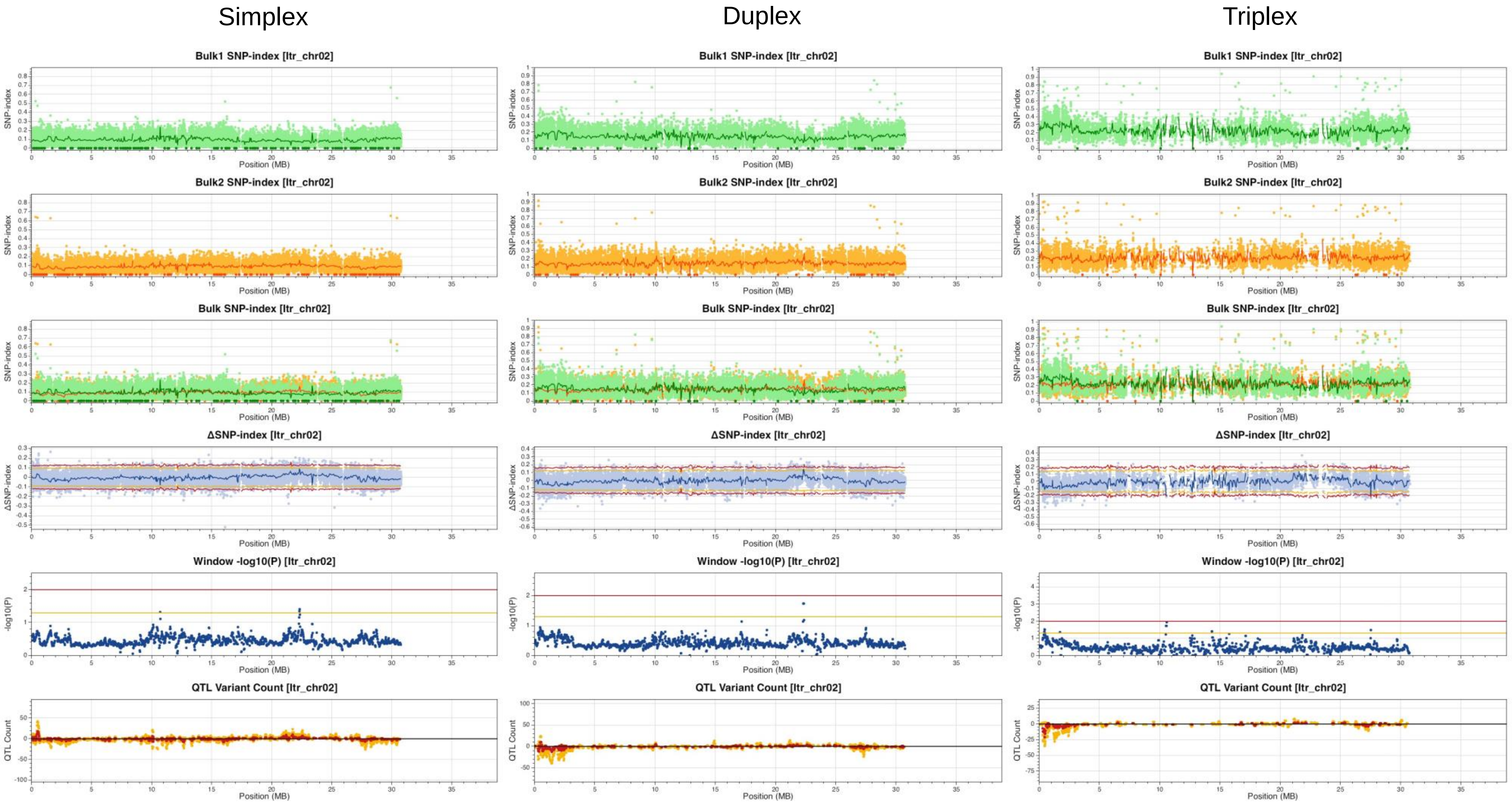

Supplemental Fig. 1. (continued)

A AH-derived variants

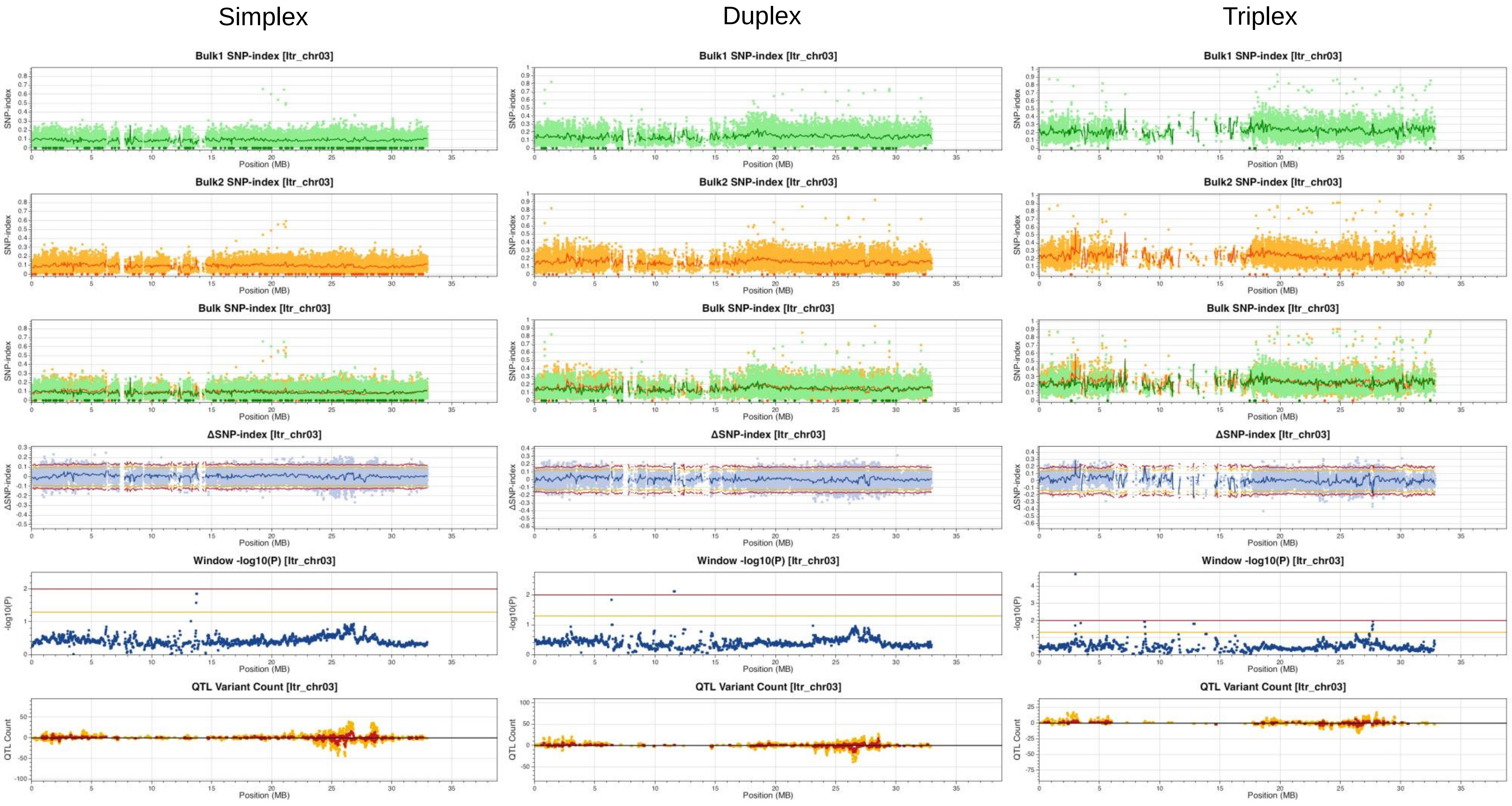

Supplemental Fig. 1. (continued)

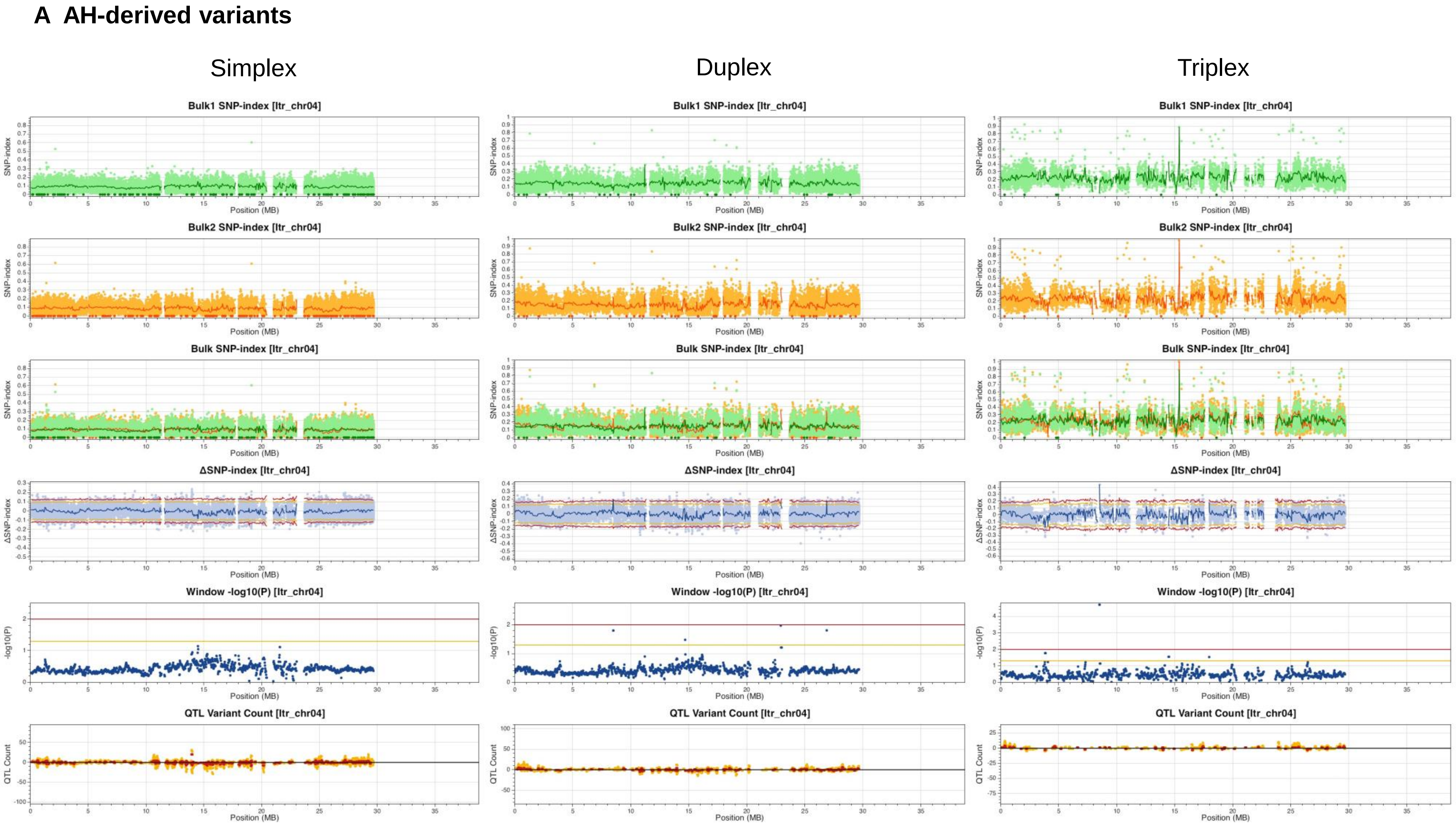

Supplemental Fig. 1. (continued)

A AH-derived variants

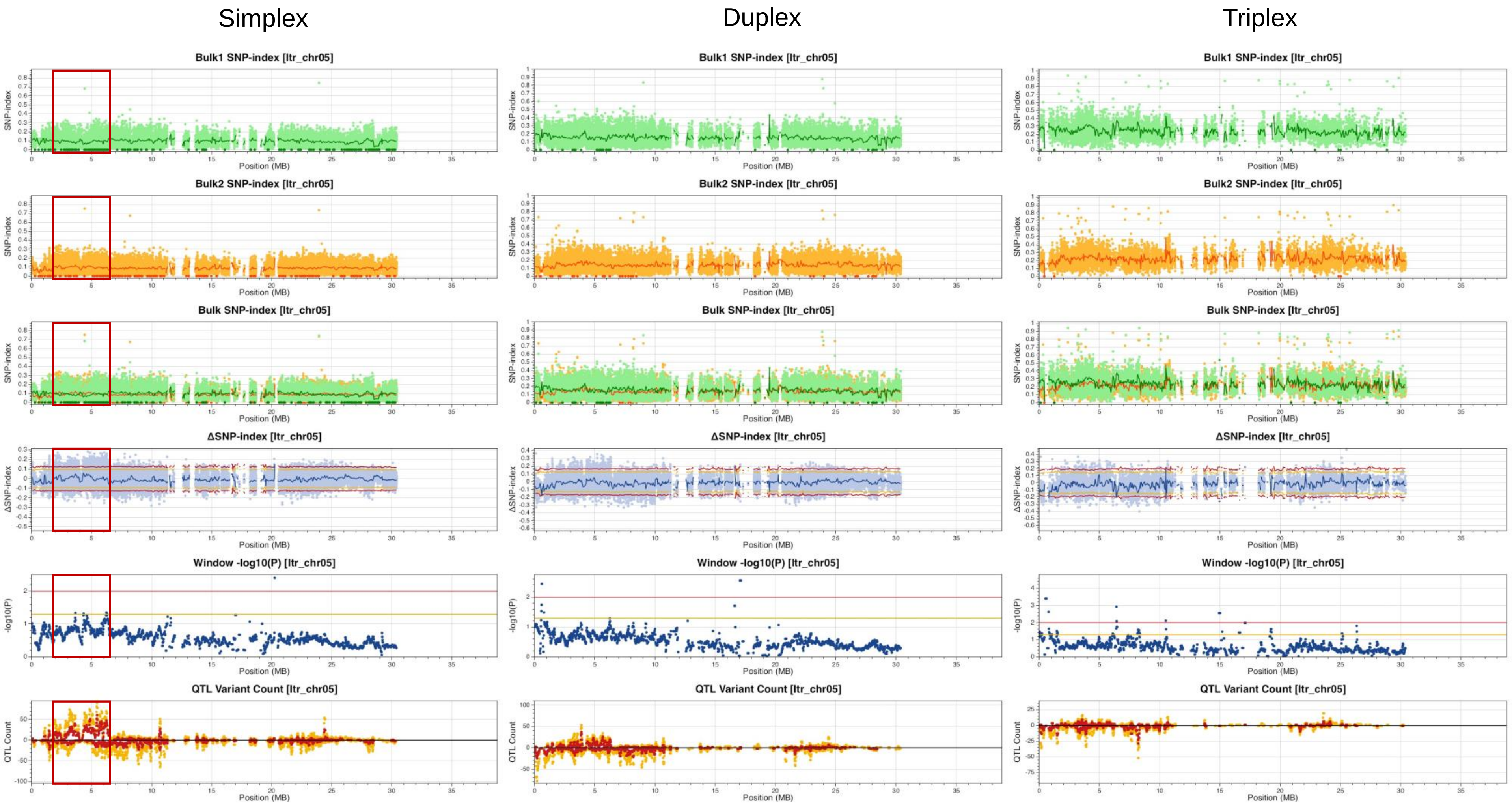

Supplemental Fig. 1. (continued)

A AH-derived variants

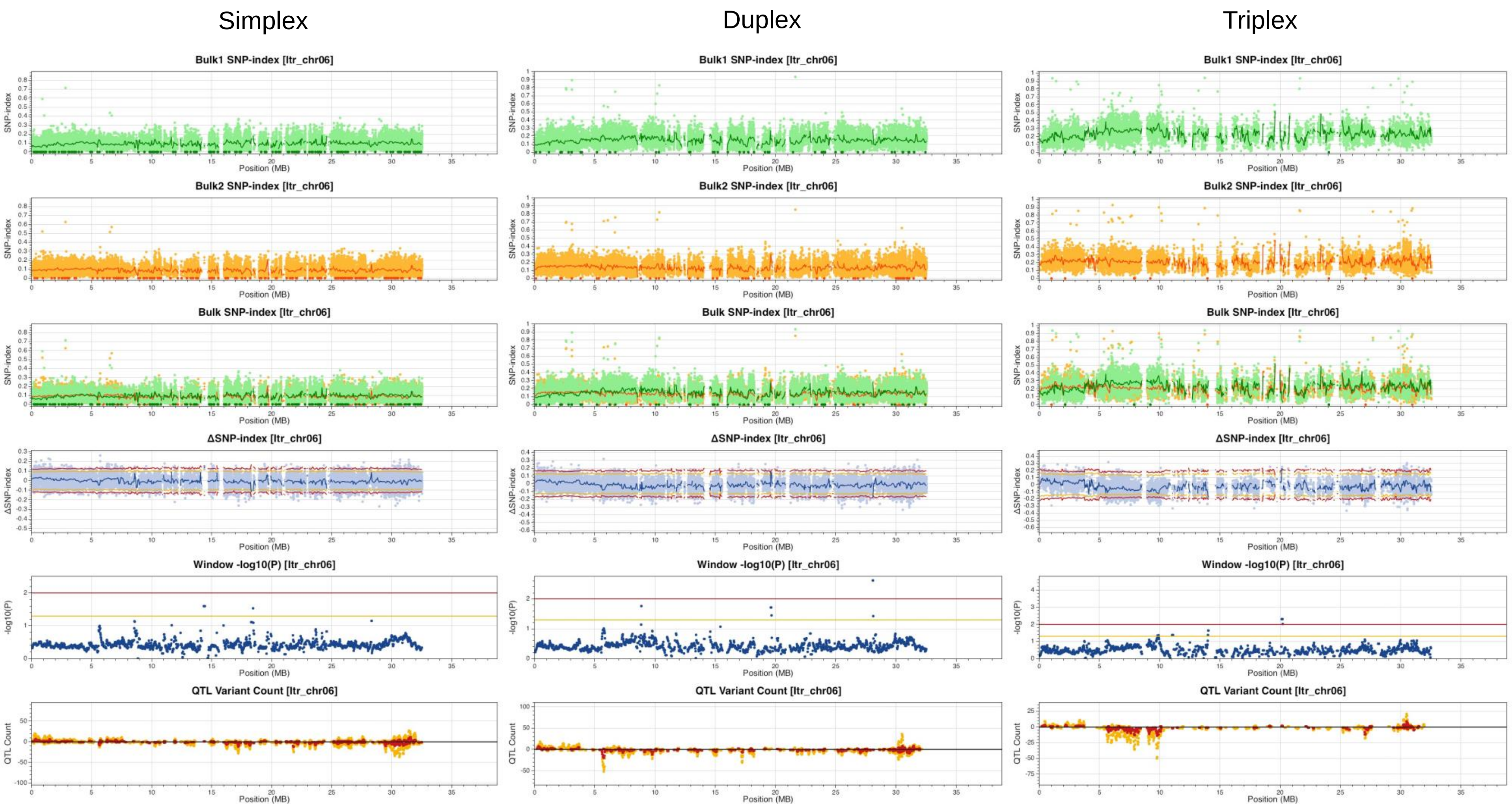

Supplemental Fig. 1. (continued)

A AH-derived variants

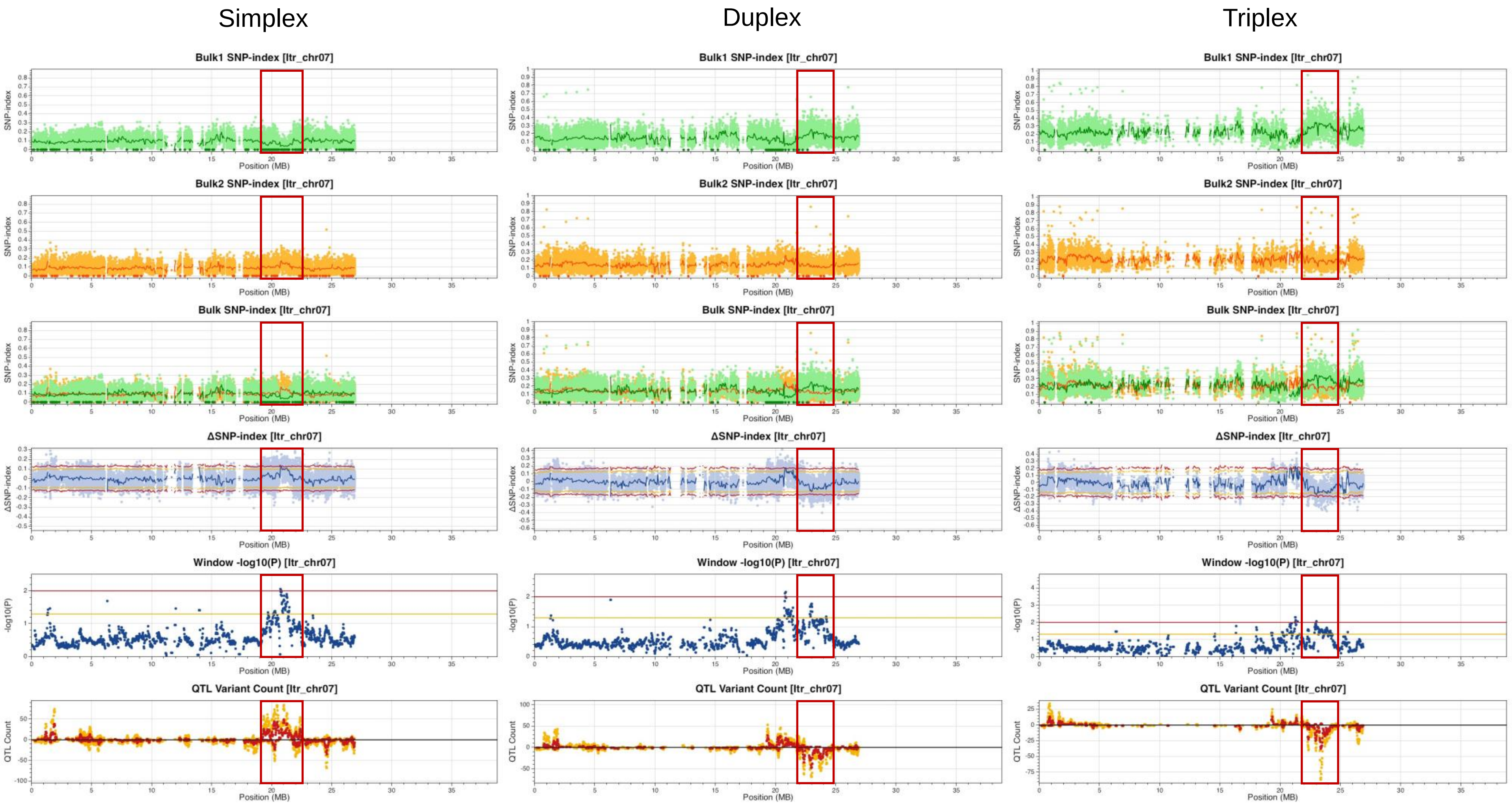

Supplemental Fig. 1. (continued)

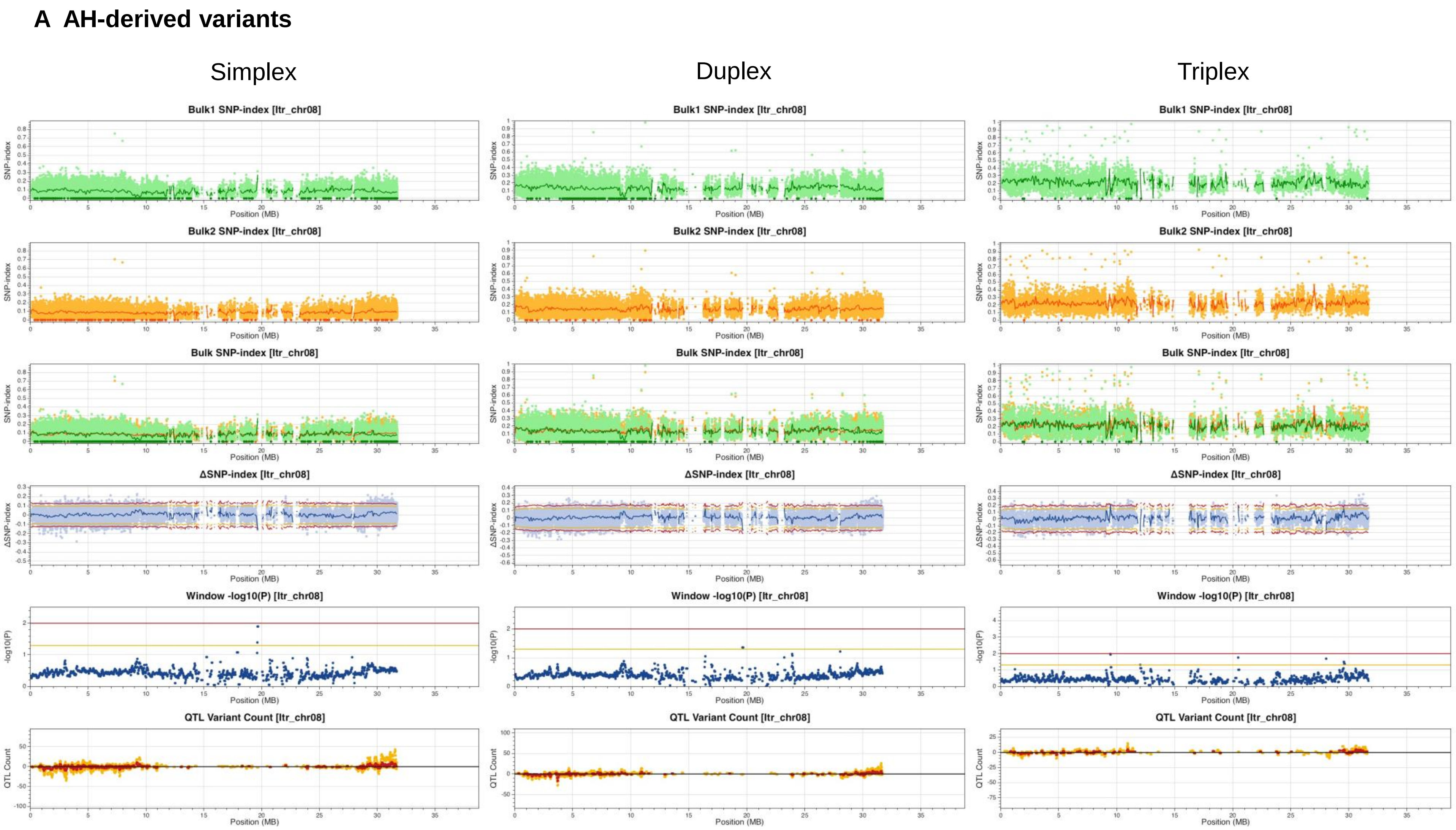

Supplemental Fig. 1. (continued)

A AH-derived variants

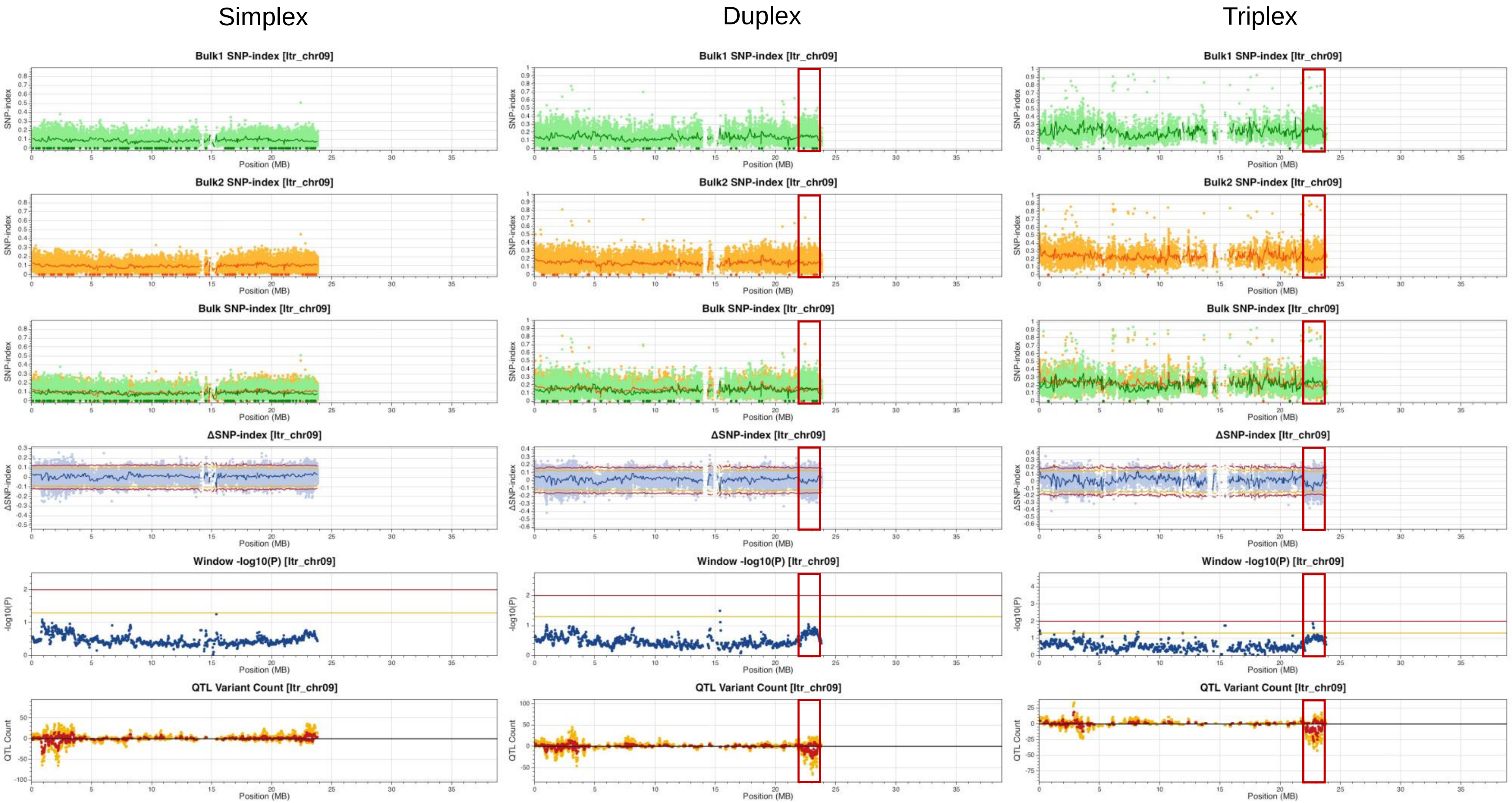

Supplemental Fig. 1. (continued)

A AH-derived variants

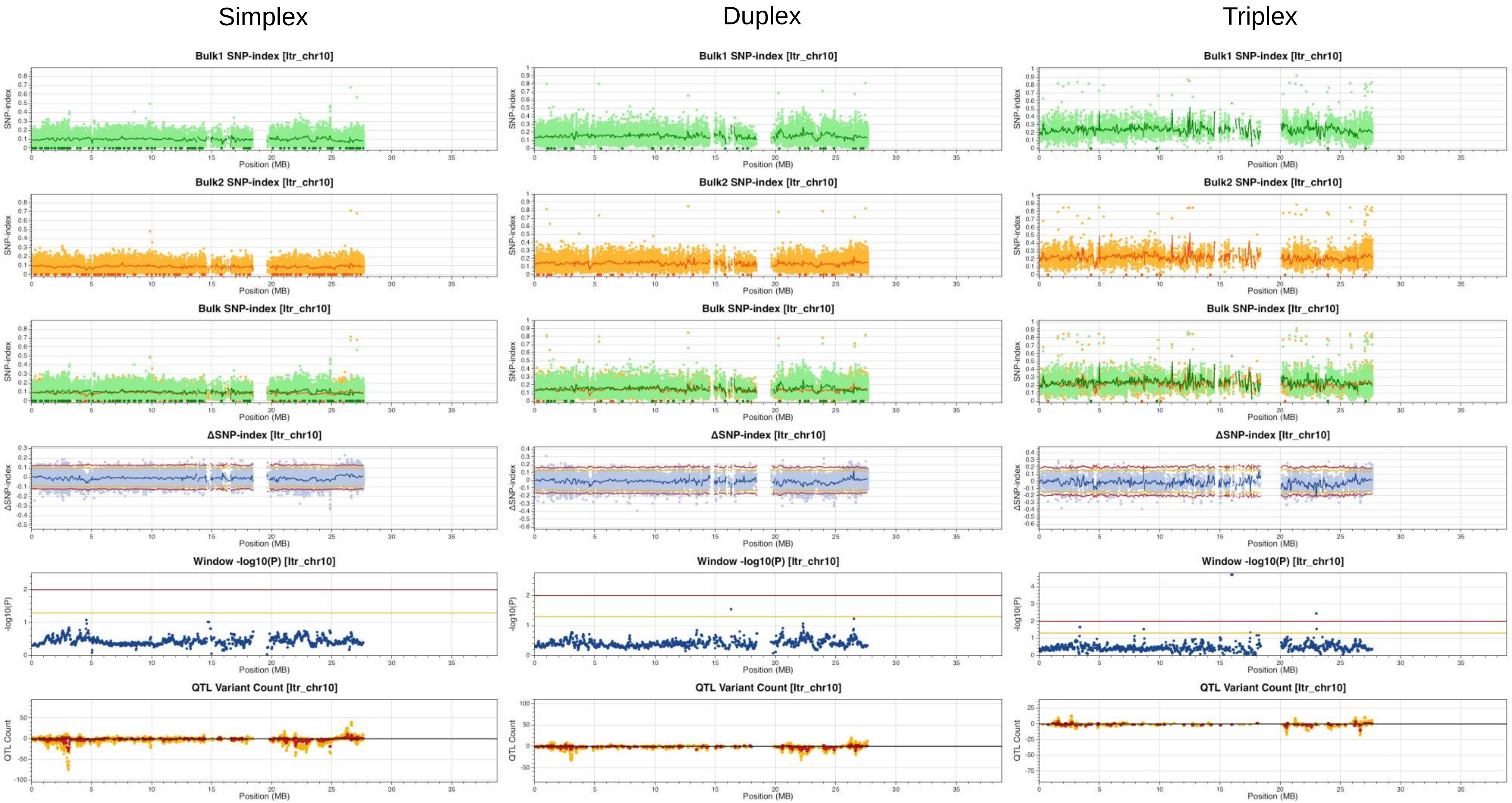

Supplemental Fig. 1. (continued)

A AH-derived variants

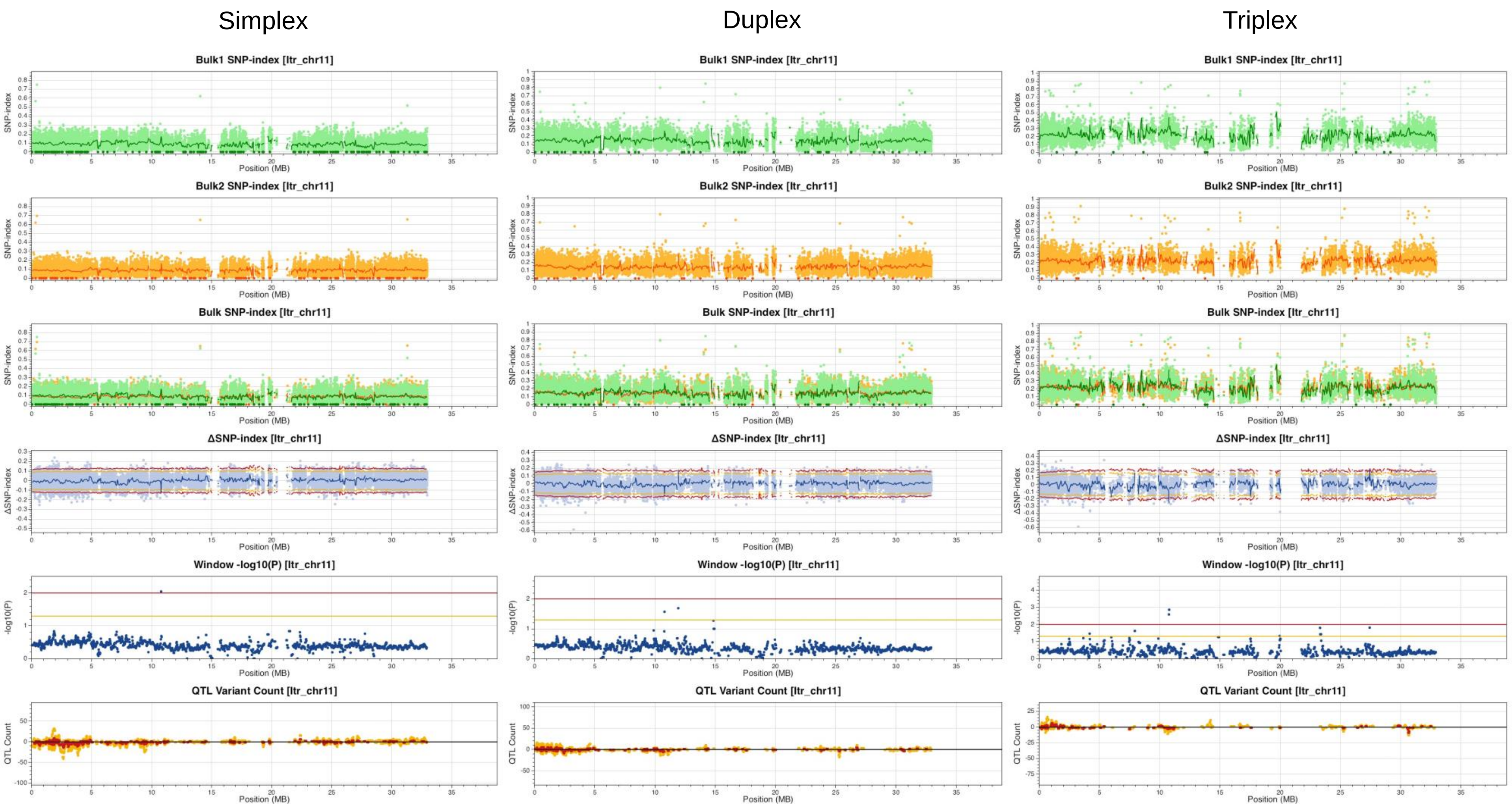

Supplemental Fig. 1. (continued)

A AH-derived variants

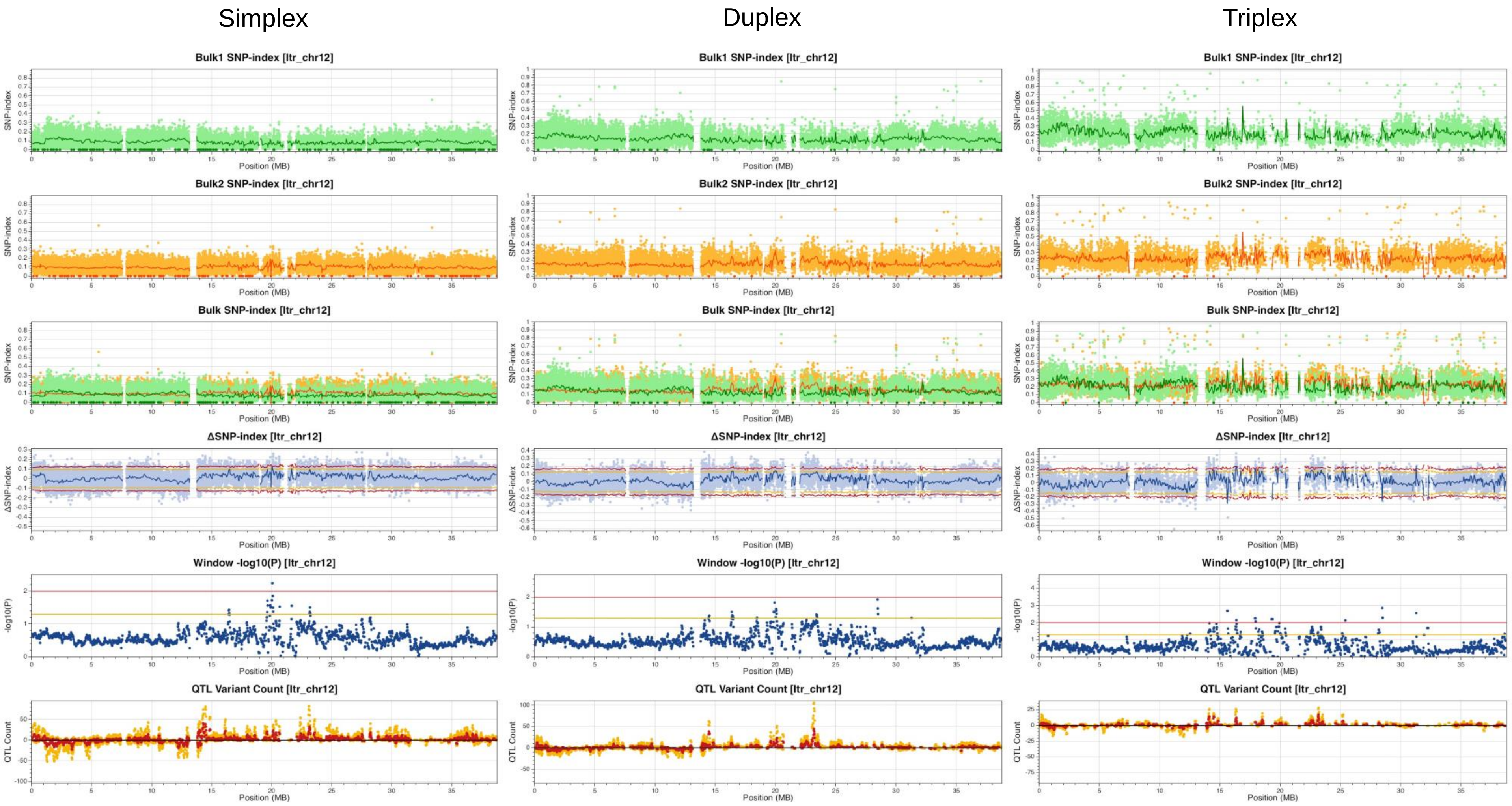

Supplemental Fig. 1. (continued)

A AH-derived variants

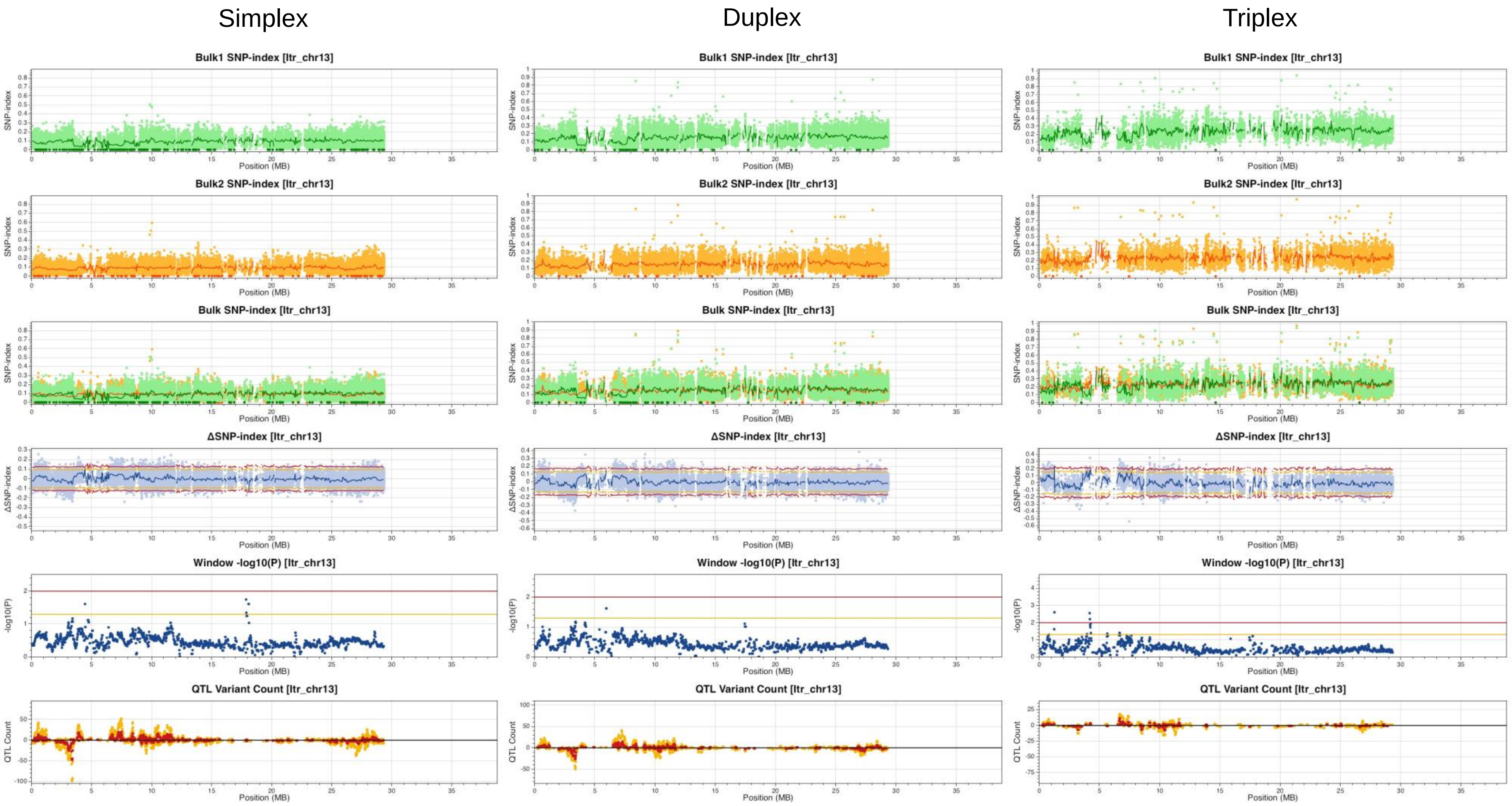

Supplemental Fig. 1. (continued)

A AH-derived variants

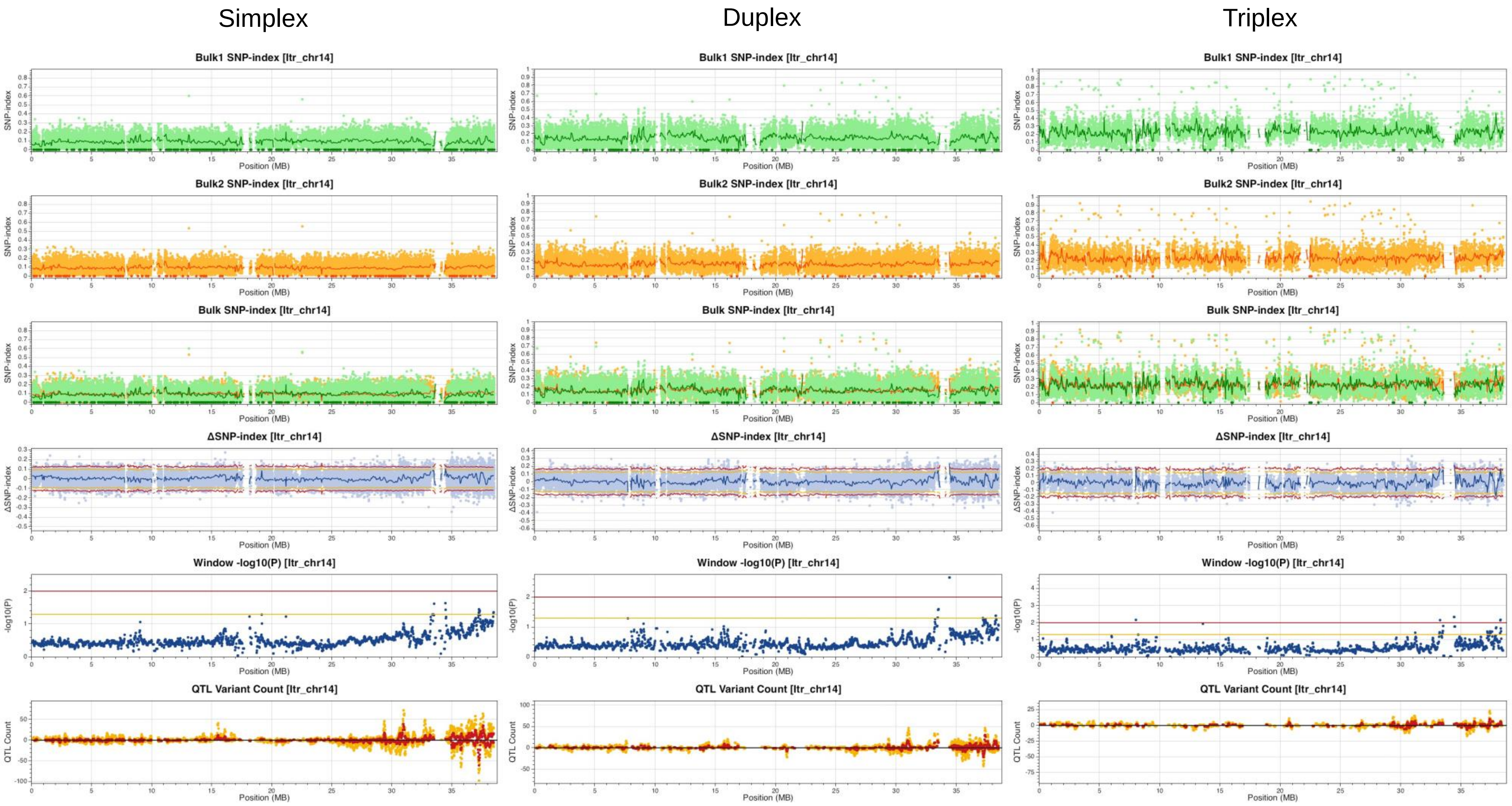

Supplemental Fig. 1. (continued)

A AH-derived variants

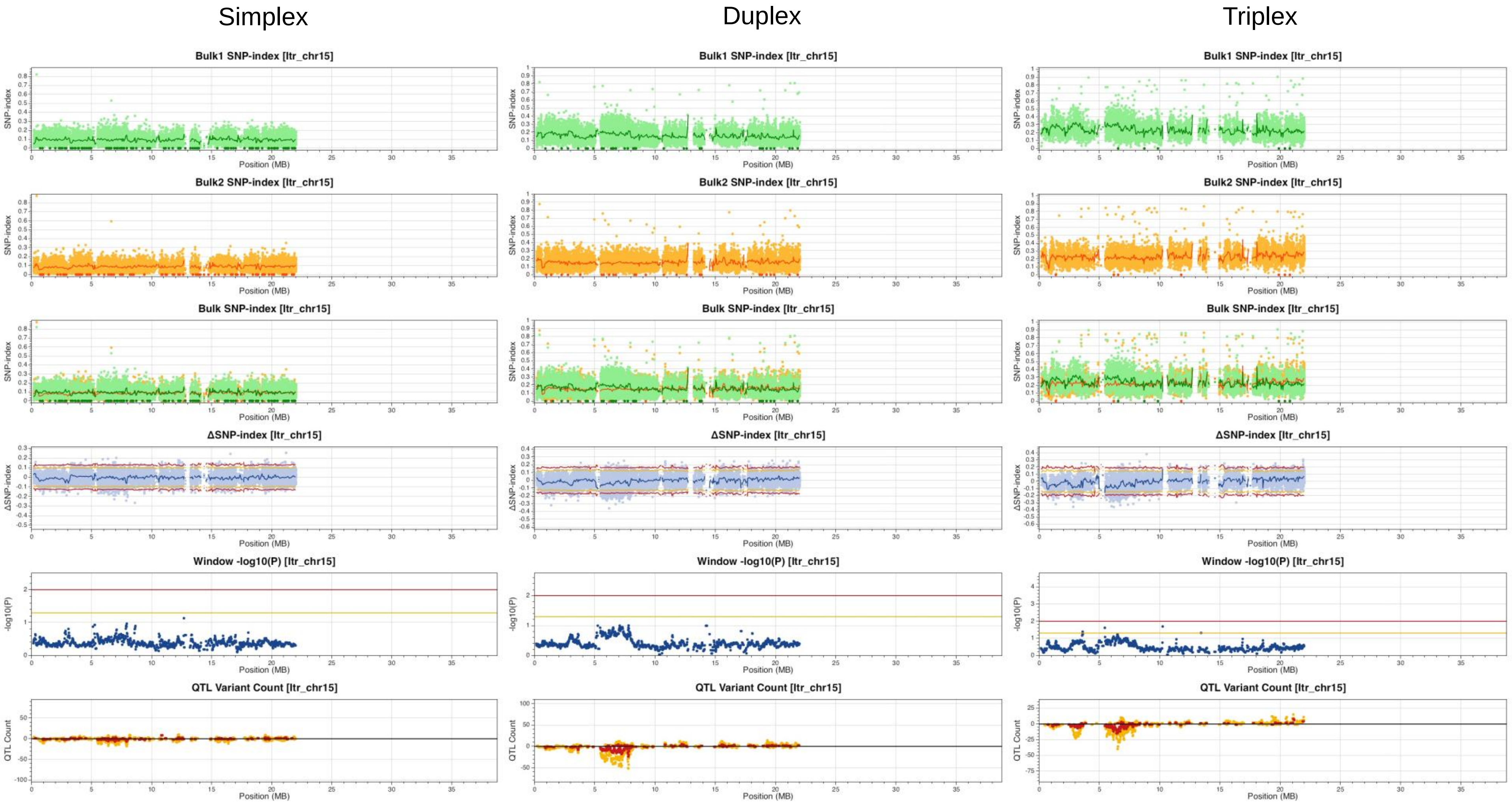

Supplemental Fig. 1. (continued)

B BK-derived variants

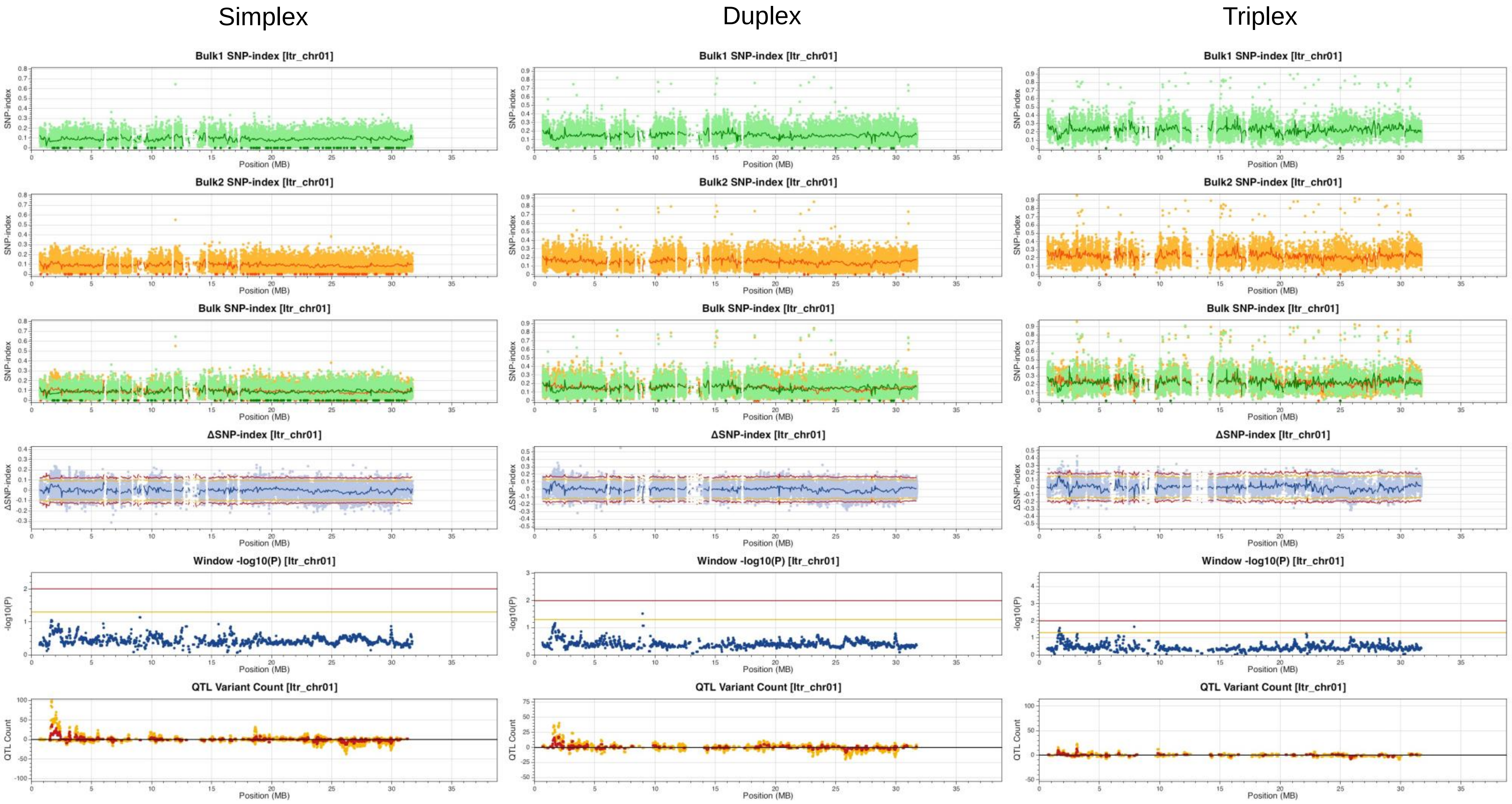

Supplemental Fig. 1. (continued)

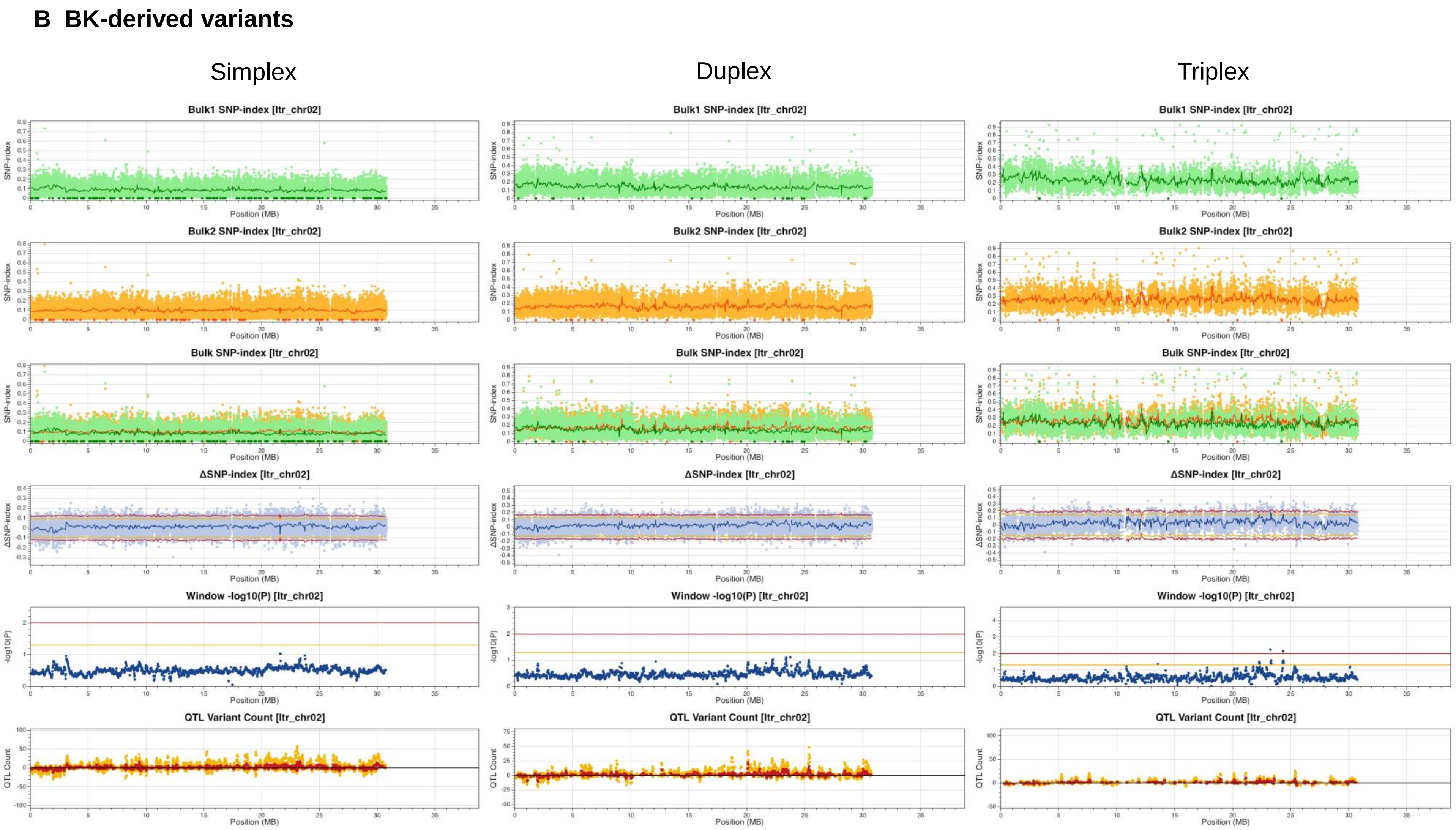

Supplemental Fig. 1. (continued)

B BK-derived variants

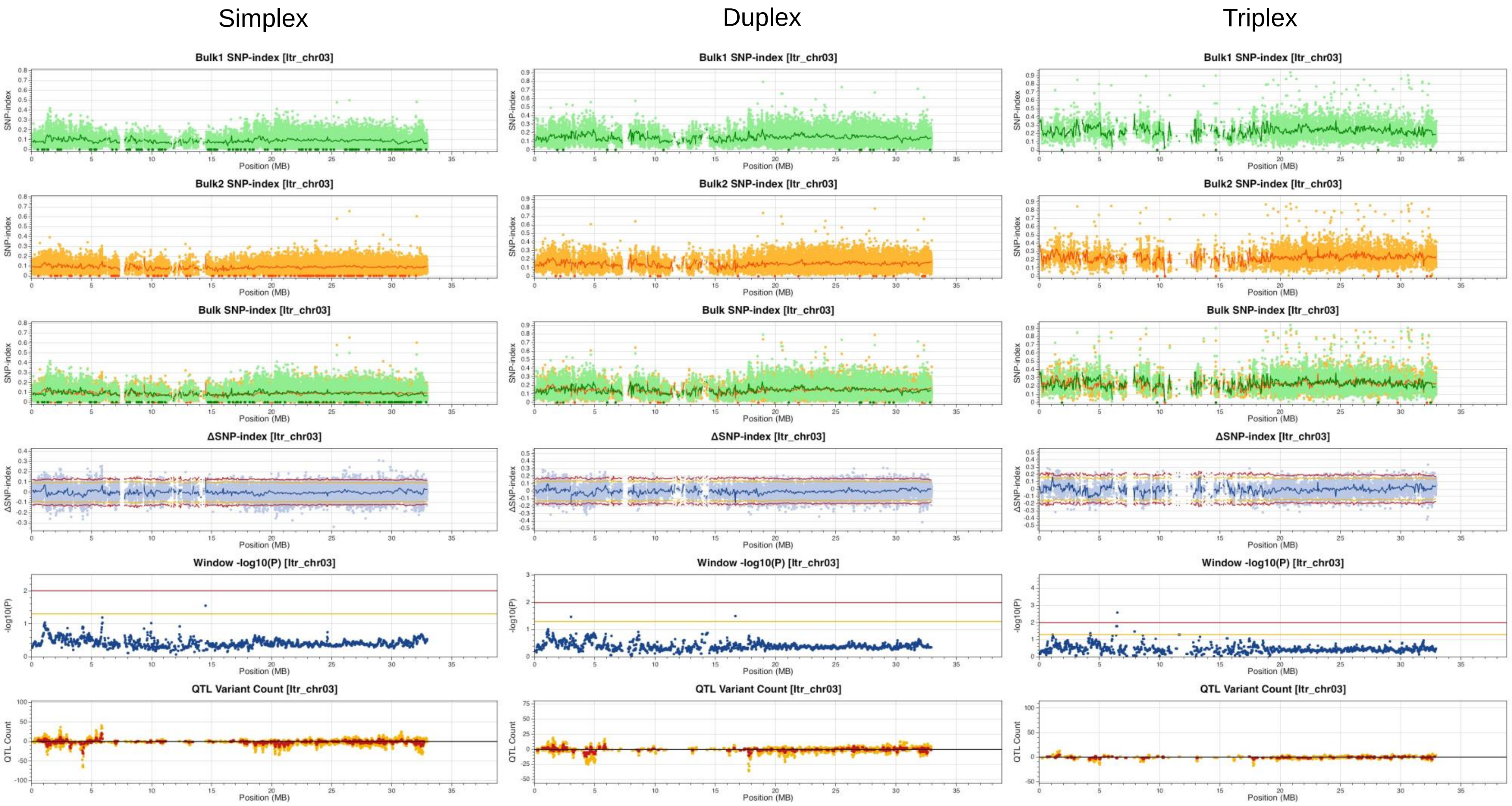

Supplemental Fig. 1. (continued)

B BK-derived variants

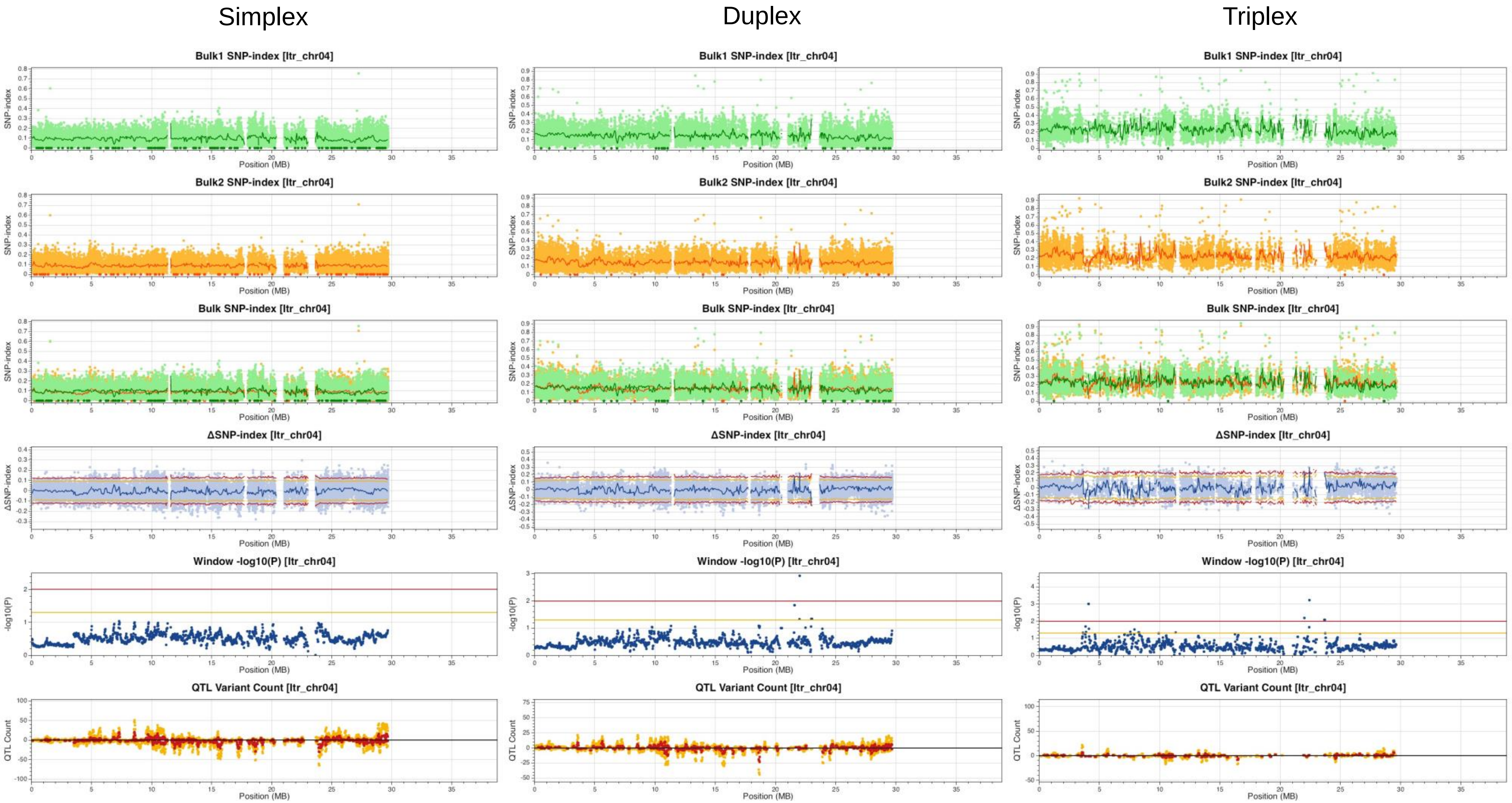

Supplemental Fig. 1. (continued)

B BK-derived variants

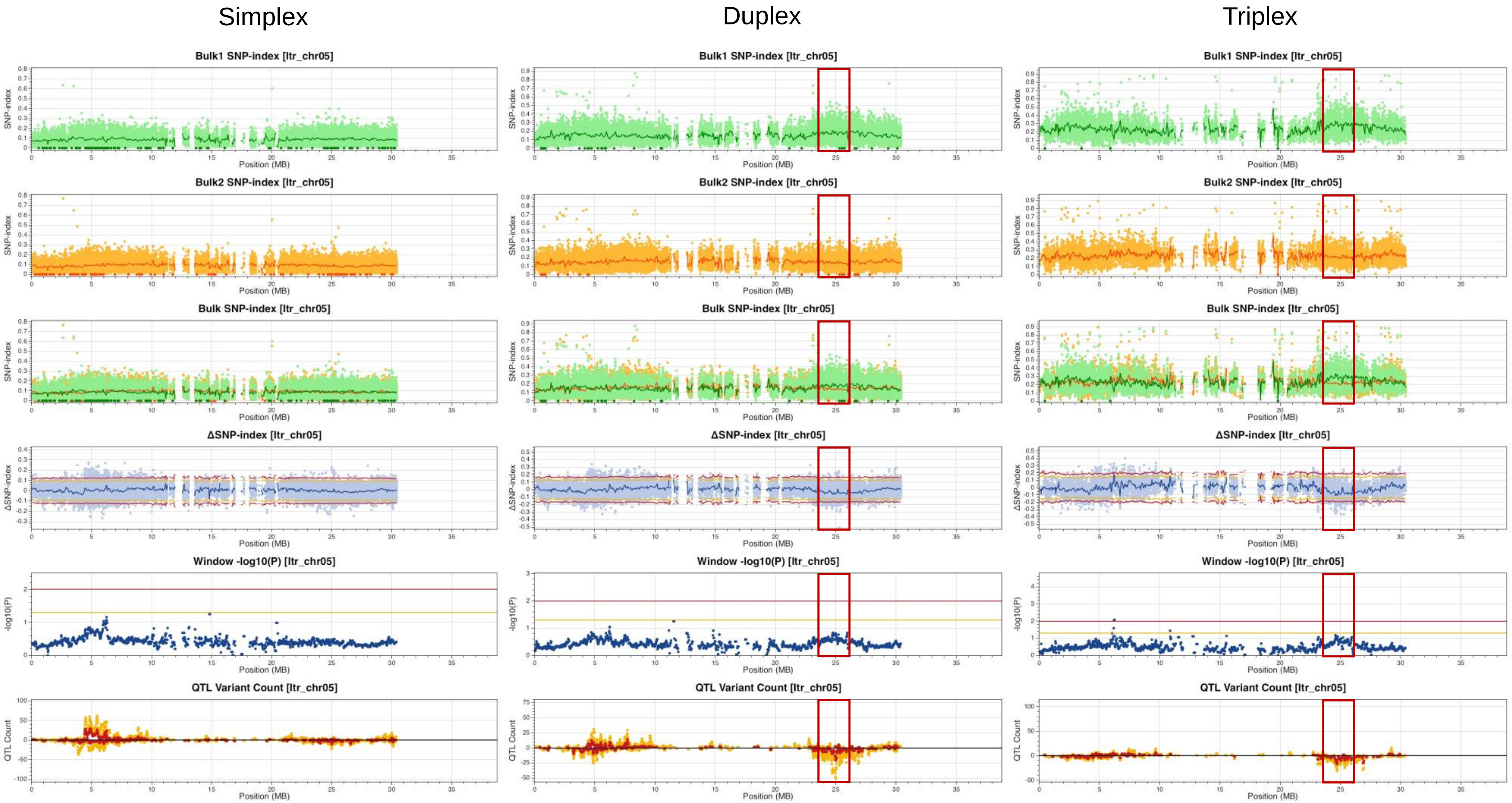

Supplemental Fig. 1. (continued)

B BK-derived variants

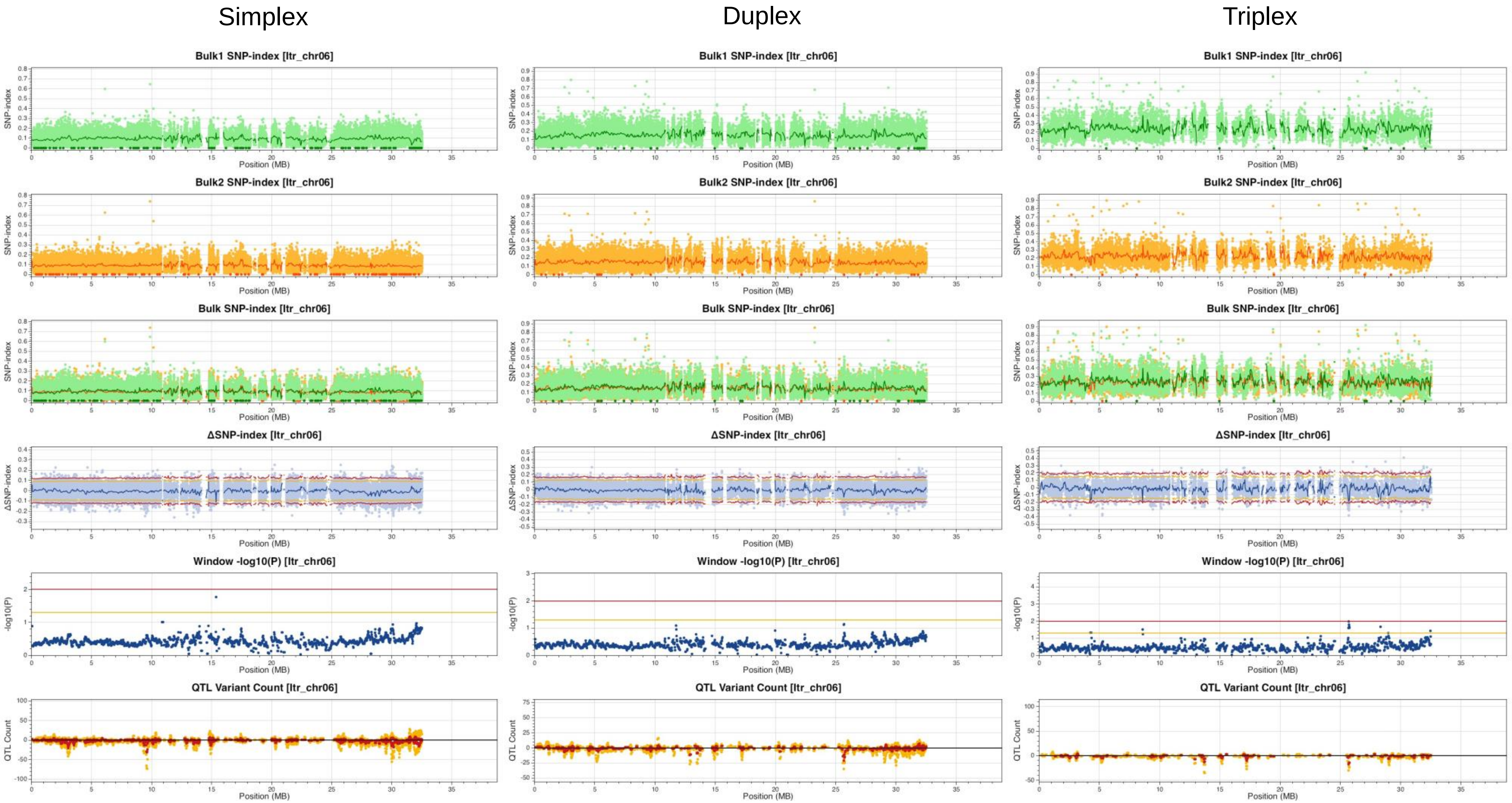

Supplemental Fig. 1. (continued)

B BK-derived variants

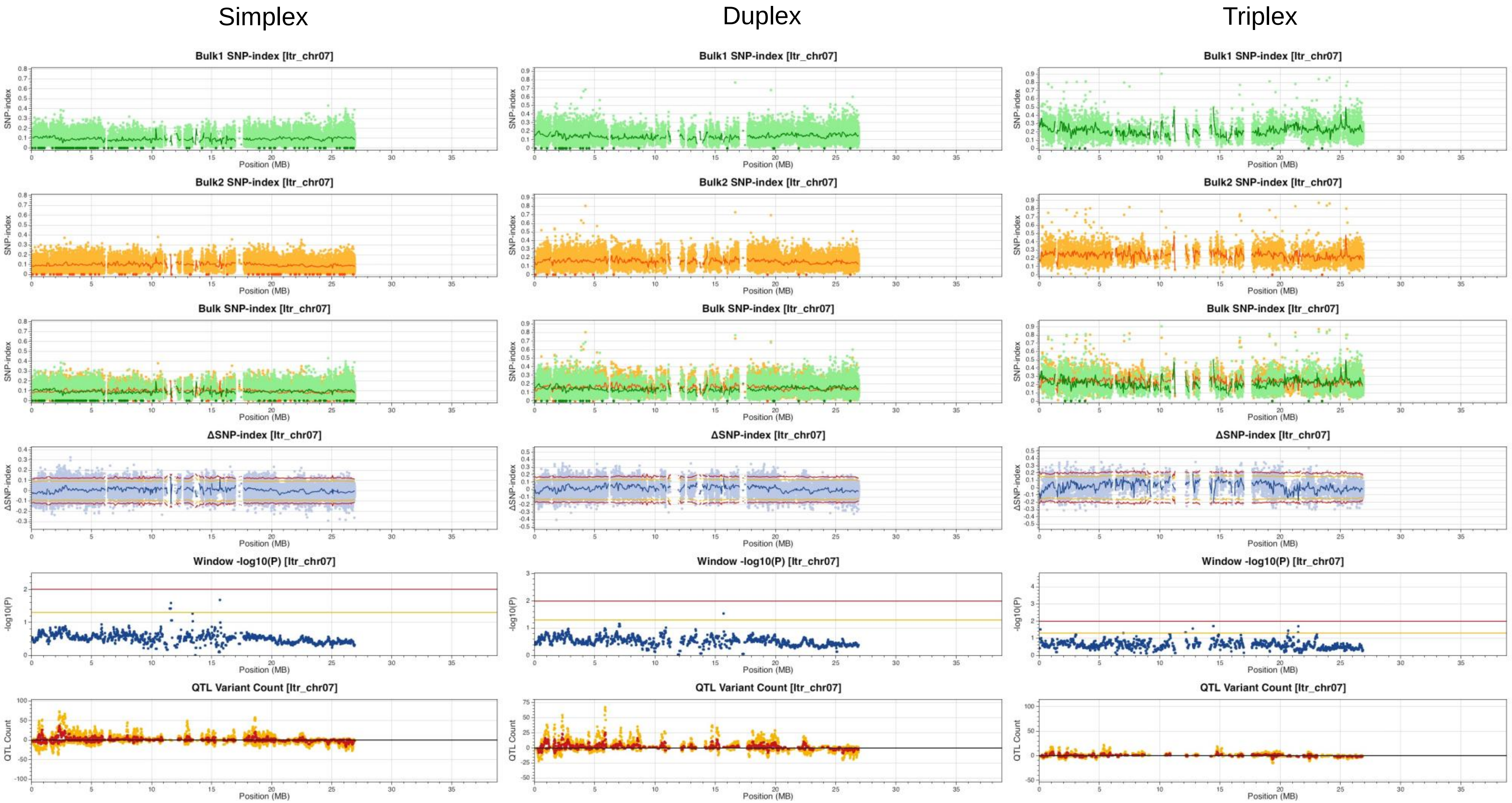

Supplemental Fig. 1. (continued)

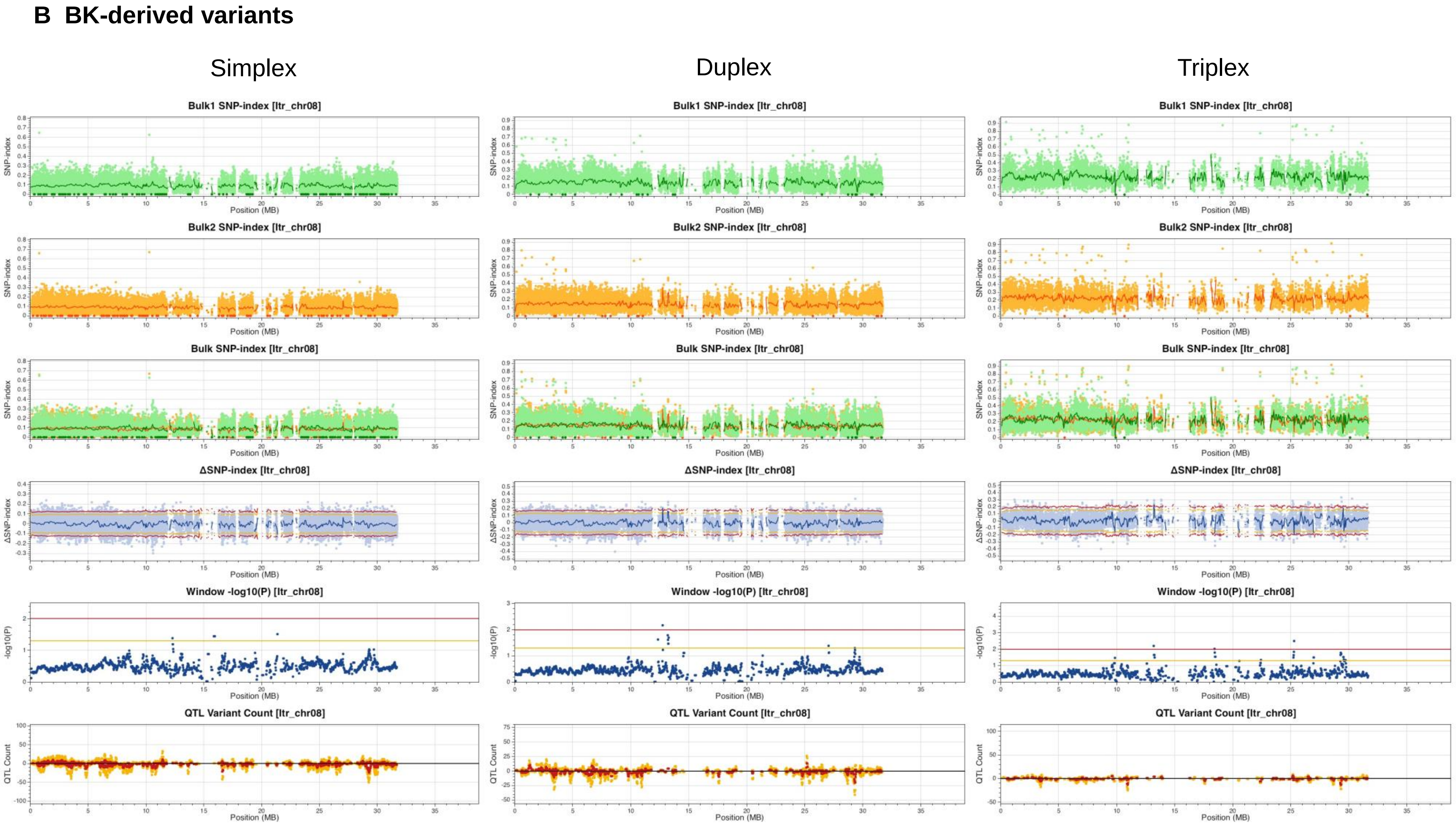

Supplemental Fig. 1. (continued)

B BK-derived variants

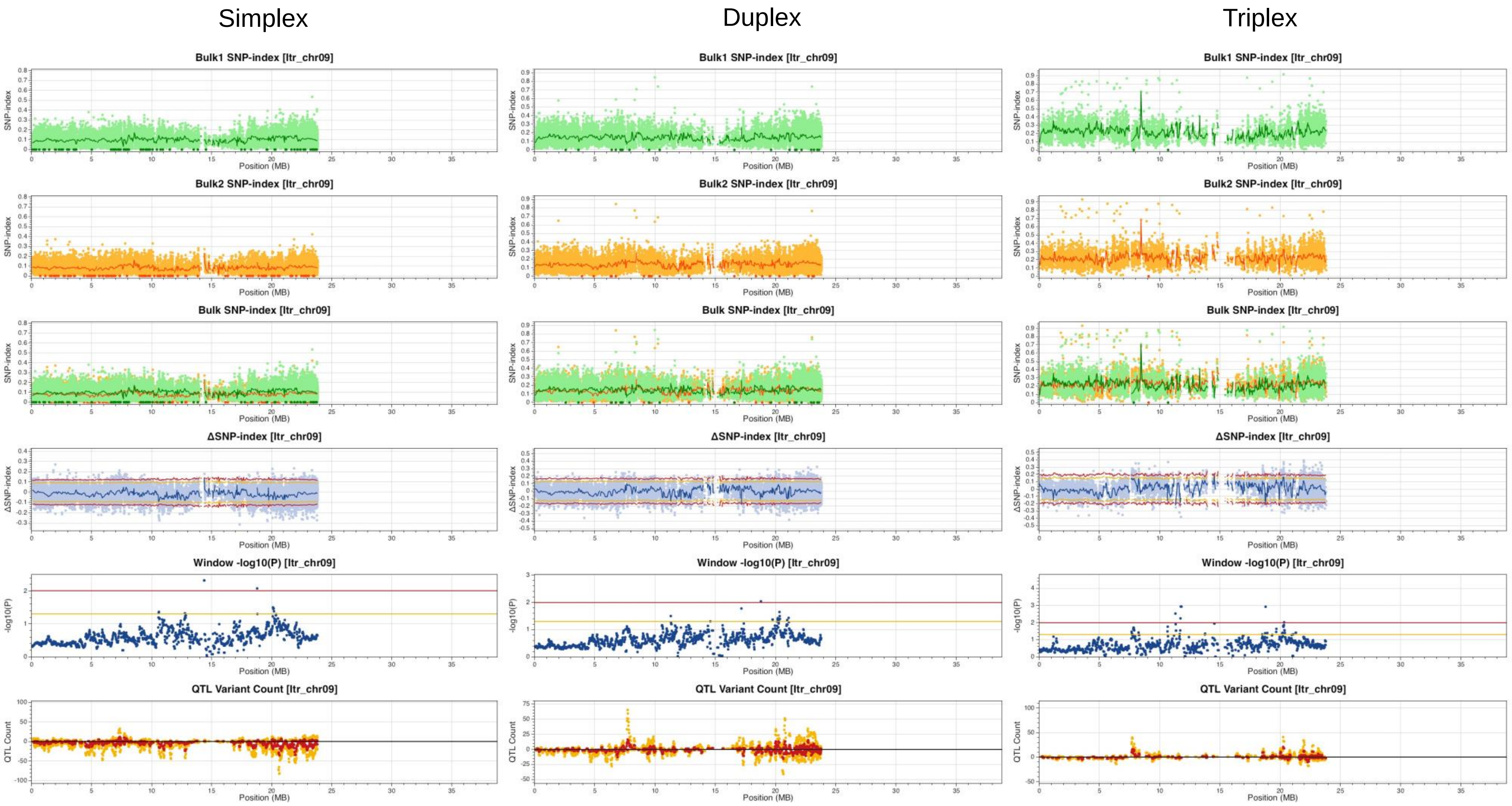

Supplemental Fig. 1. (continued)

B BK-derived variants

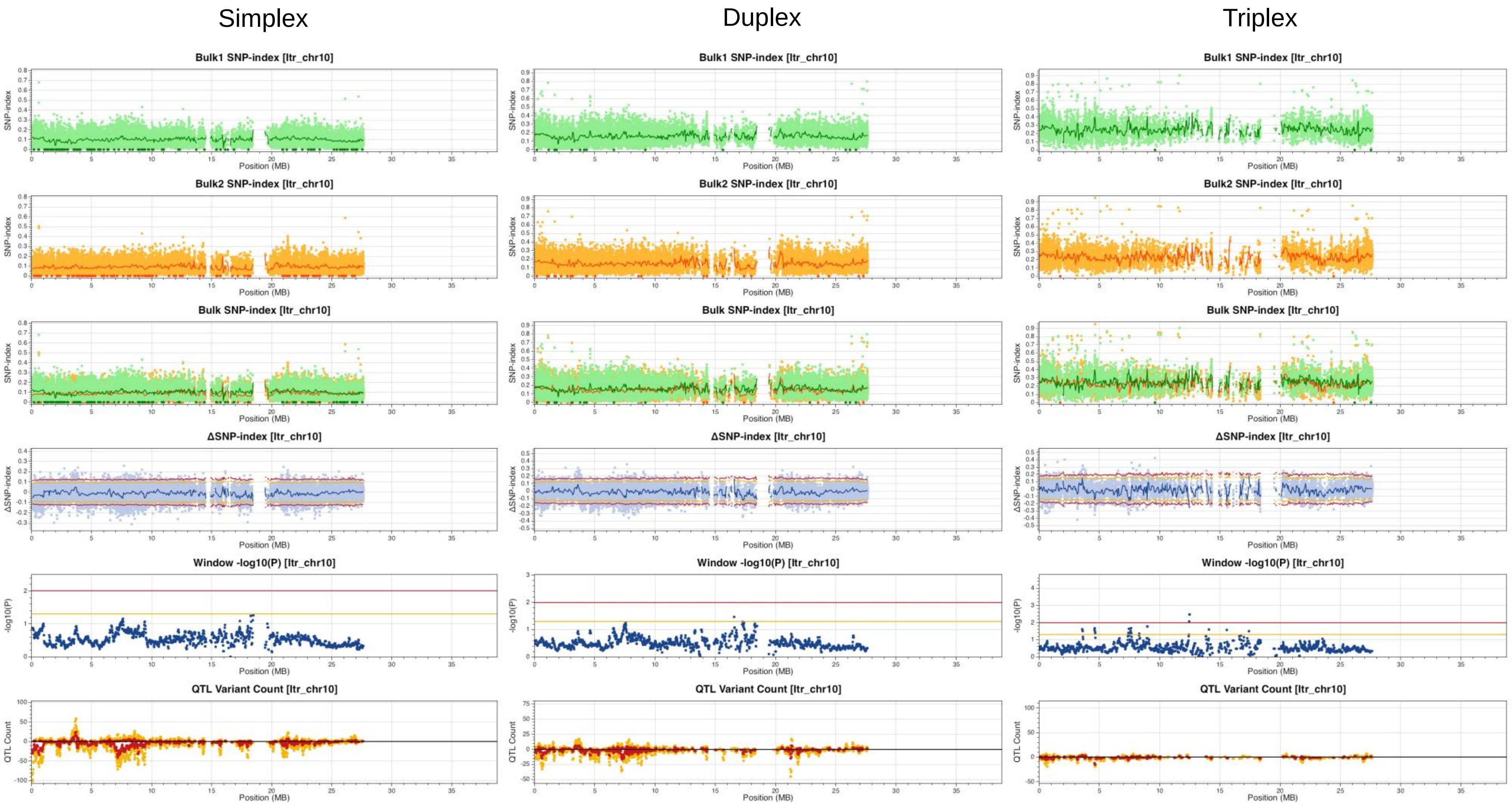

Supplemental Fig. 1. (continued)

B BK-derived variants

Simplex Duplex Triplex

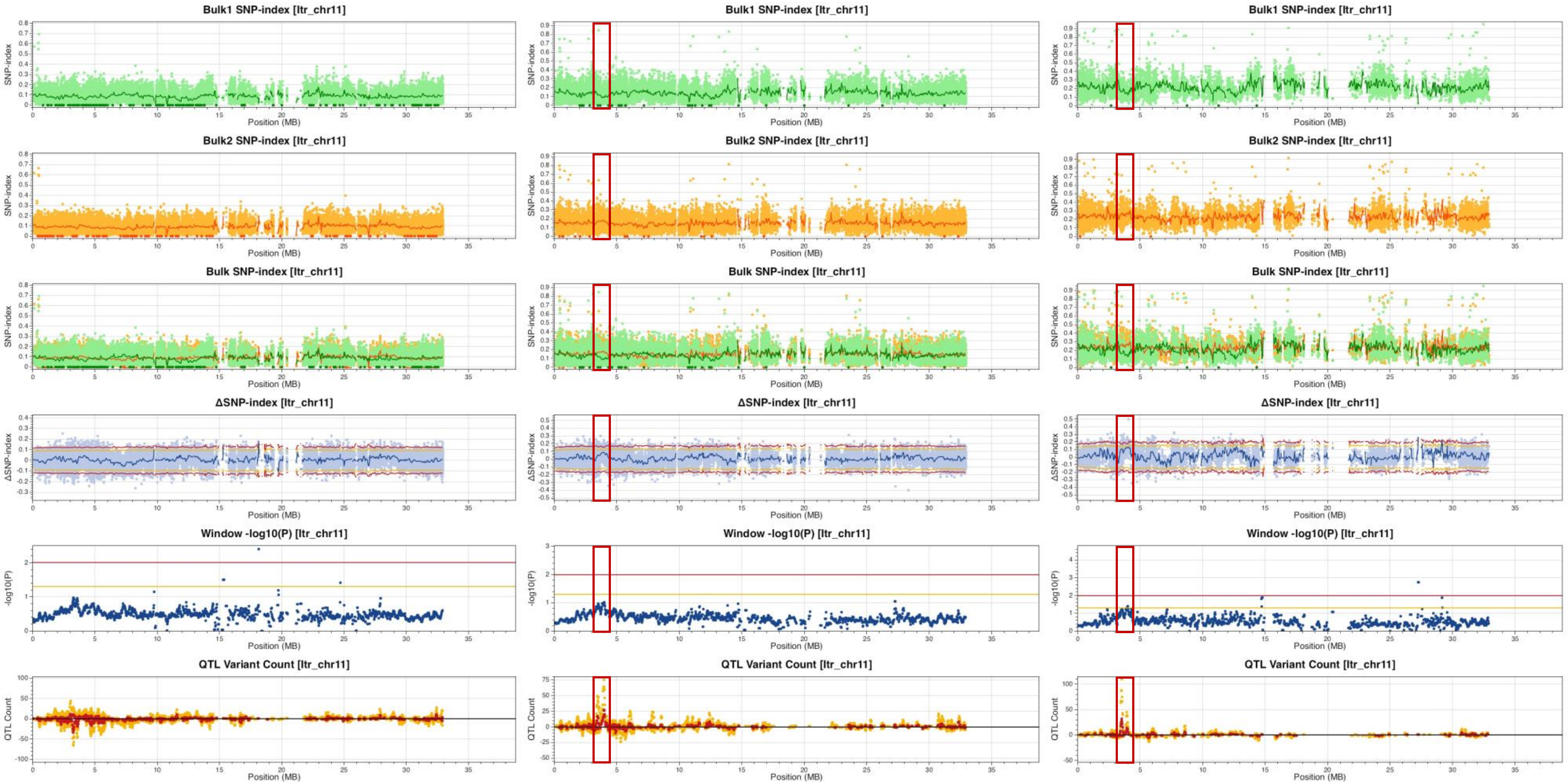

Supplemental Fig. 1. (continued)

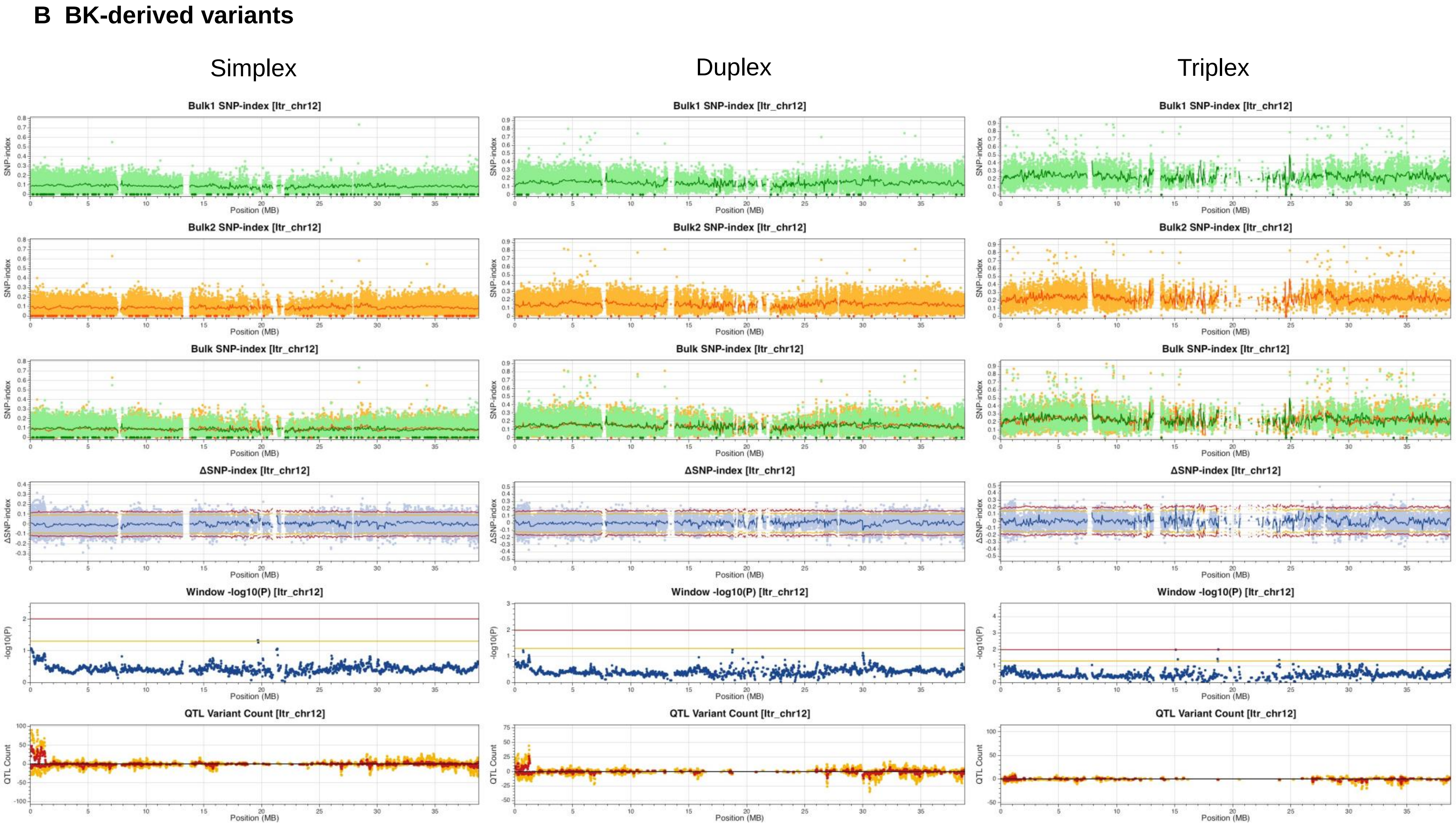

Supplemental Fig. 1. (continued)

B BK-derived variants

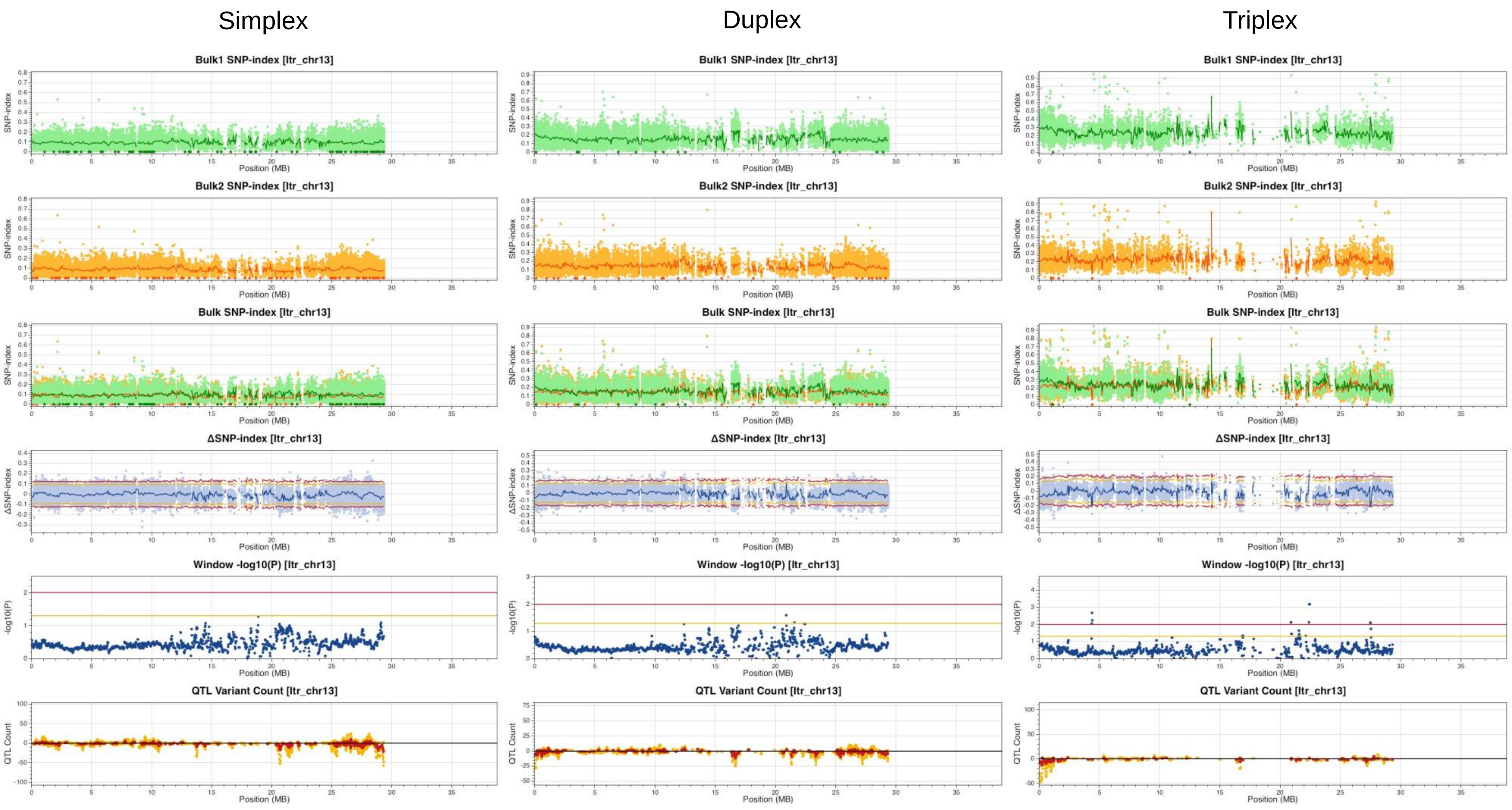

Supplemental Fig. 1. (continued)

B BK-derived variants

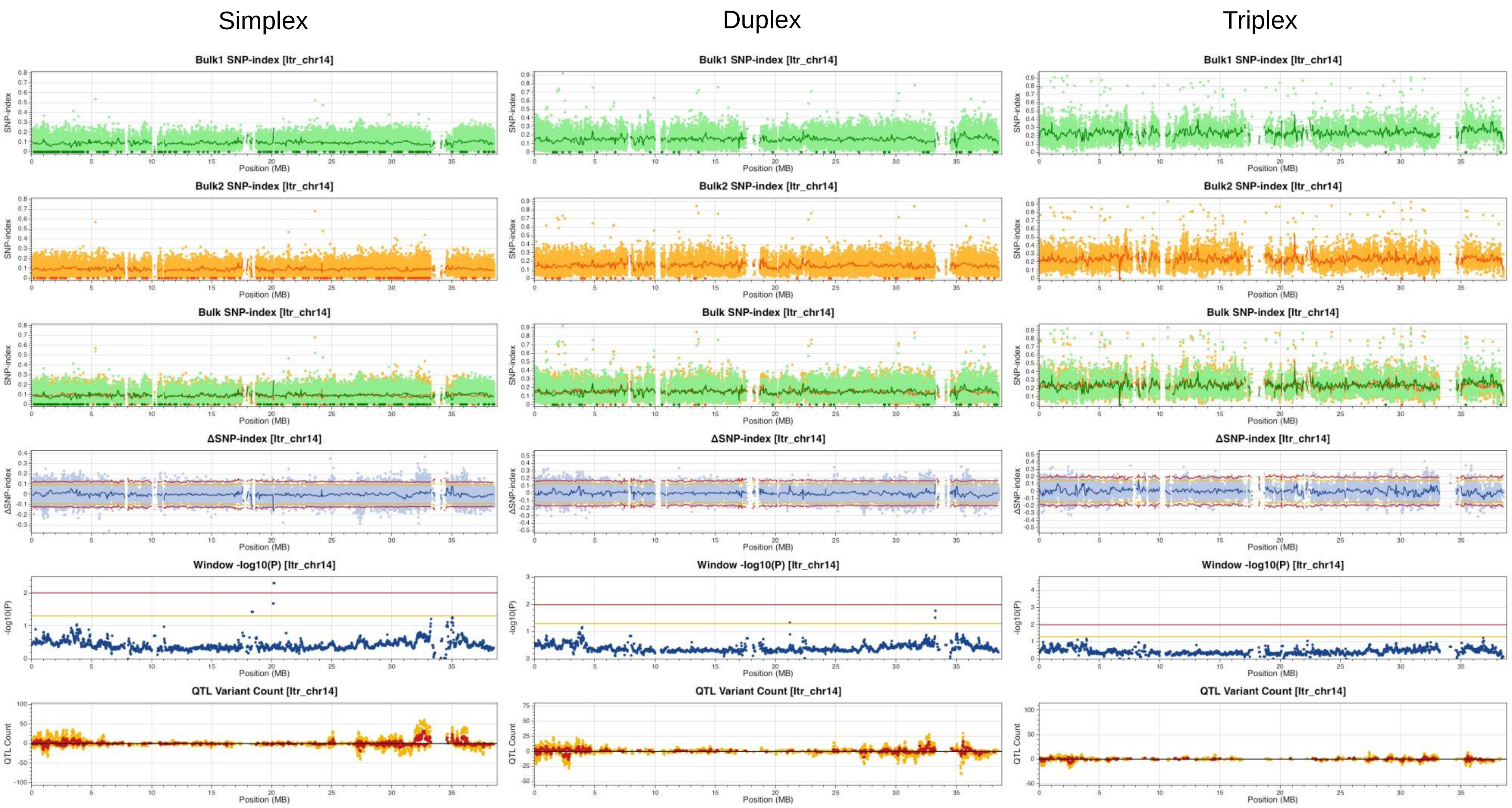

Supplemental Fig. 1. (continued)

B BK-derived variants

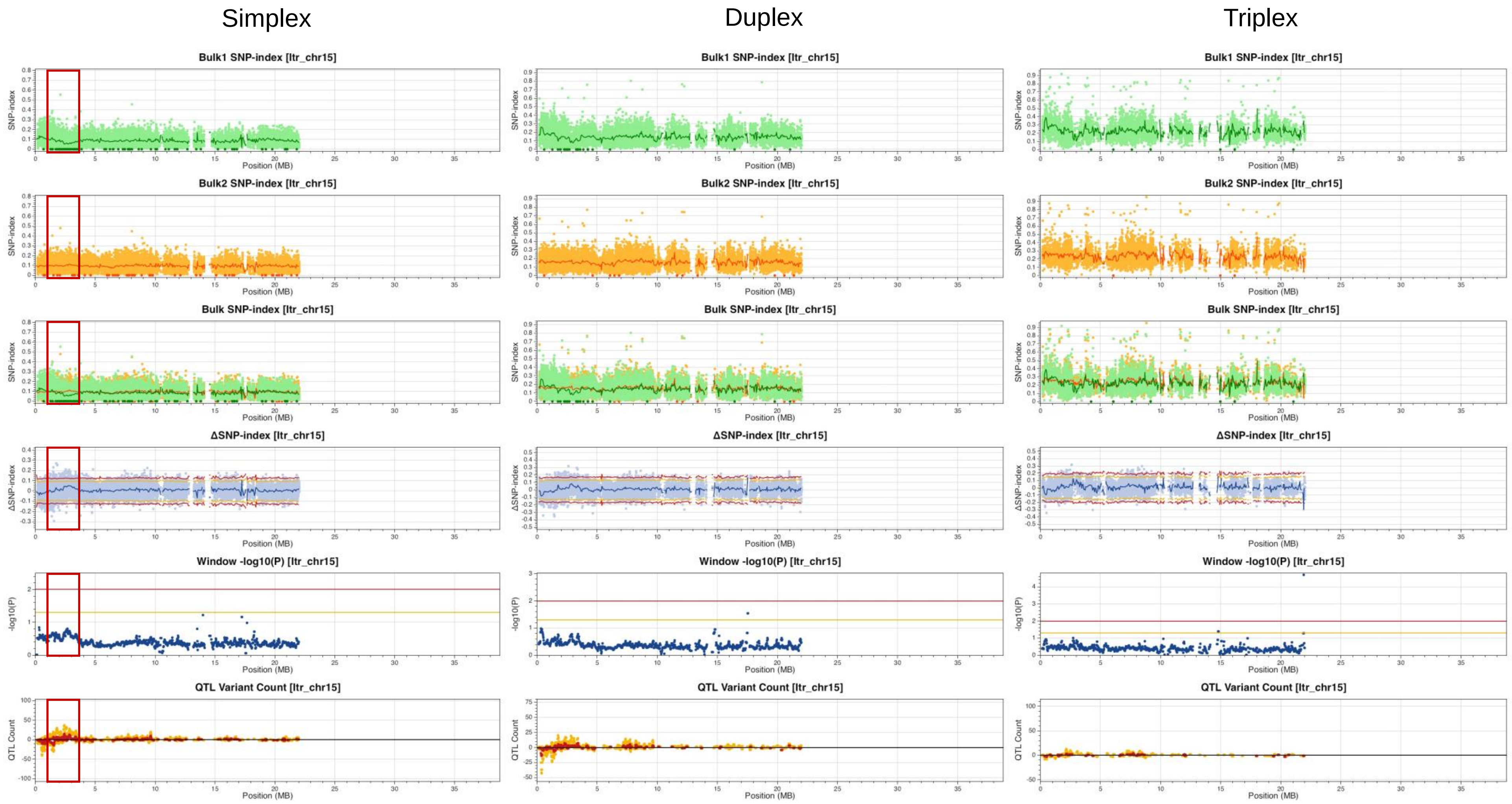

Supplemental Fig. 1. (continued)

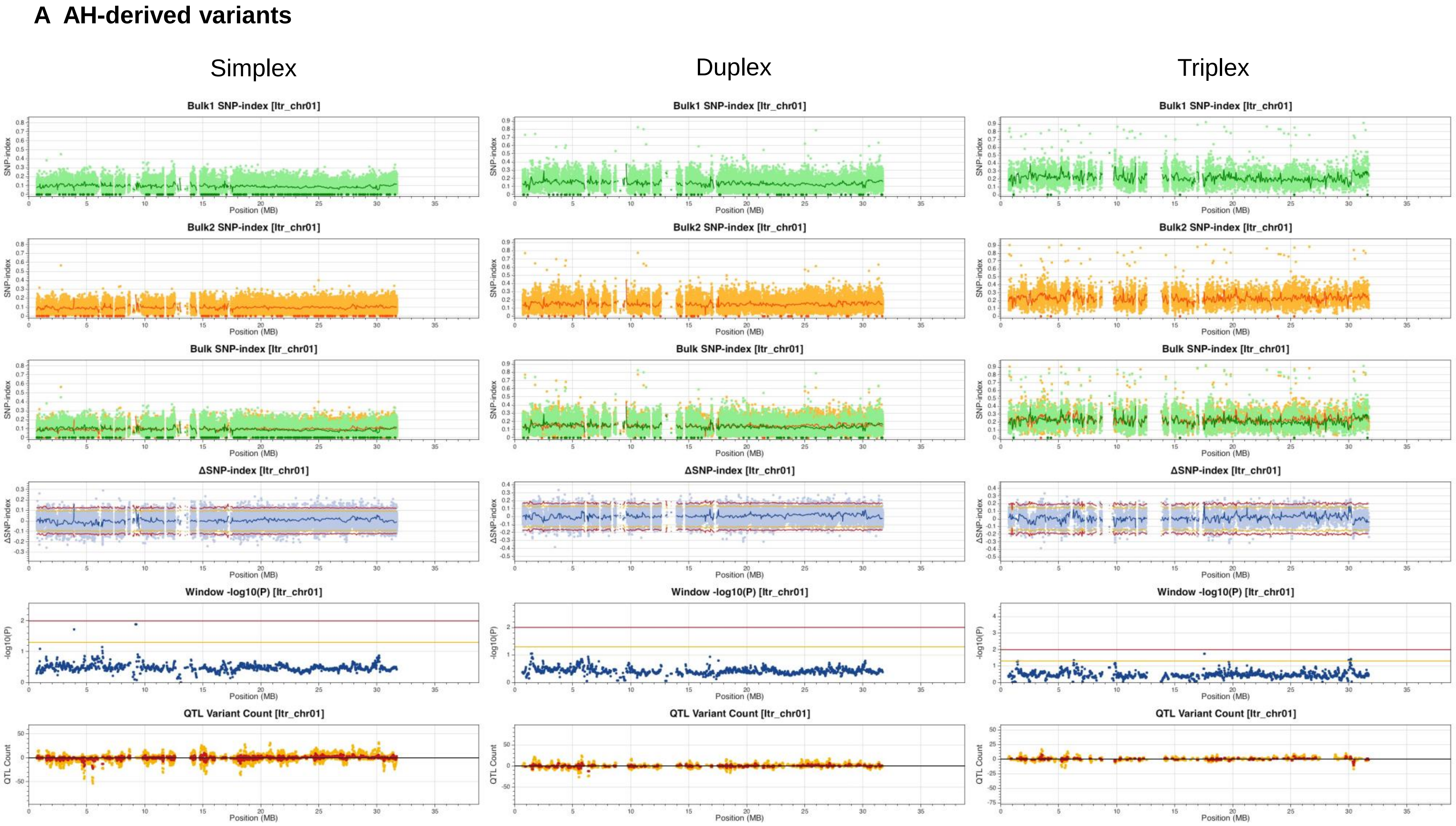

Supplemental Fig. 2. Genomic regions regulating starch GT in ABF1 progenies. (A) Polyploid QTL-seq analysis using each of AH-derived simplex, duplex and triplex variants. (B) Polyploid QTL-seq analysis using each of BK-derived simplex, duplex and triplex variants. SNP-index plots of low-temperature (LT) bulk and high-temperature (HT) bulk, their superimposed plot, ΔSNP-index plot, window -$\log_{10}P$ plot, and QTL variant count plot are depicted similarly to Fig. 6. Red frames indicate candidate regions for QTLs.

A AH-derived variants

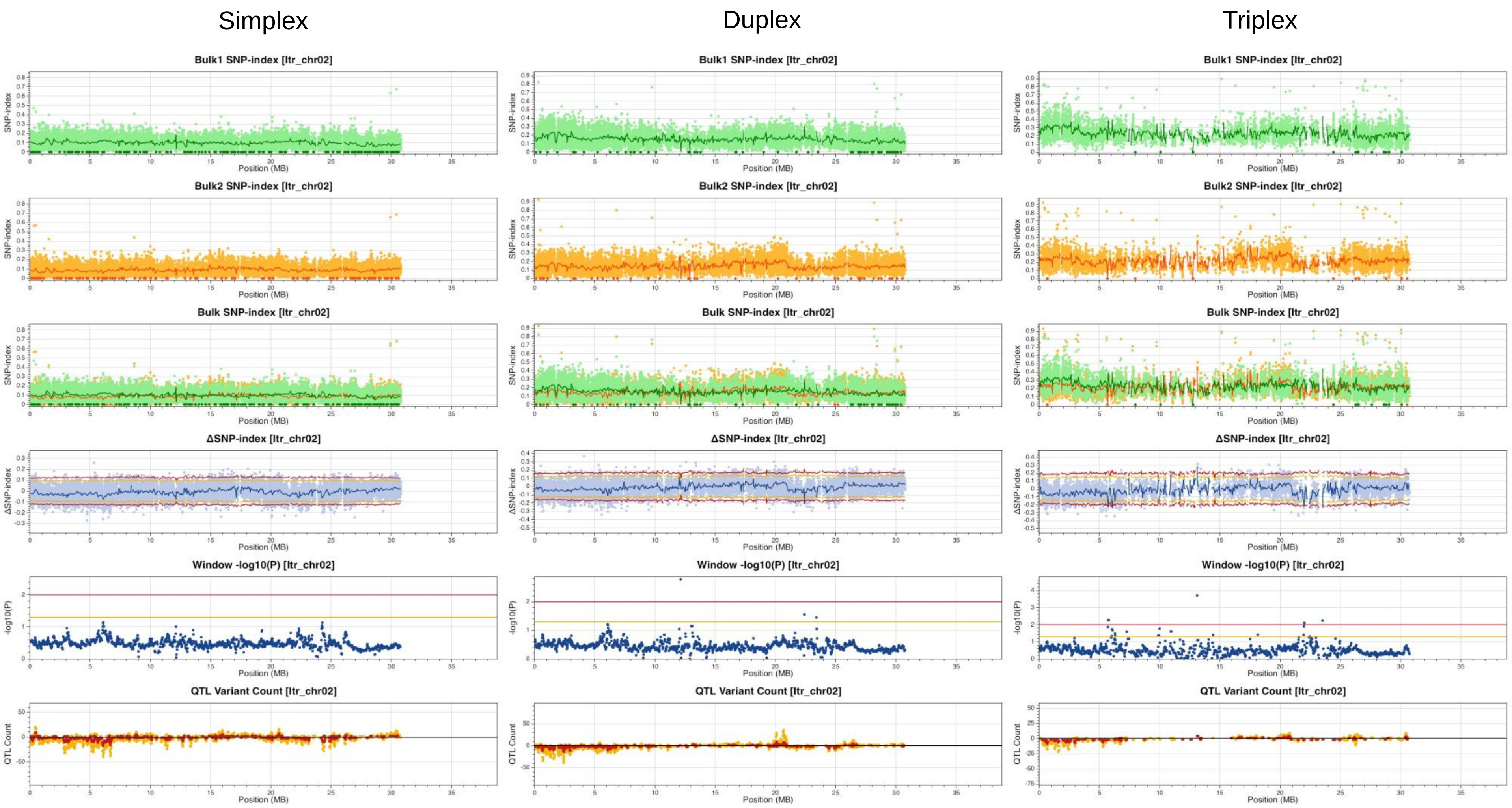

Supplemental Fig. 2. (continued)

A AH-derived variants

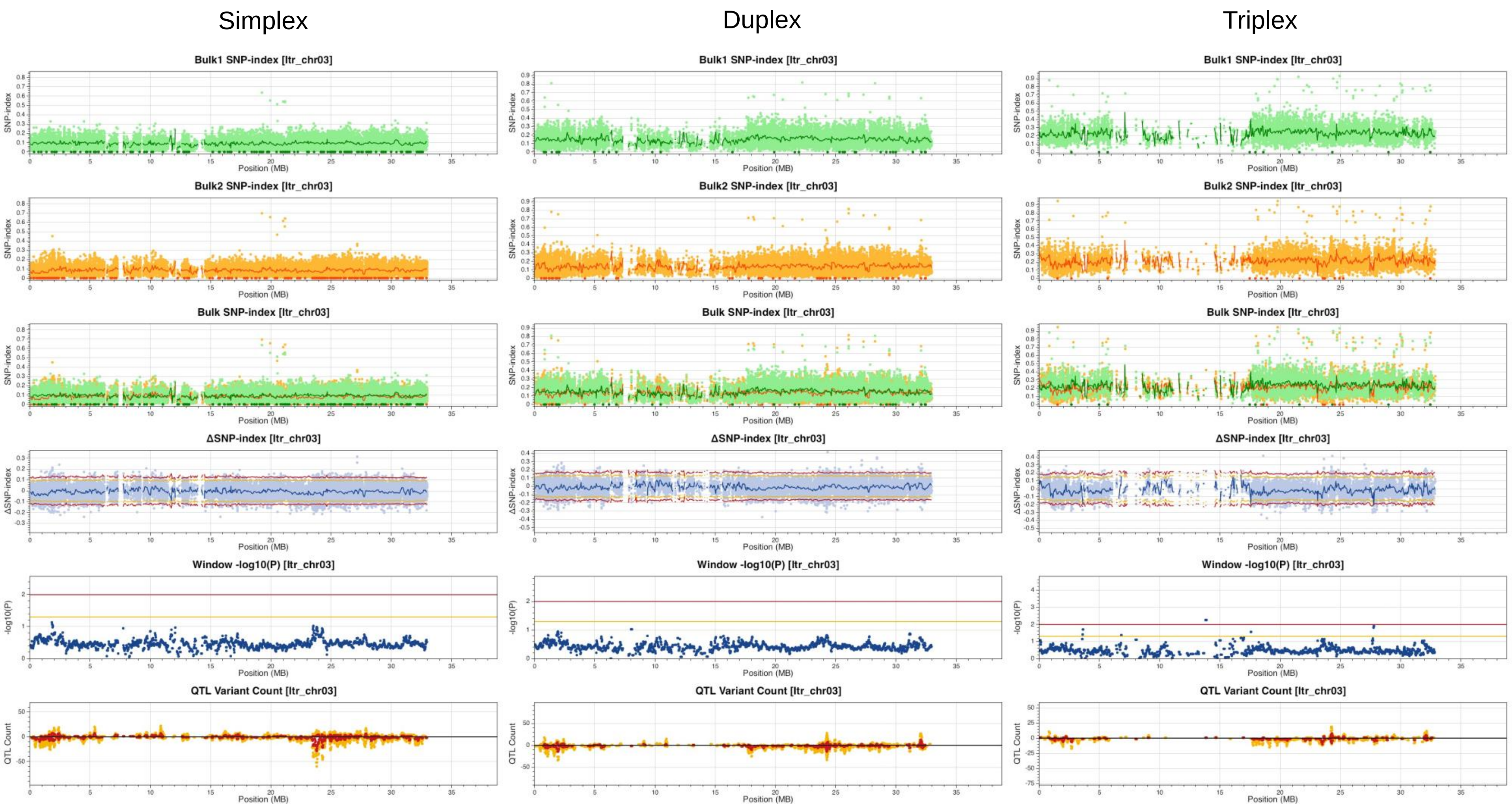

Supplemental Fig. 2. (continued)

A AH-derived variants

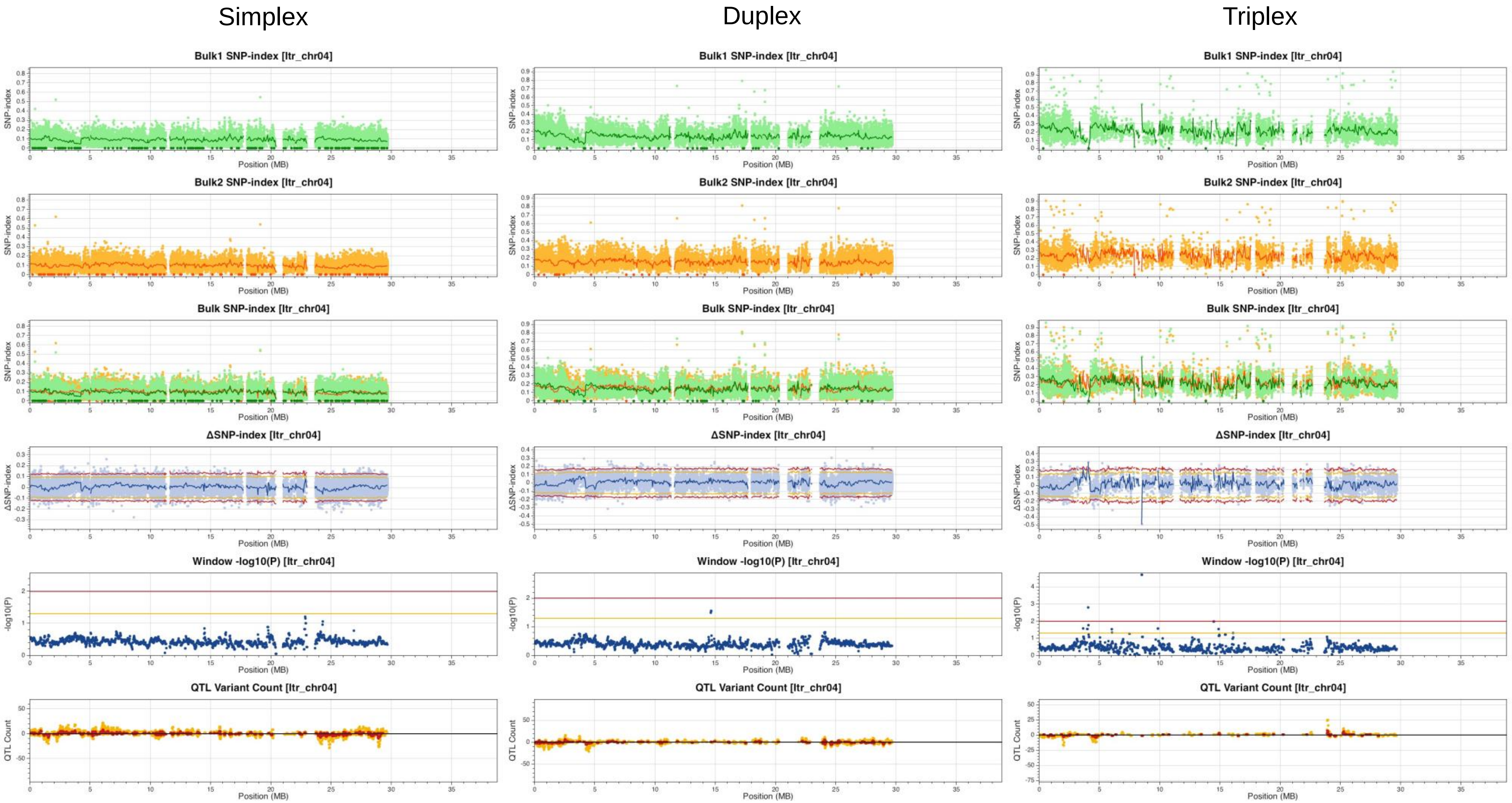

Supplemental Fig. 2. (continued)

A AH-derived variants

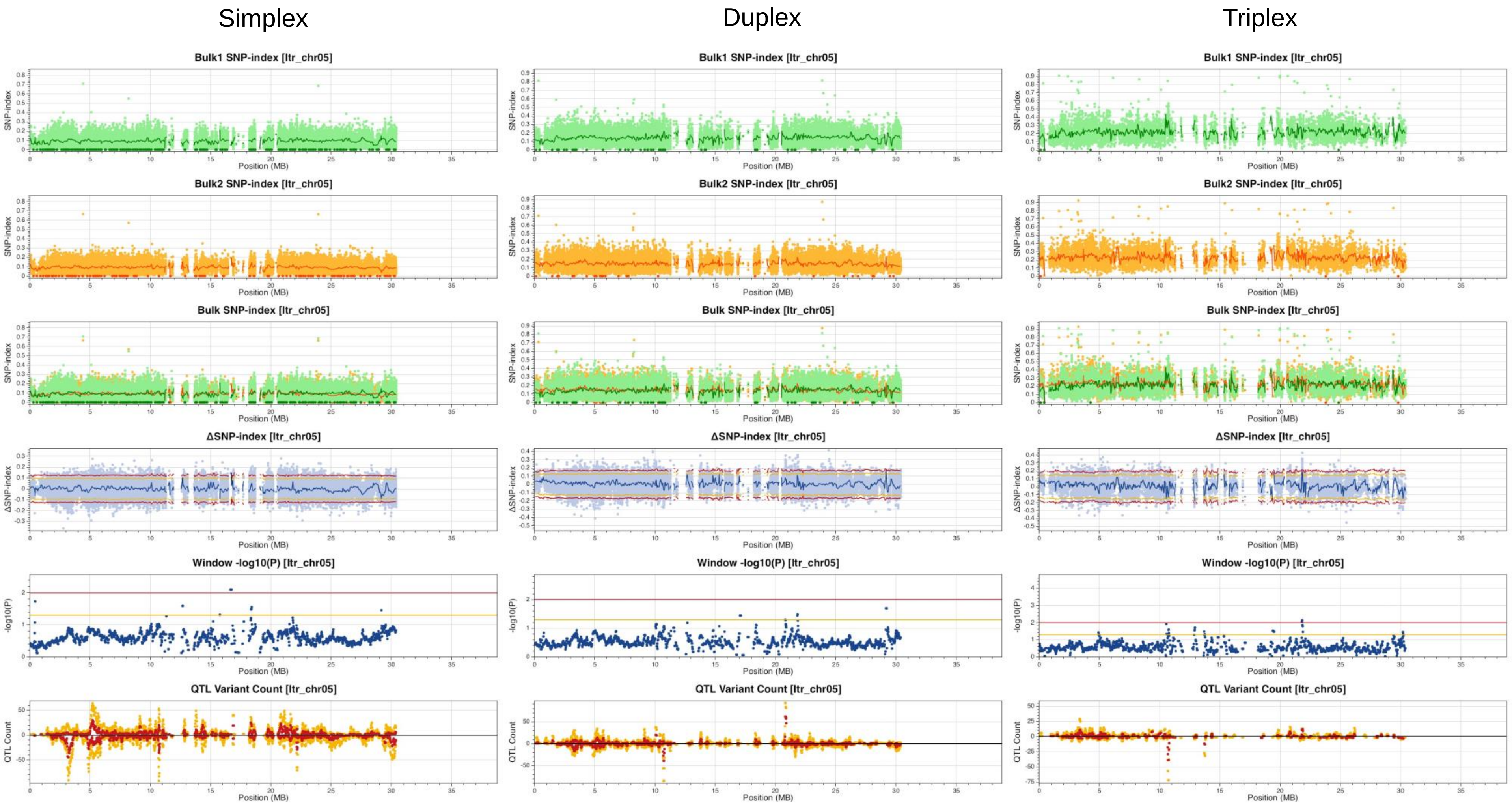

Supplemental Fig. 2. (continued)

A AH-derived variants

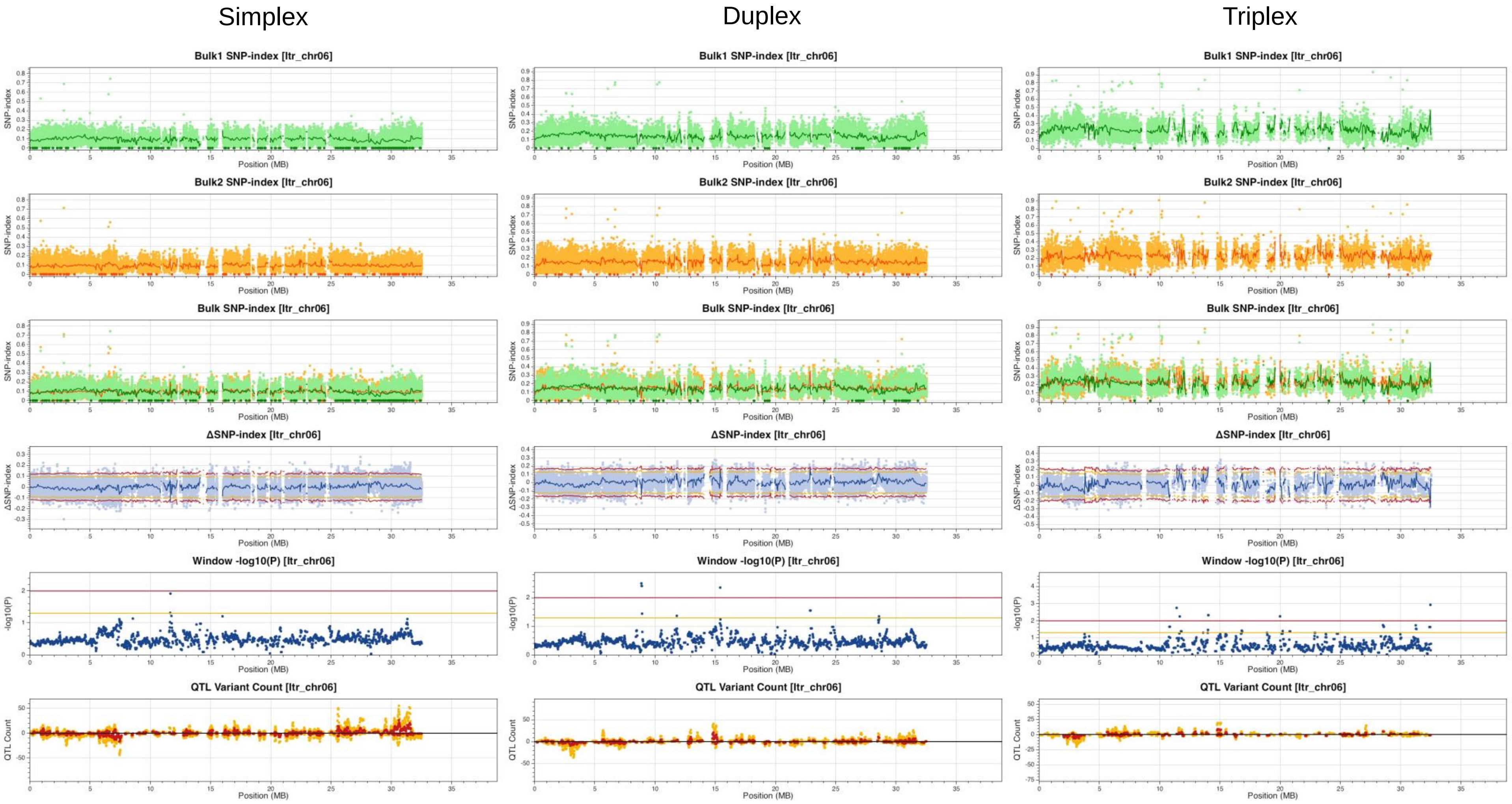

Supplemental Fig. 2. (continued)

A AH-derived variants

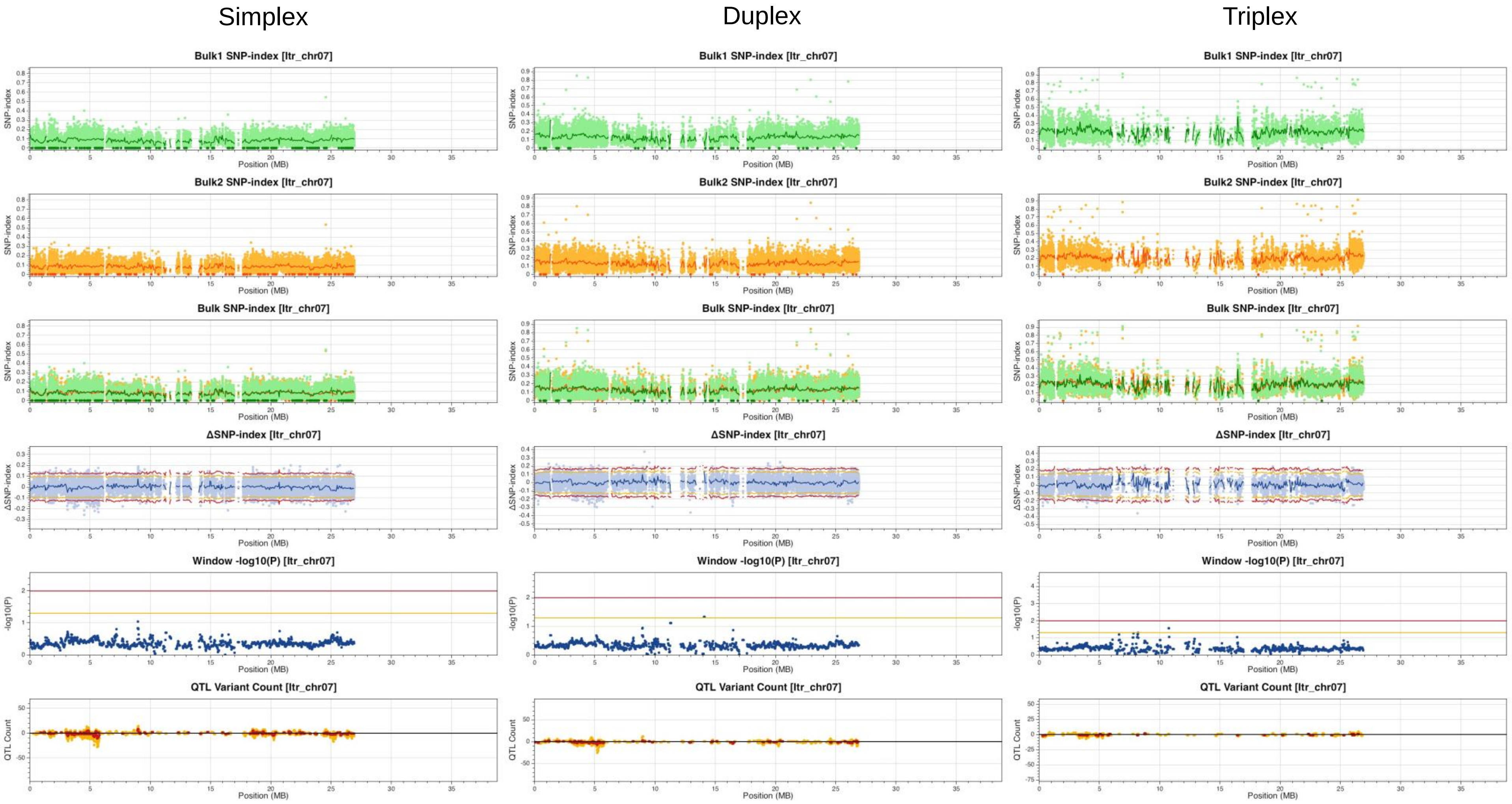

Supplemental Fig. 2. (continued)

A AH-derived variants

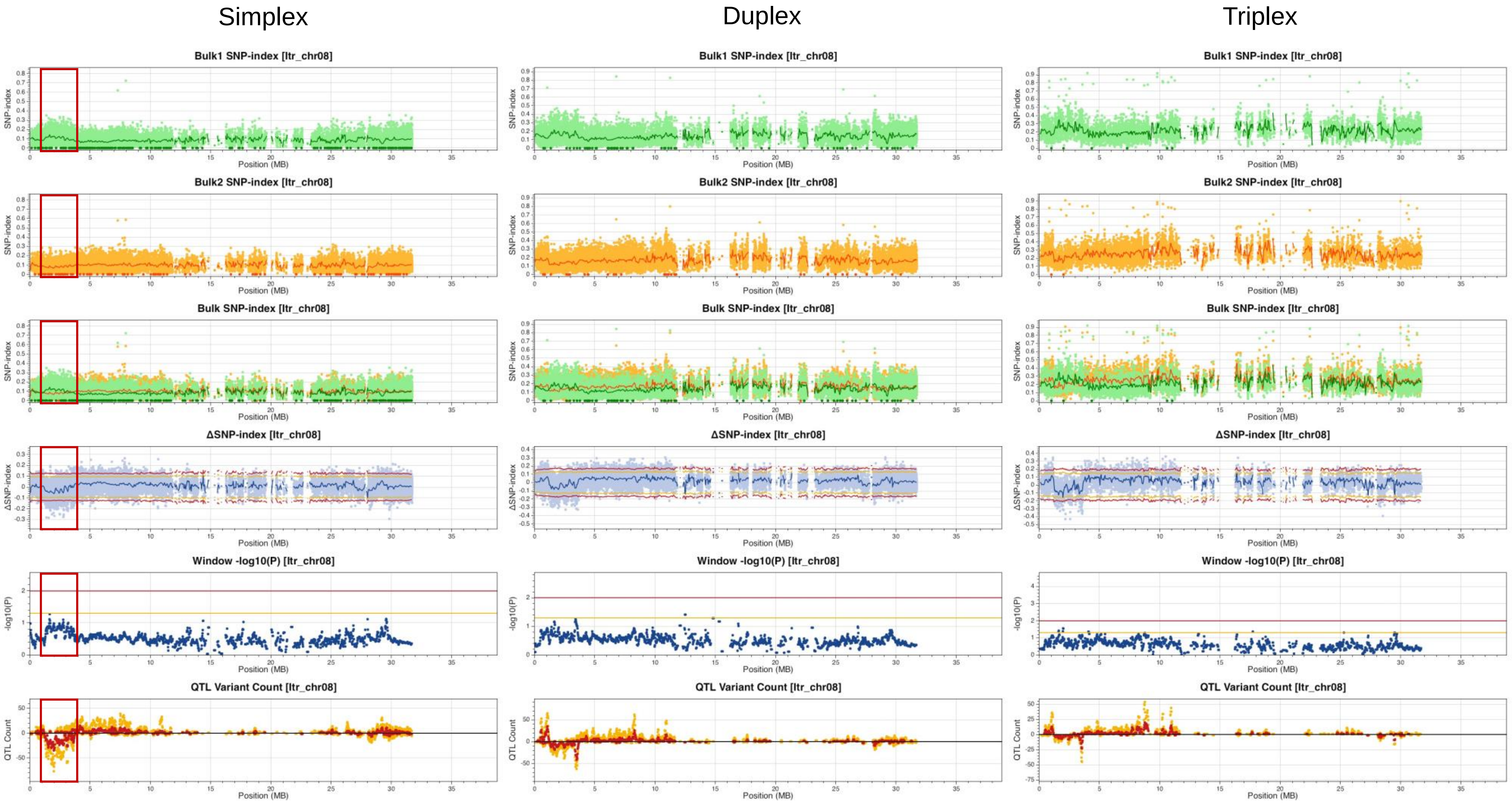

Supplemental Fig. 2. (continued)

A AH-derived variants

Simplex | Duplex | Triplex

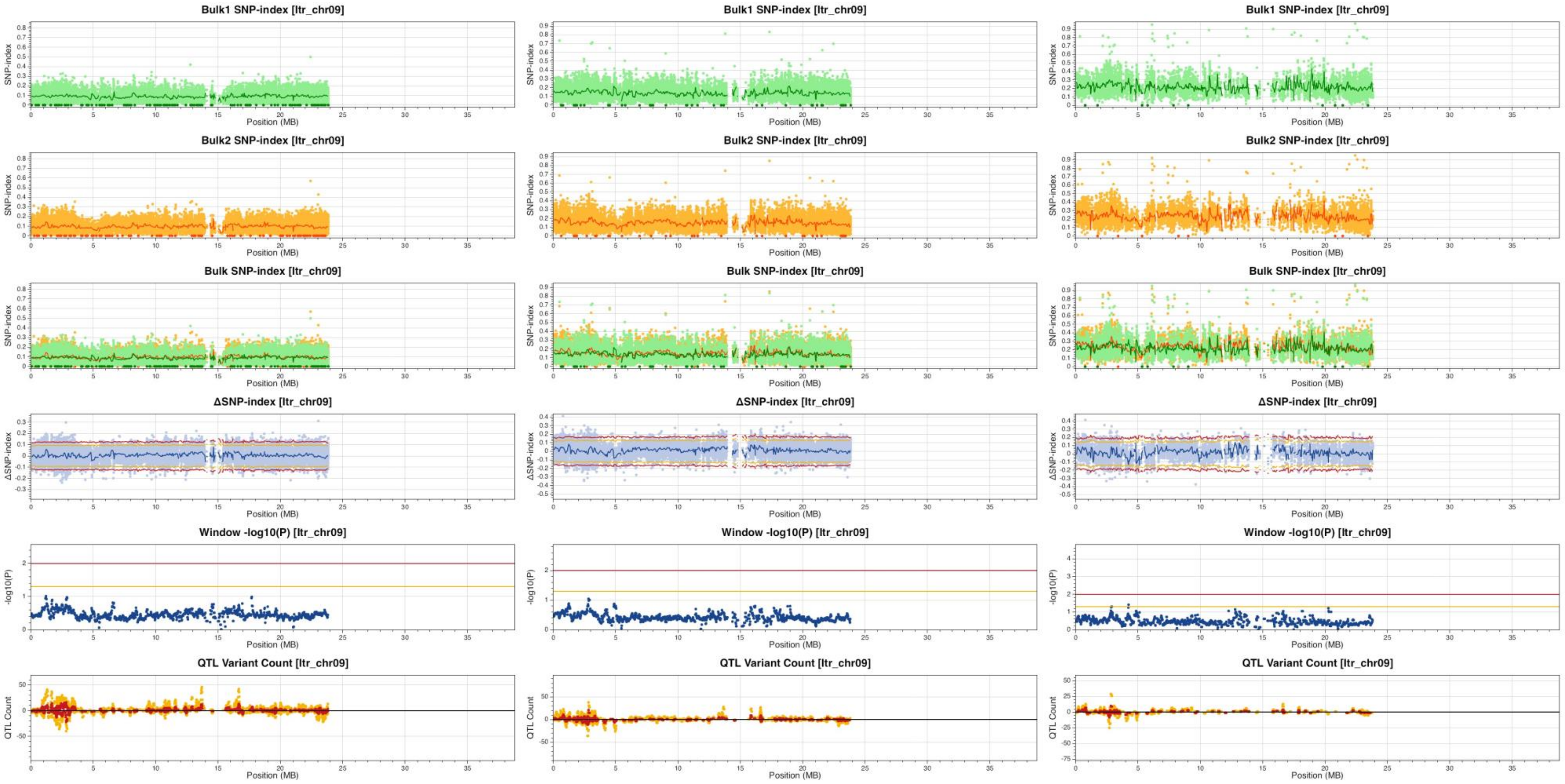

Supplemental Fig. 2. (continued)

A AH-derived variants

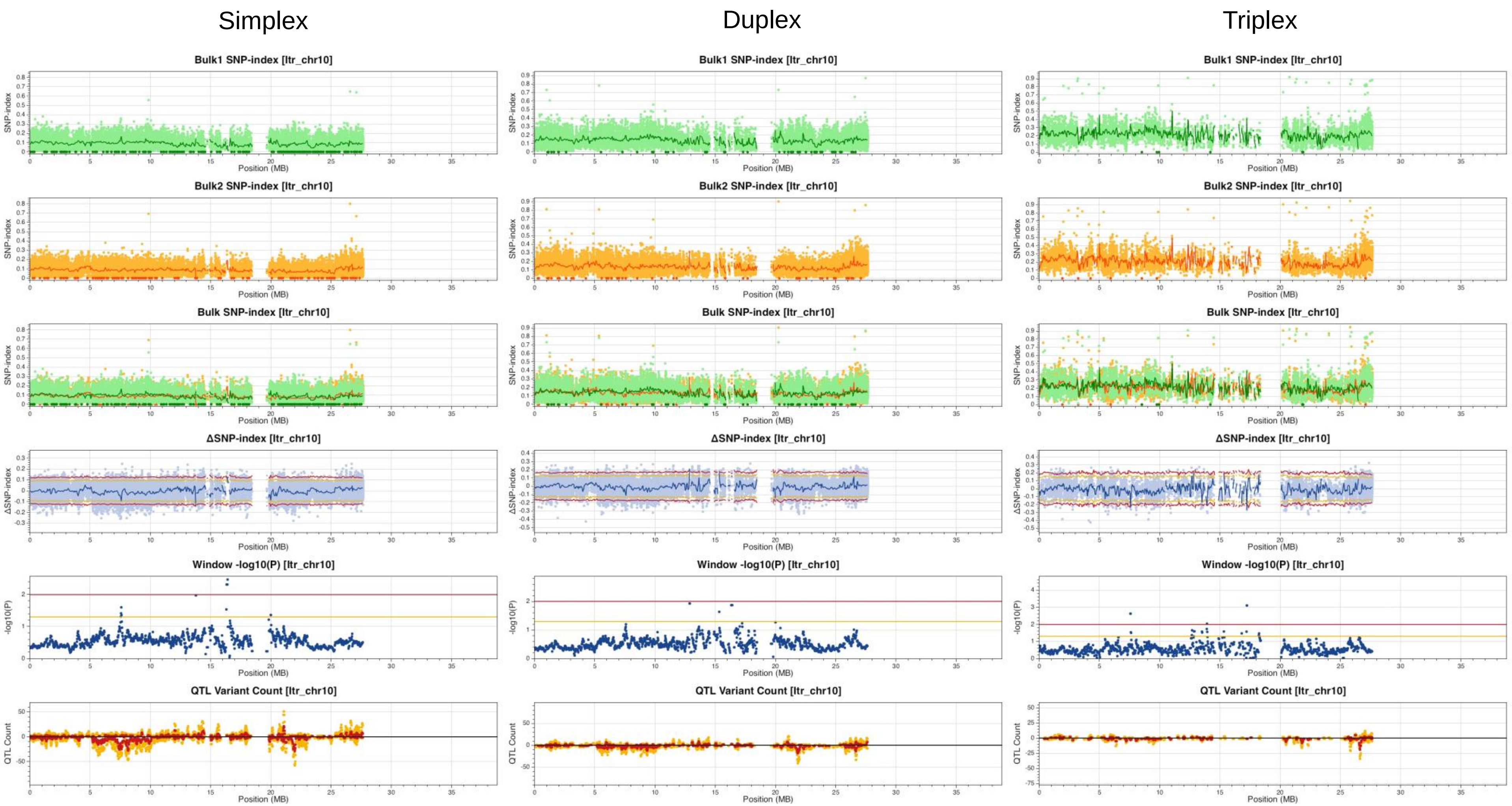

Supplemental Fig. 2. (continued)

A AH-derived variants

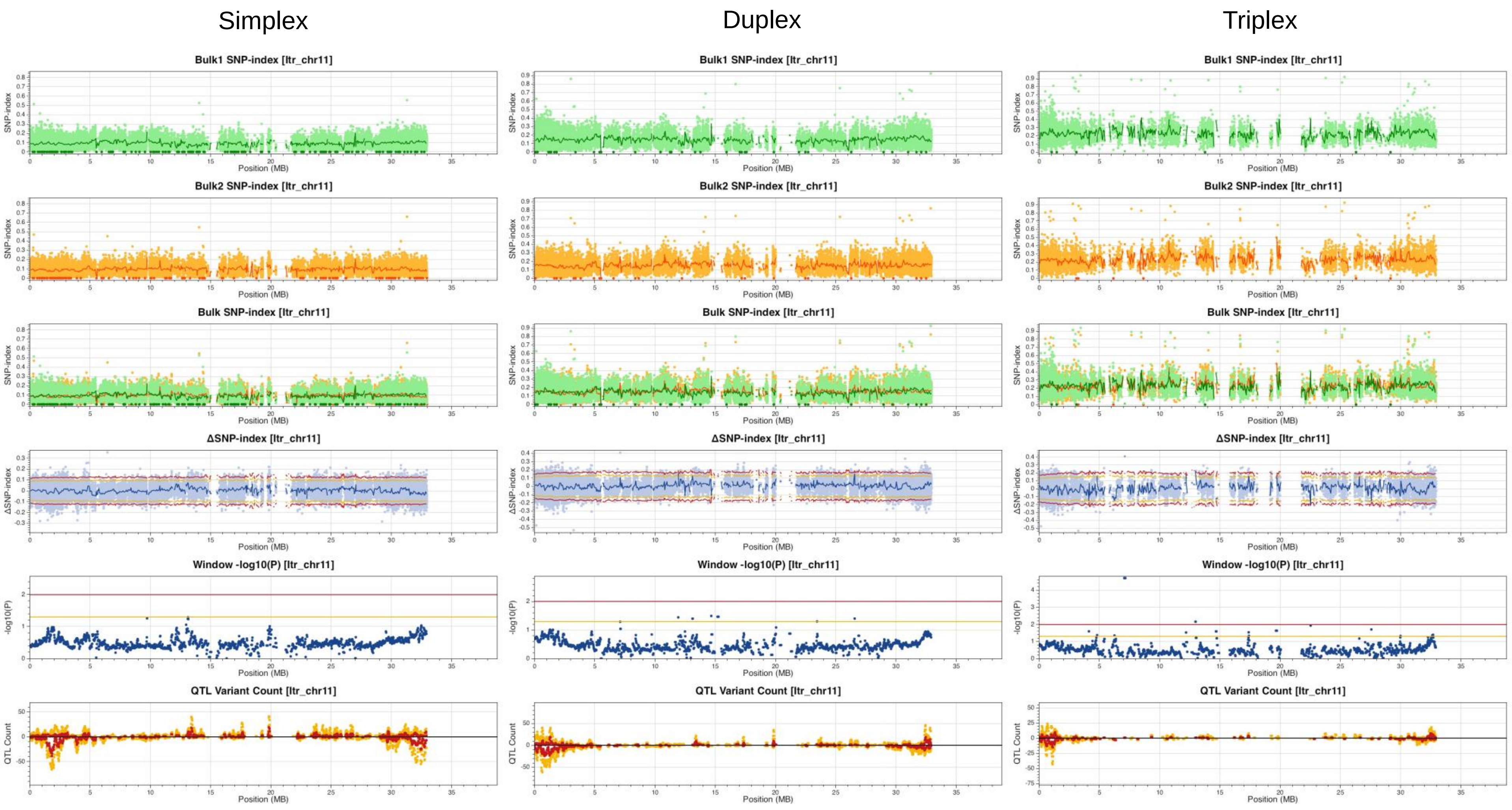

Supplemental Fig. 2. (continued)

A AH-derived variants

Simplex Duplex Triplex

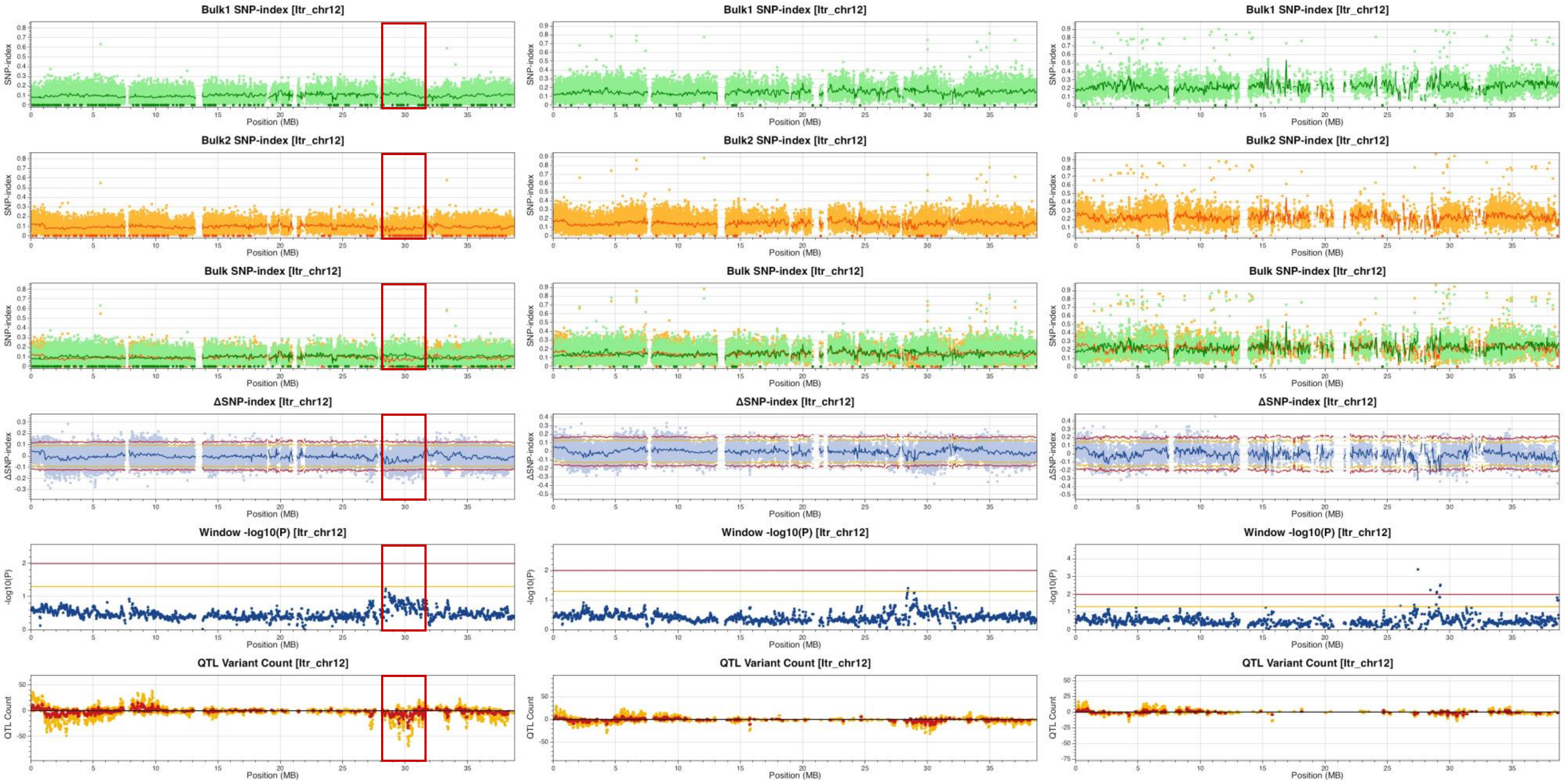

Supplemental Fig. 2. (continued)

A AH-derived variants

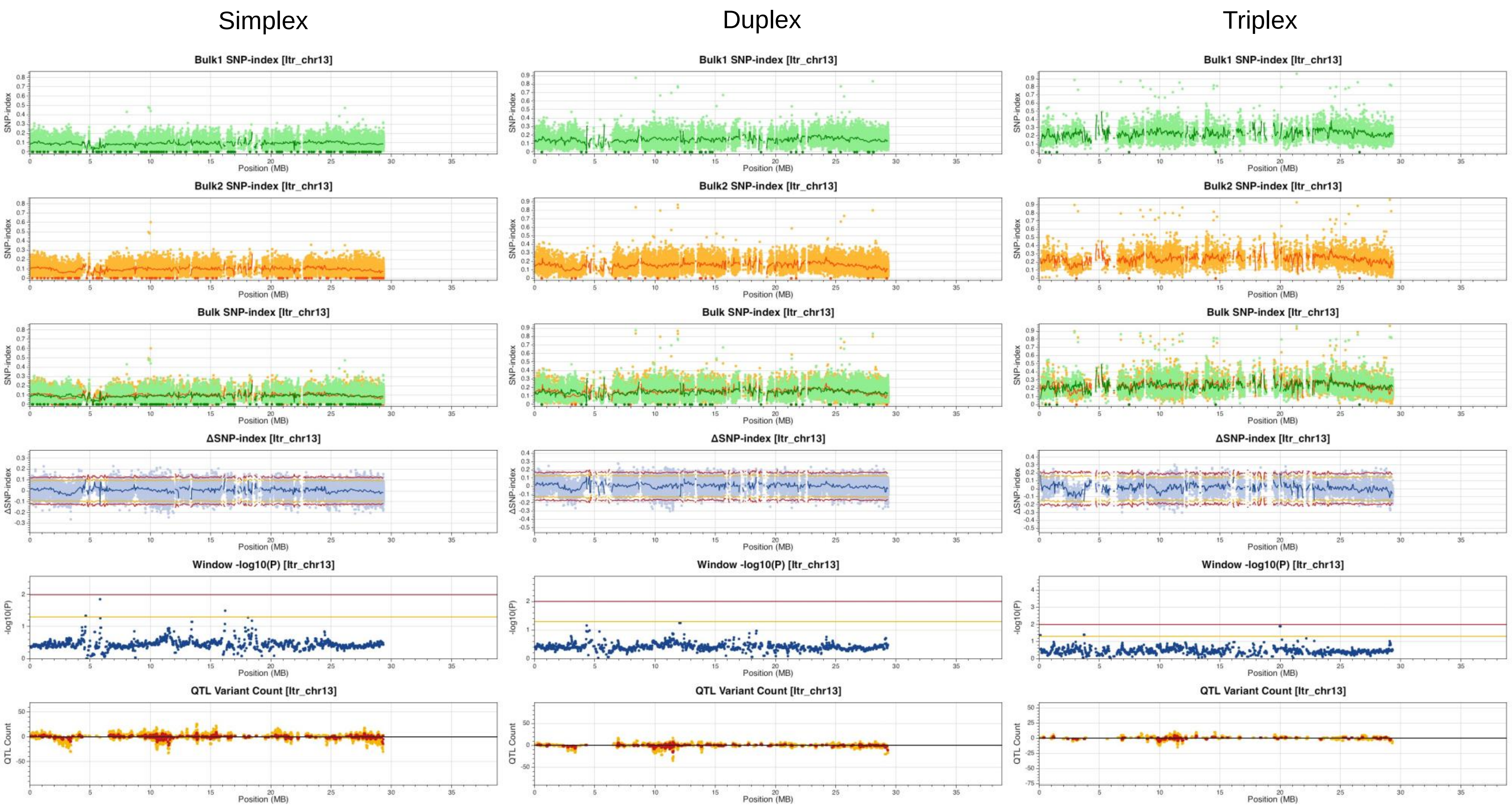

Supplemental Fig. 2. (continued)

A AH-derived variants

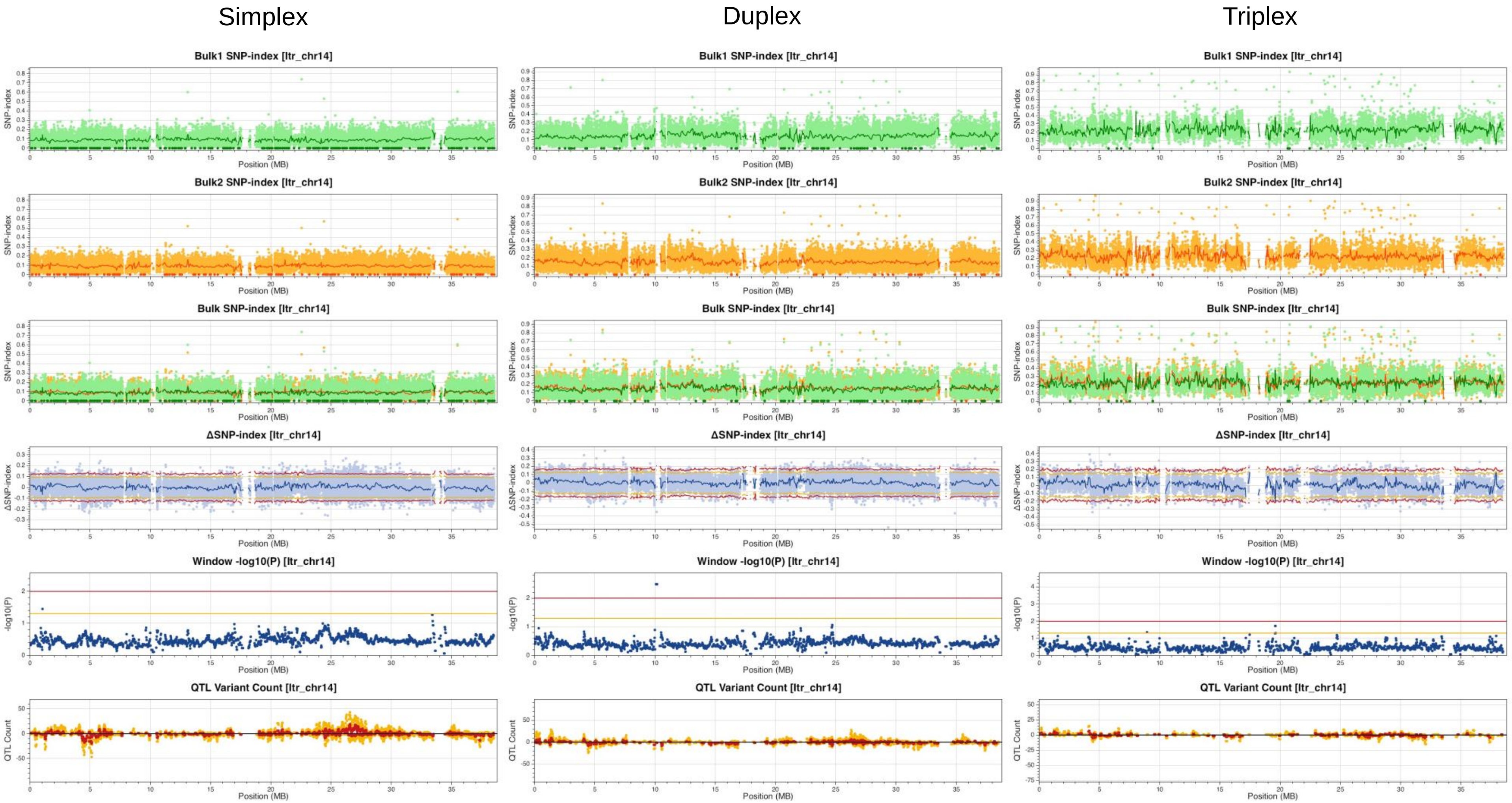

Supplemental Fig. 2. (continued)

A AH-derived variants

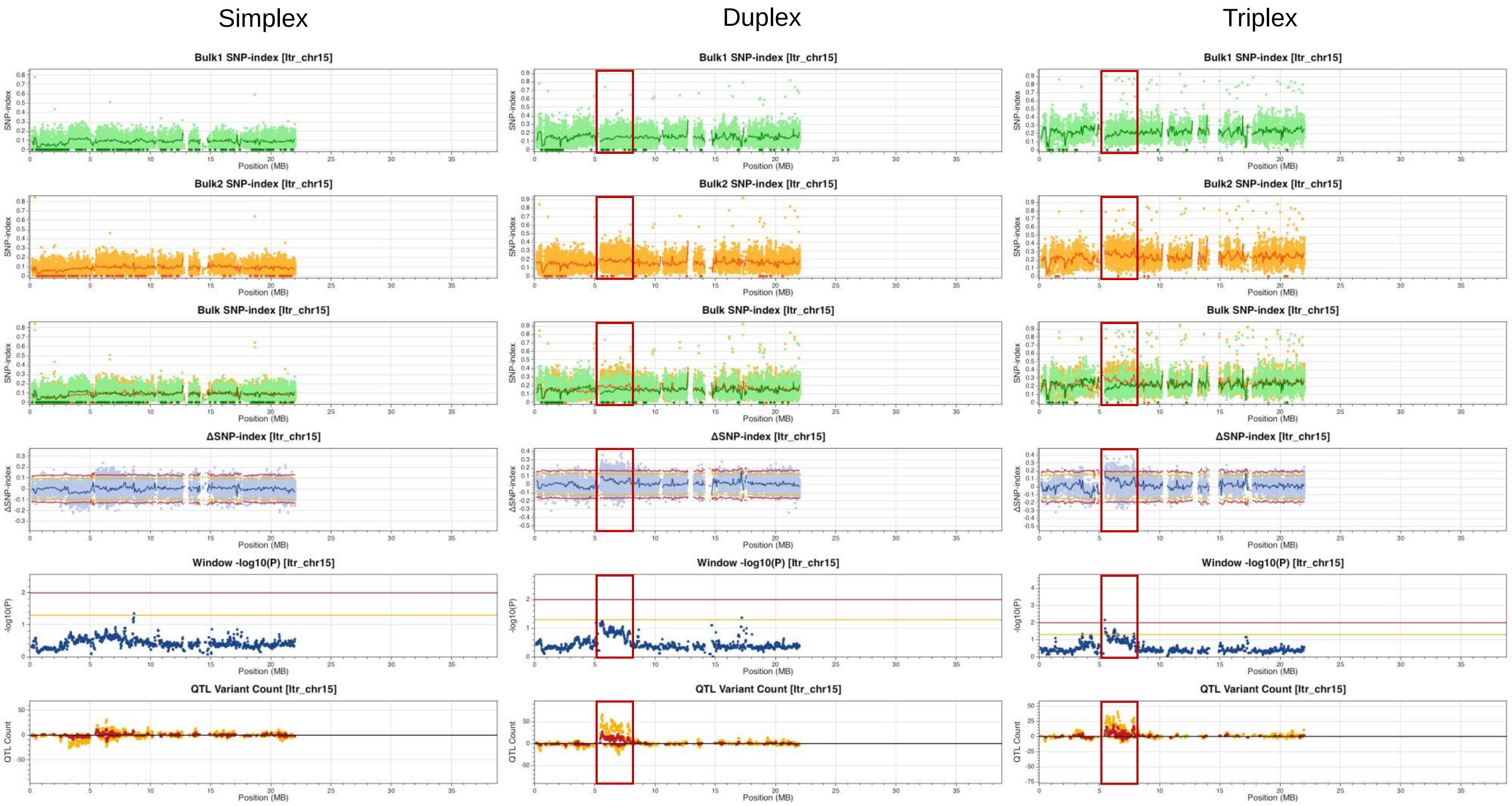

Supplemental Fig. 2. (continued)

B BK-derived variants

Simplex | Duplex | Triplex

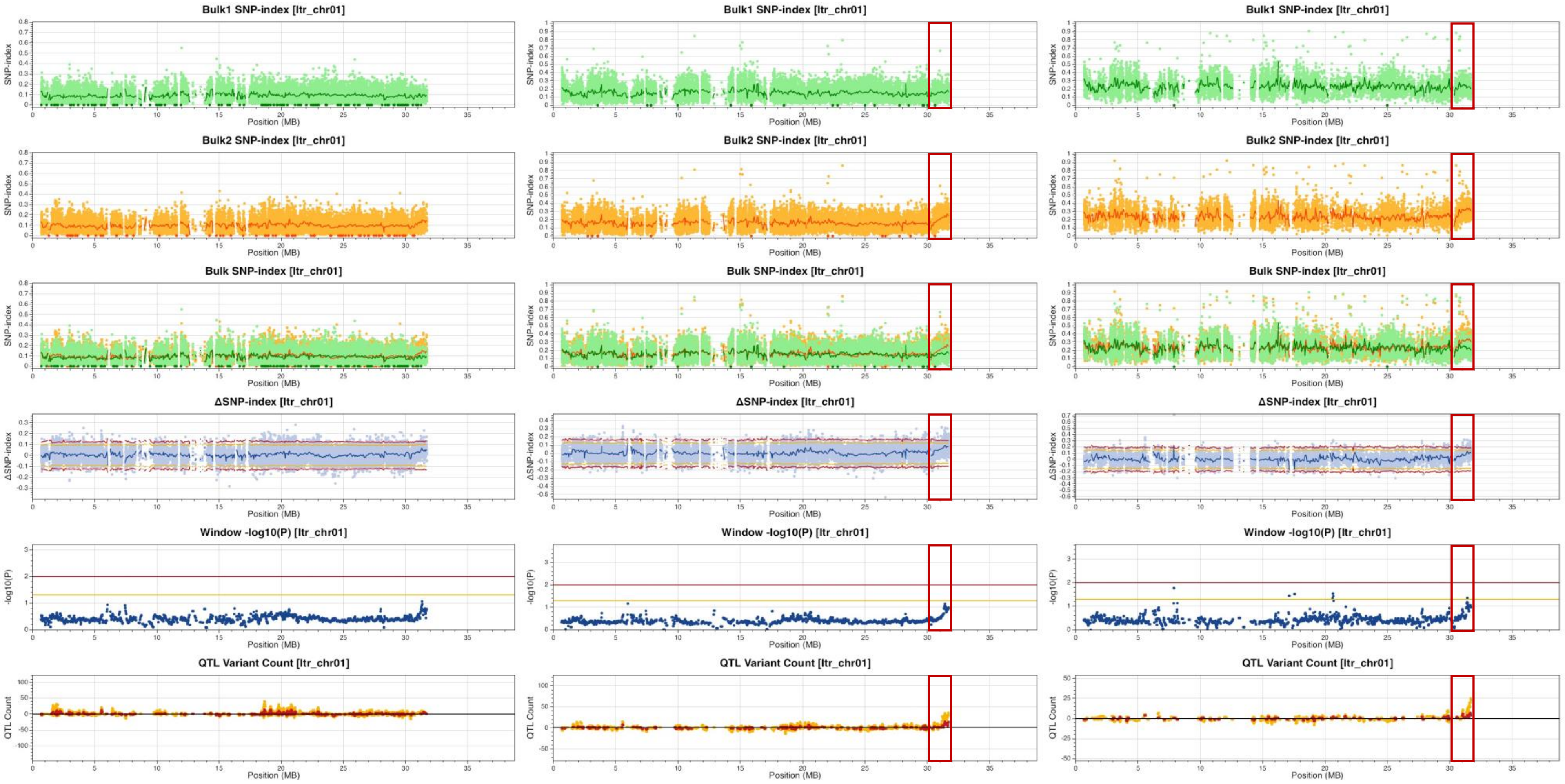

Supplemental Fig. 2. (continued)

B BK-derived variants

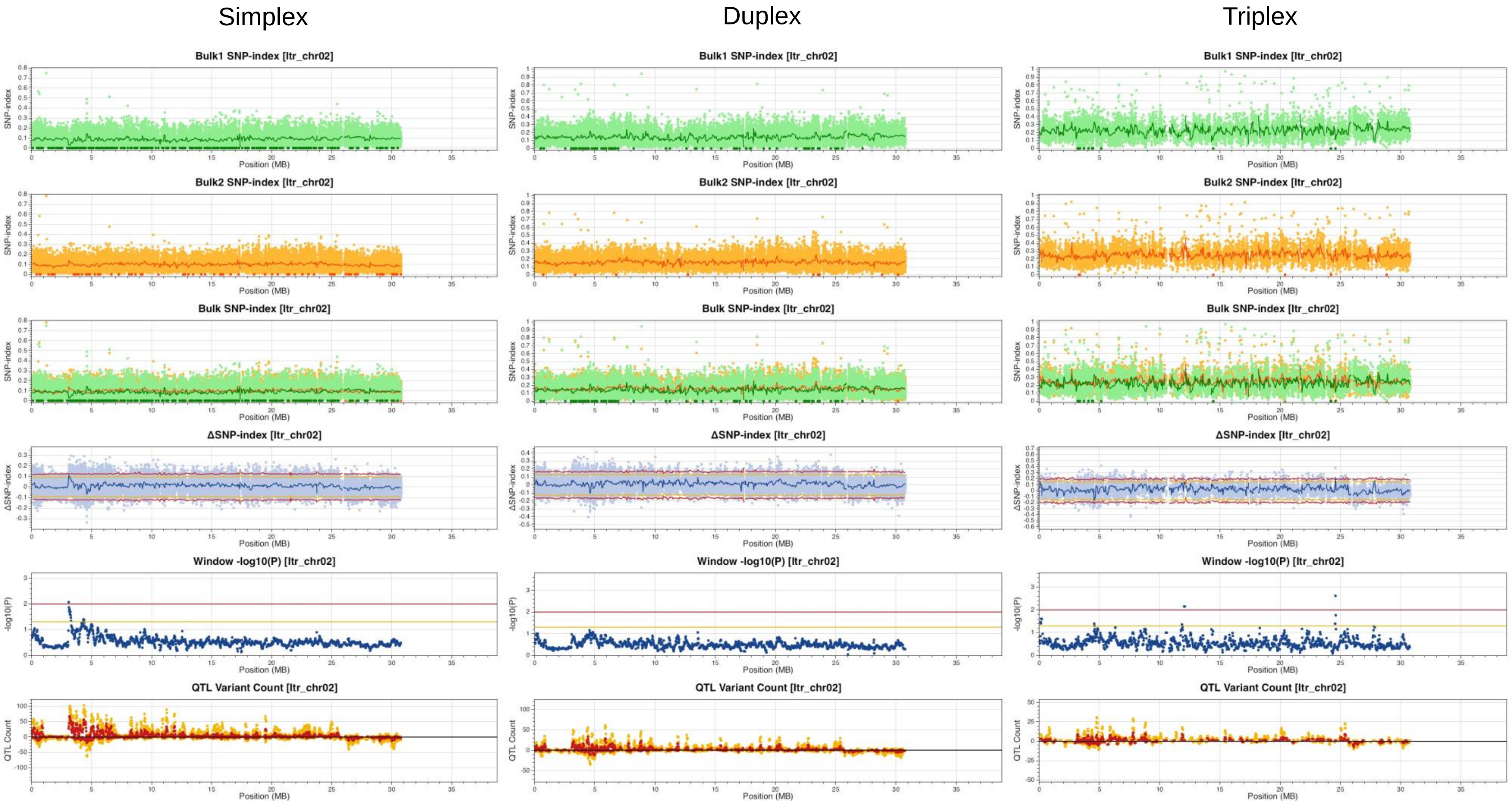

Supplemental Fig. 2. (continued)

B BK-derived variants

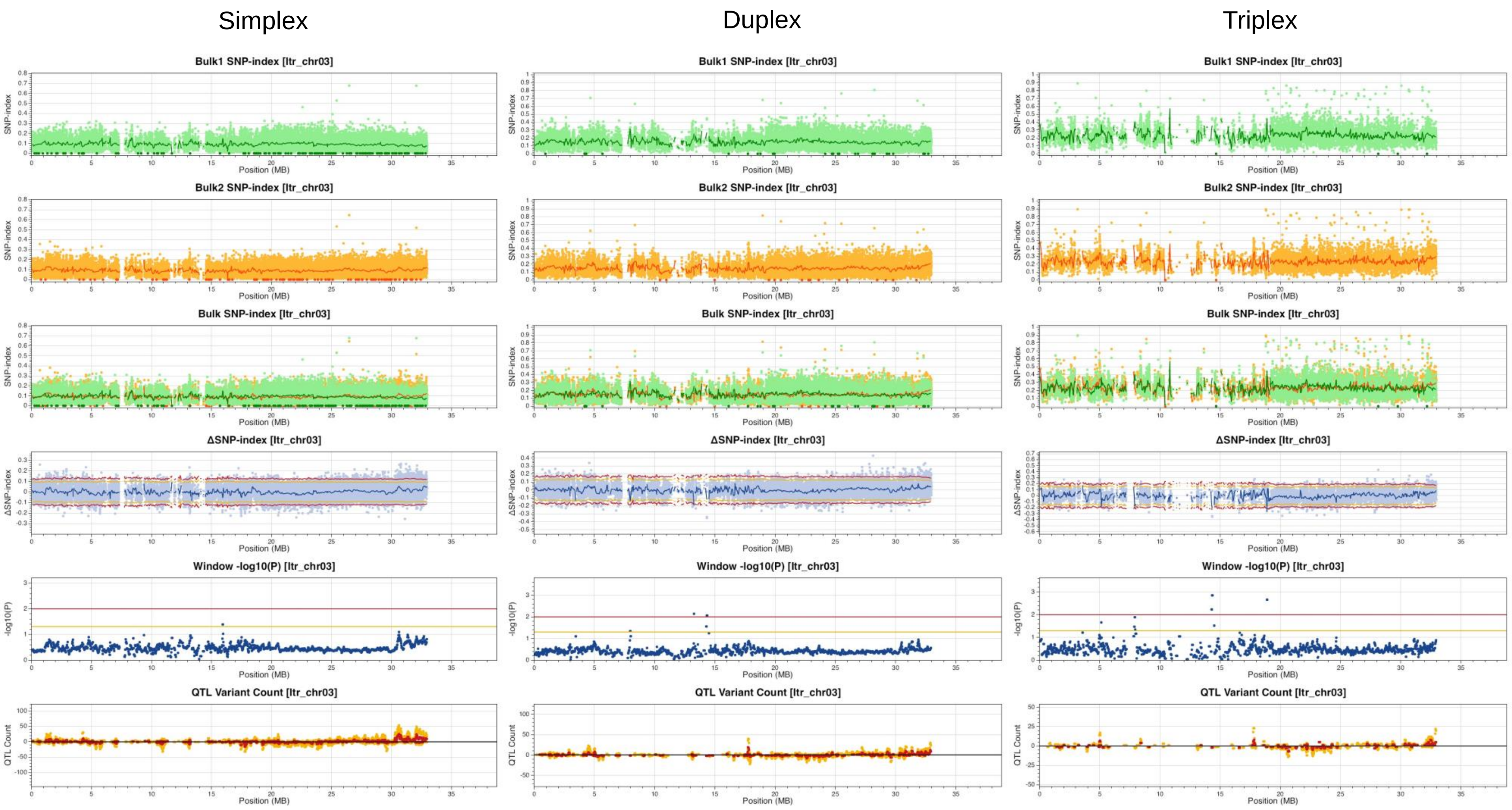

Supplemental Fig. 2. (continued)

B BK-derived variants

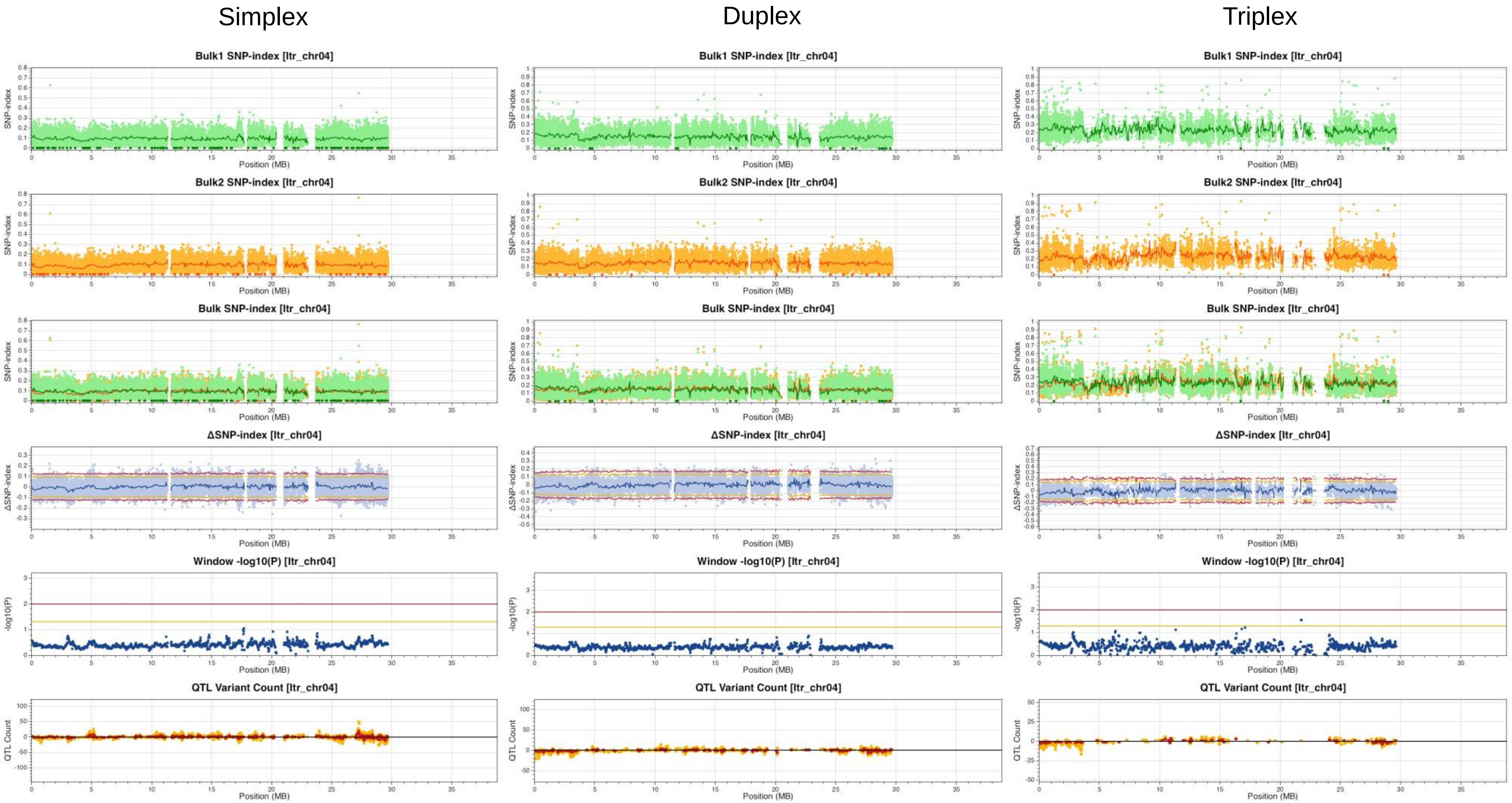

Supplemental Fig. 2. (continued)

B BK-derived variants

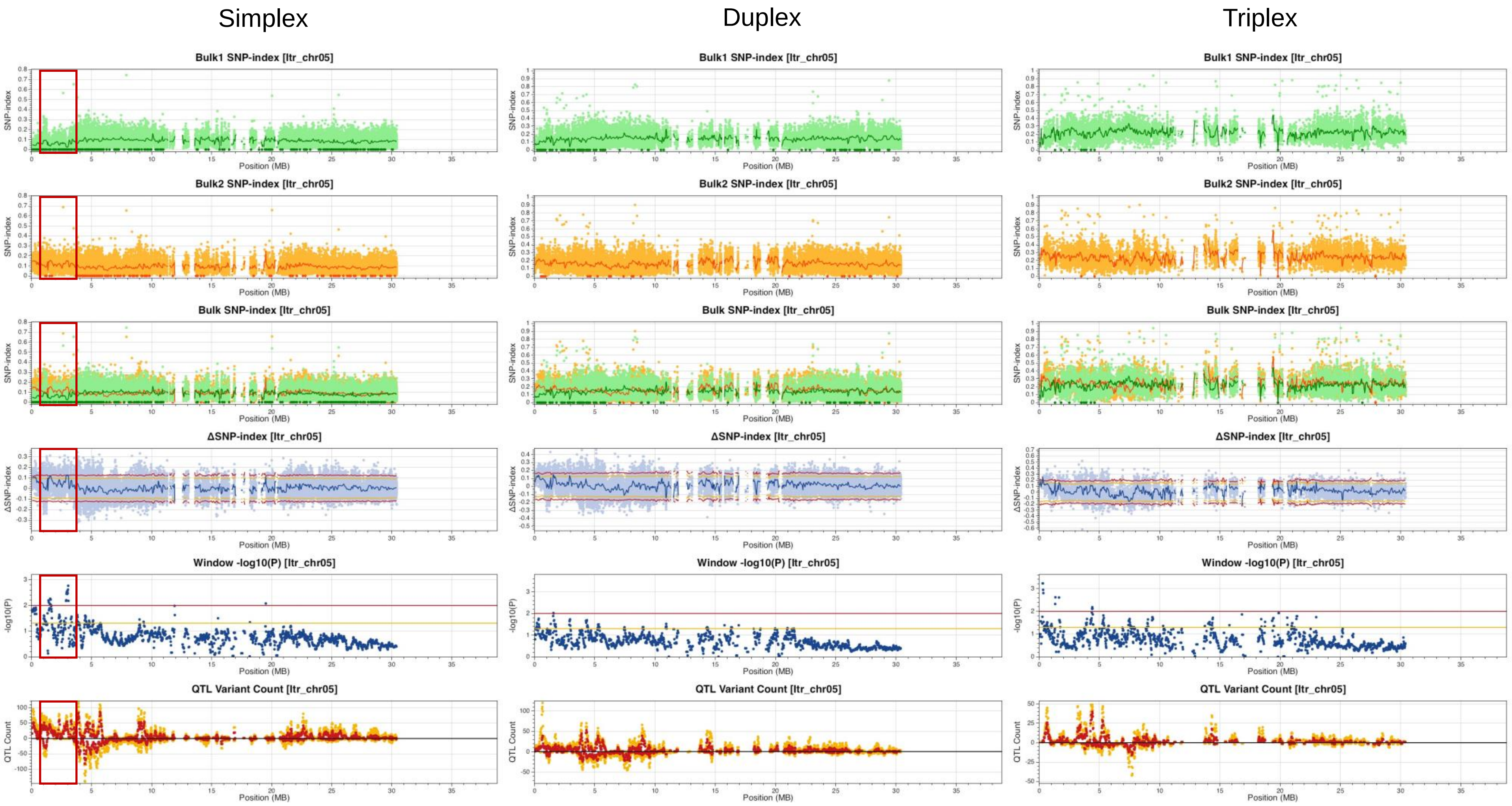

Supplemental Fig. 2. (continued)

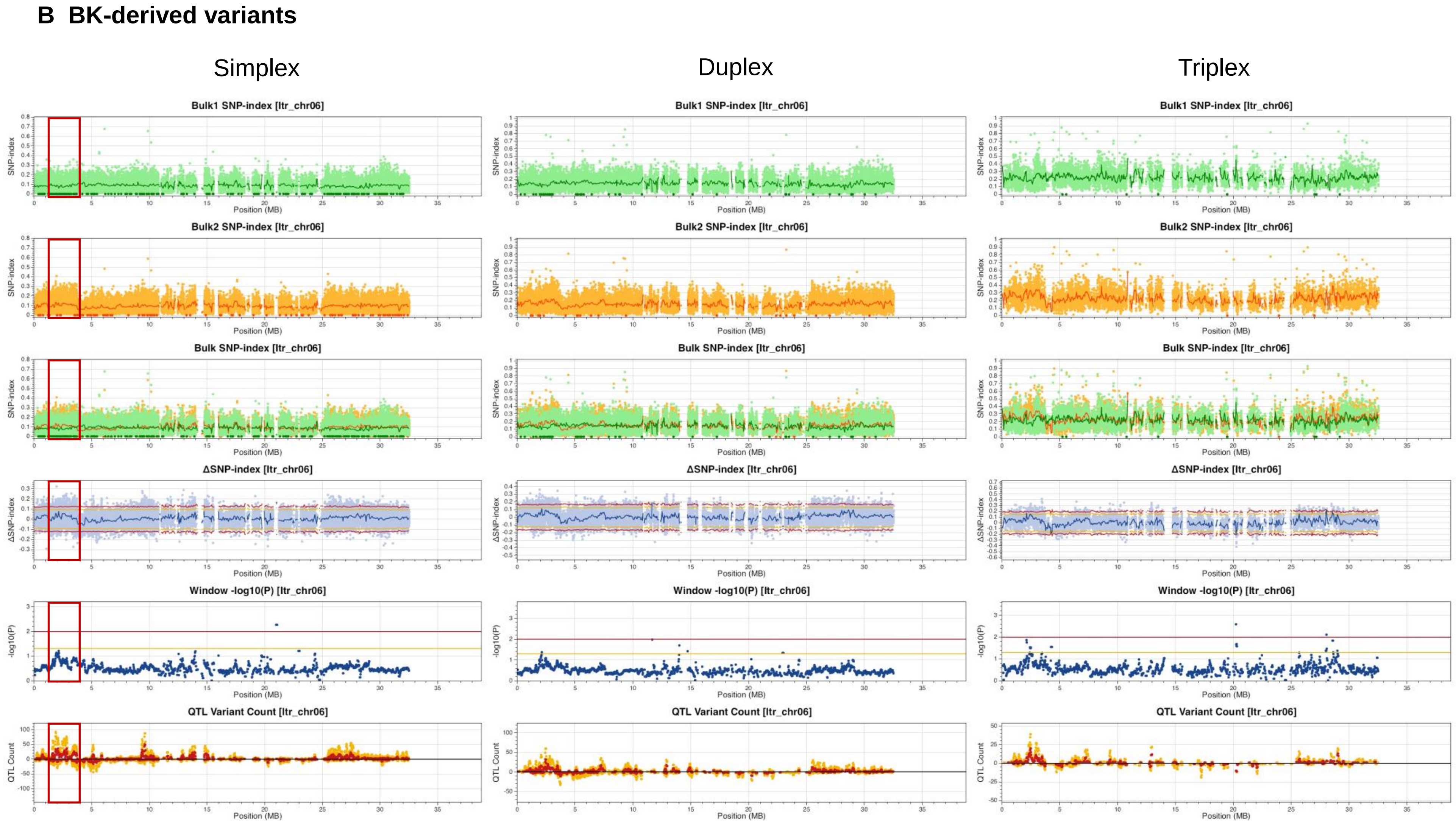

Supplemental Fig. 2. (continued)

B BK-derived variants

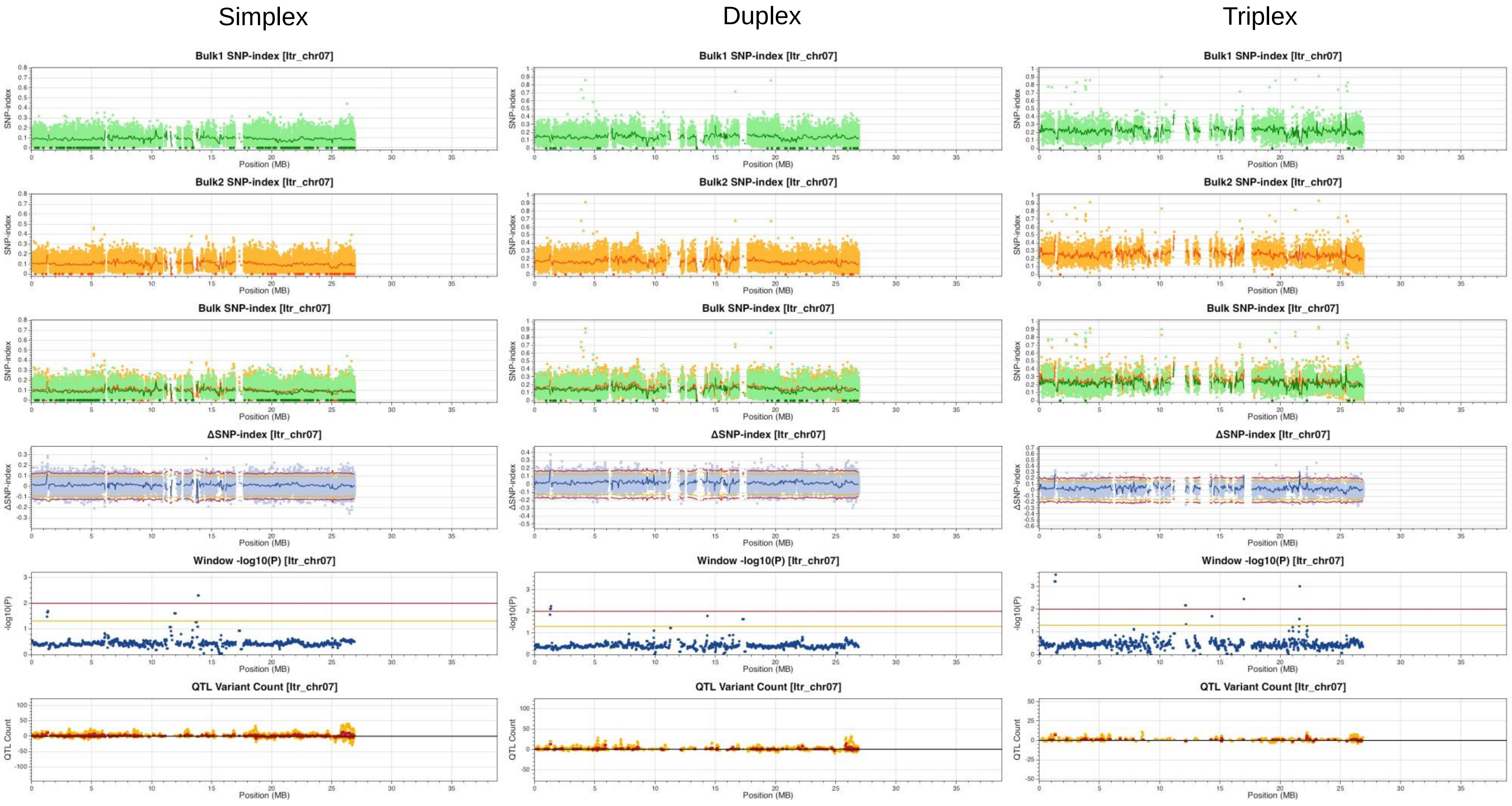

Supplemental Fig. 2. (continued)

B BK-derived variants

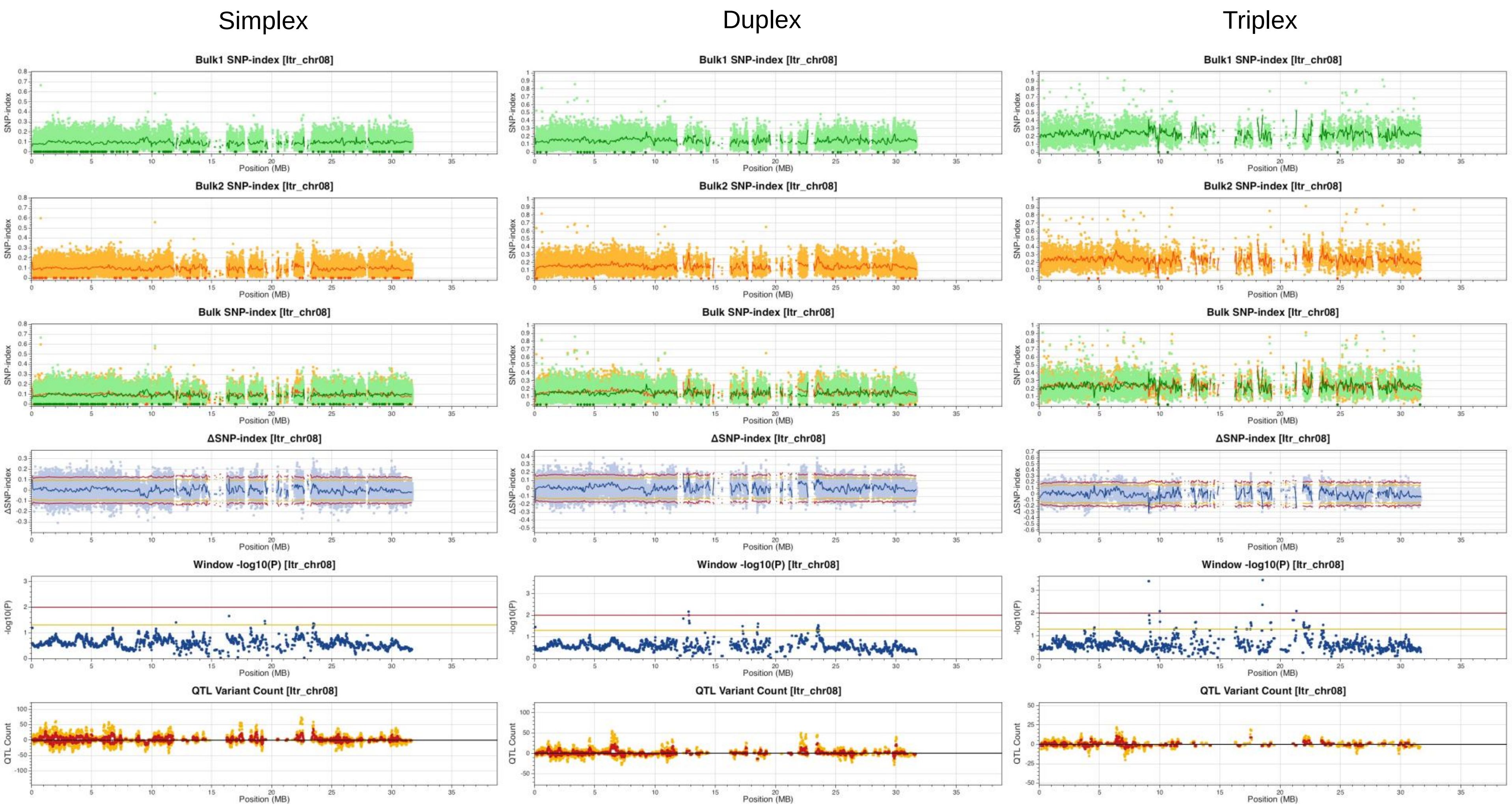

Supplemental Fig. 2. (continued)

B BK-derived variants

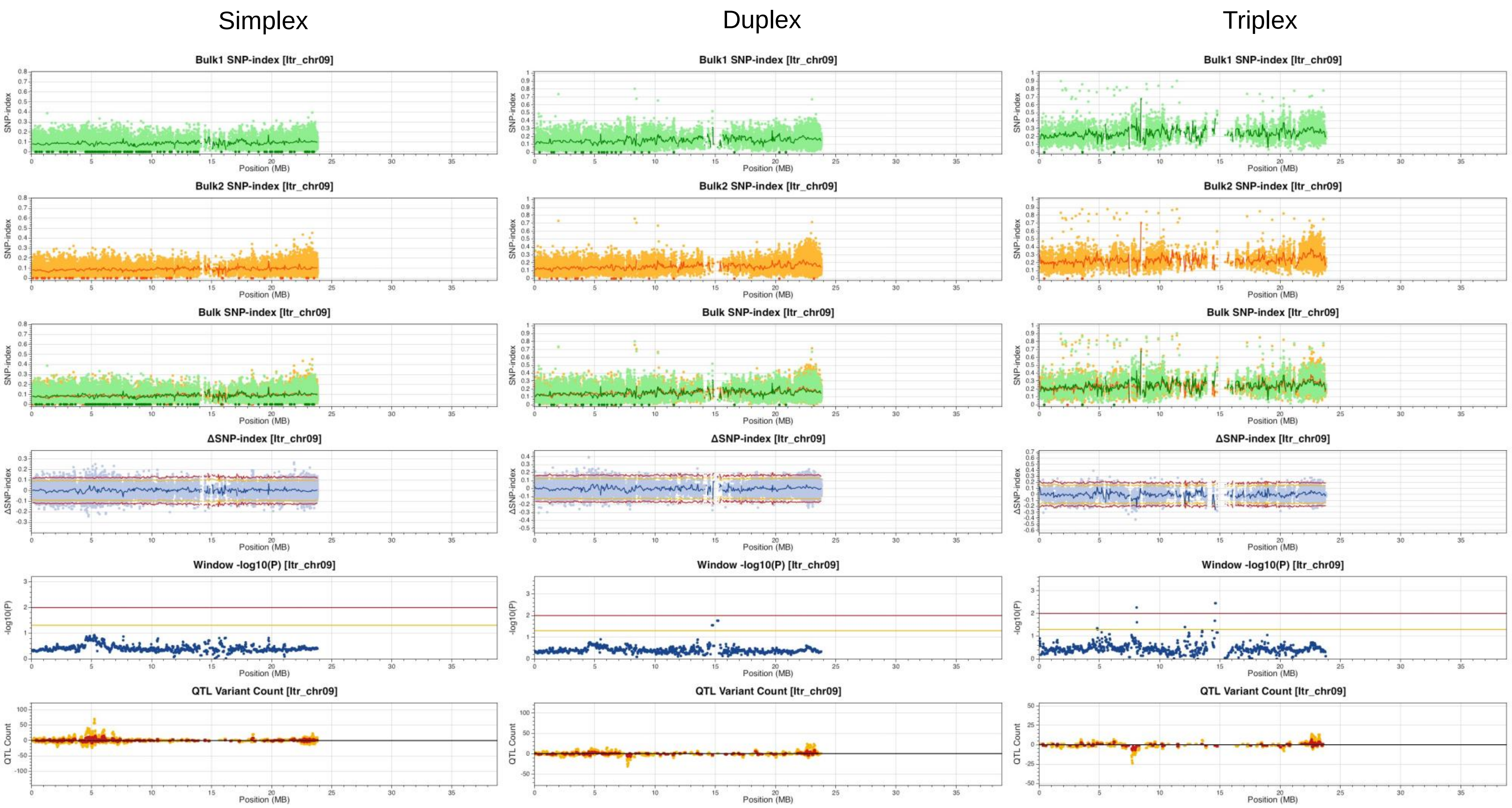

Supplemental Fig. 2. (continued)

B BK-derived variants

Simplex | Duplex | Triplex

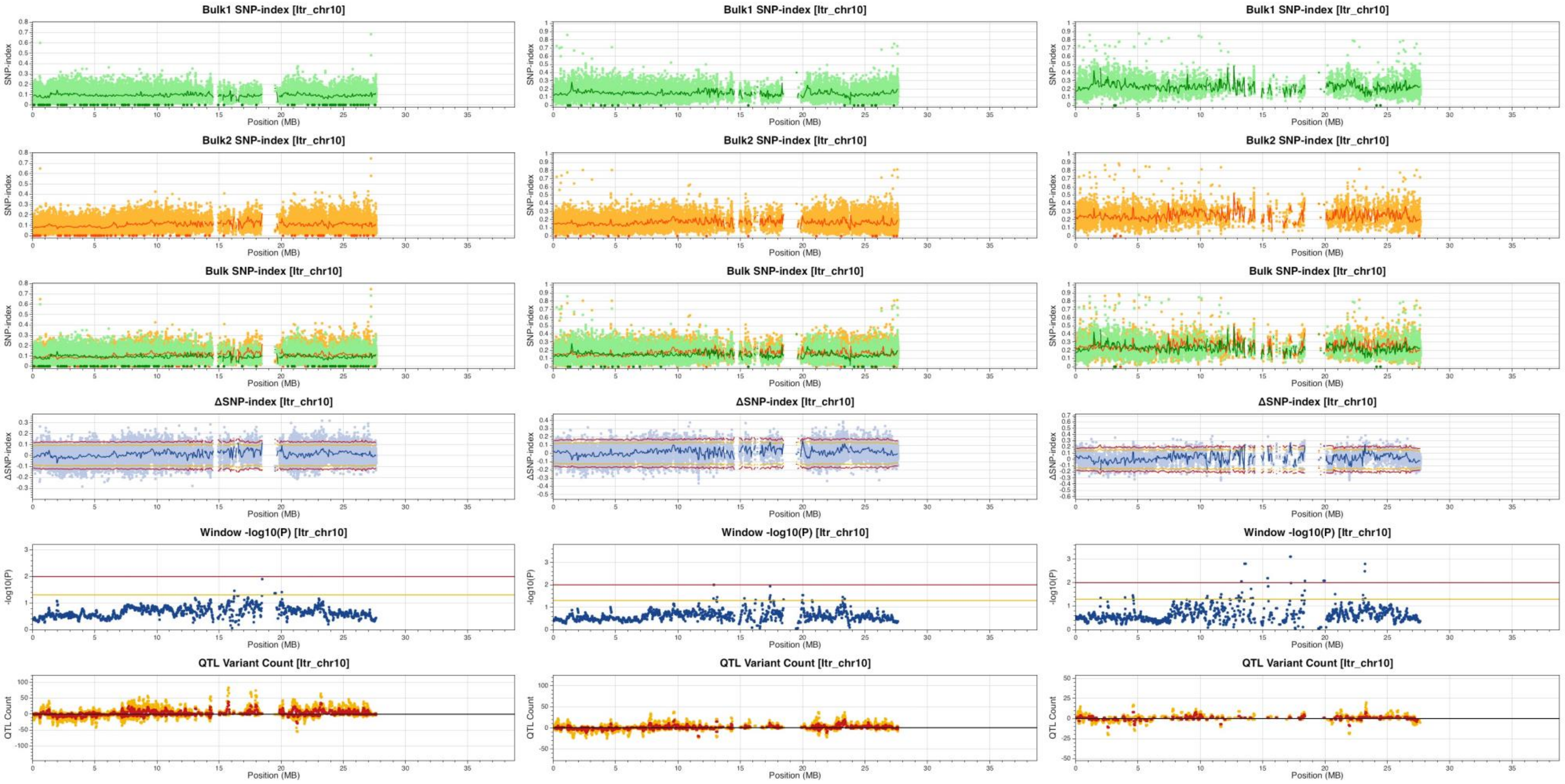

Supplemental Fig. 2. (continued)

B BK-derived variants

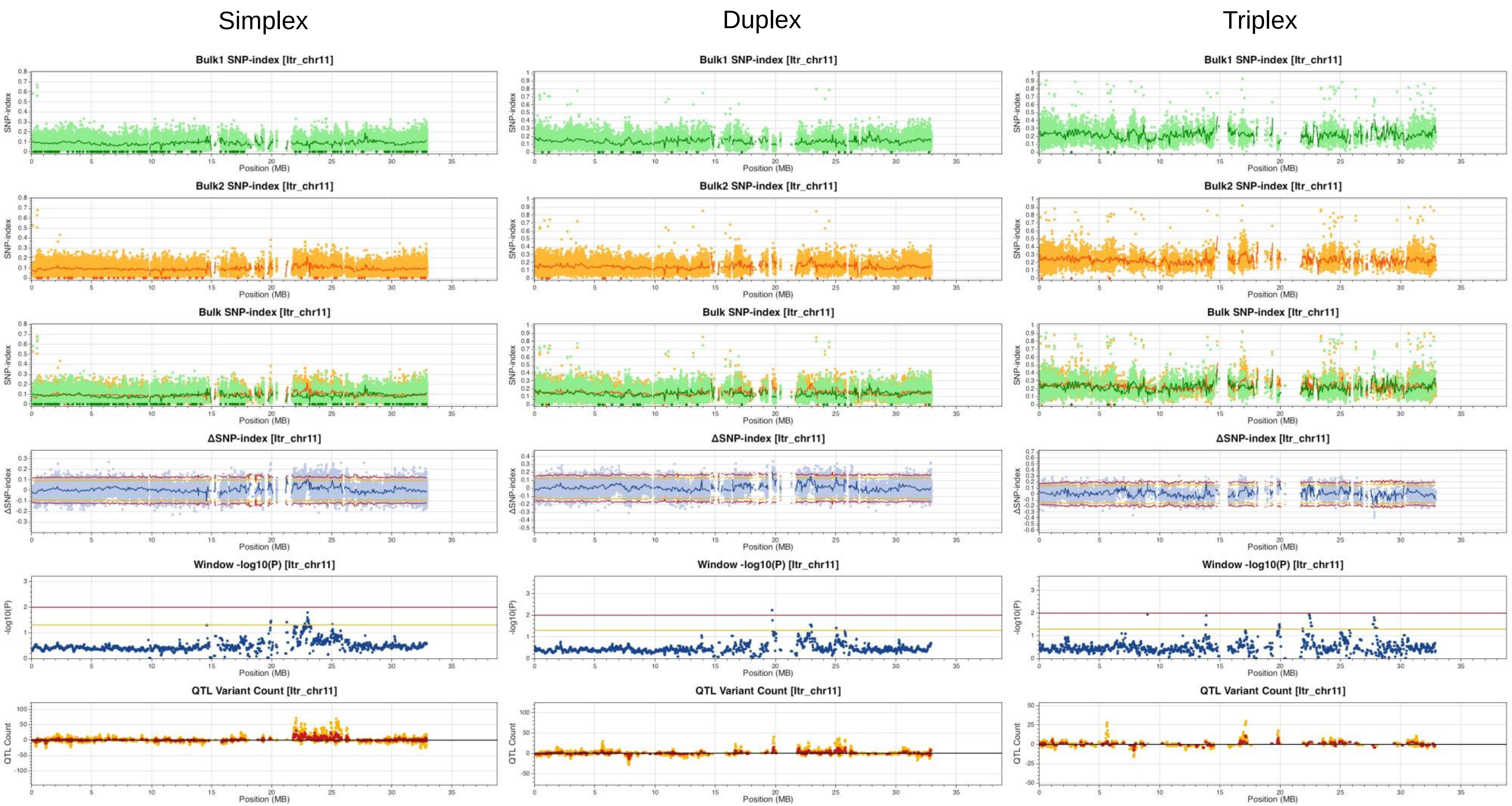

Supplemental Fig. 2. (continued)

B BK-derived variants

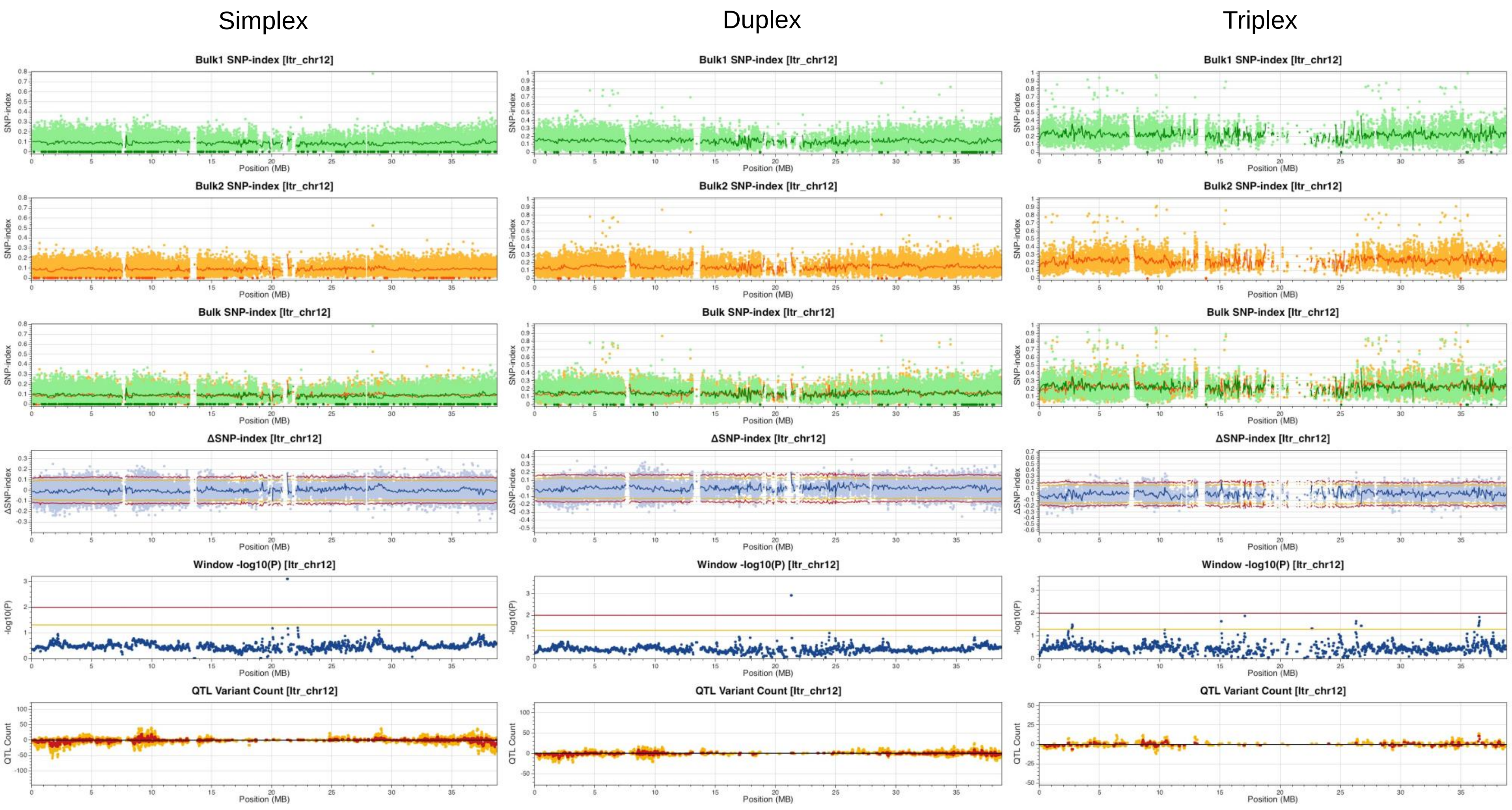

Supplemental Fig. 2. (continued)

B BK-derived variants

Simplex | Duplex | Triplex

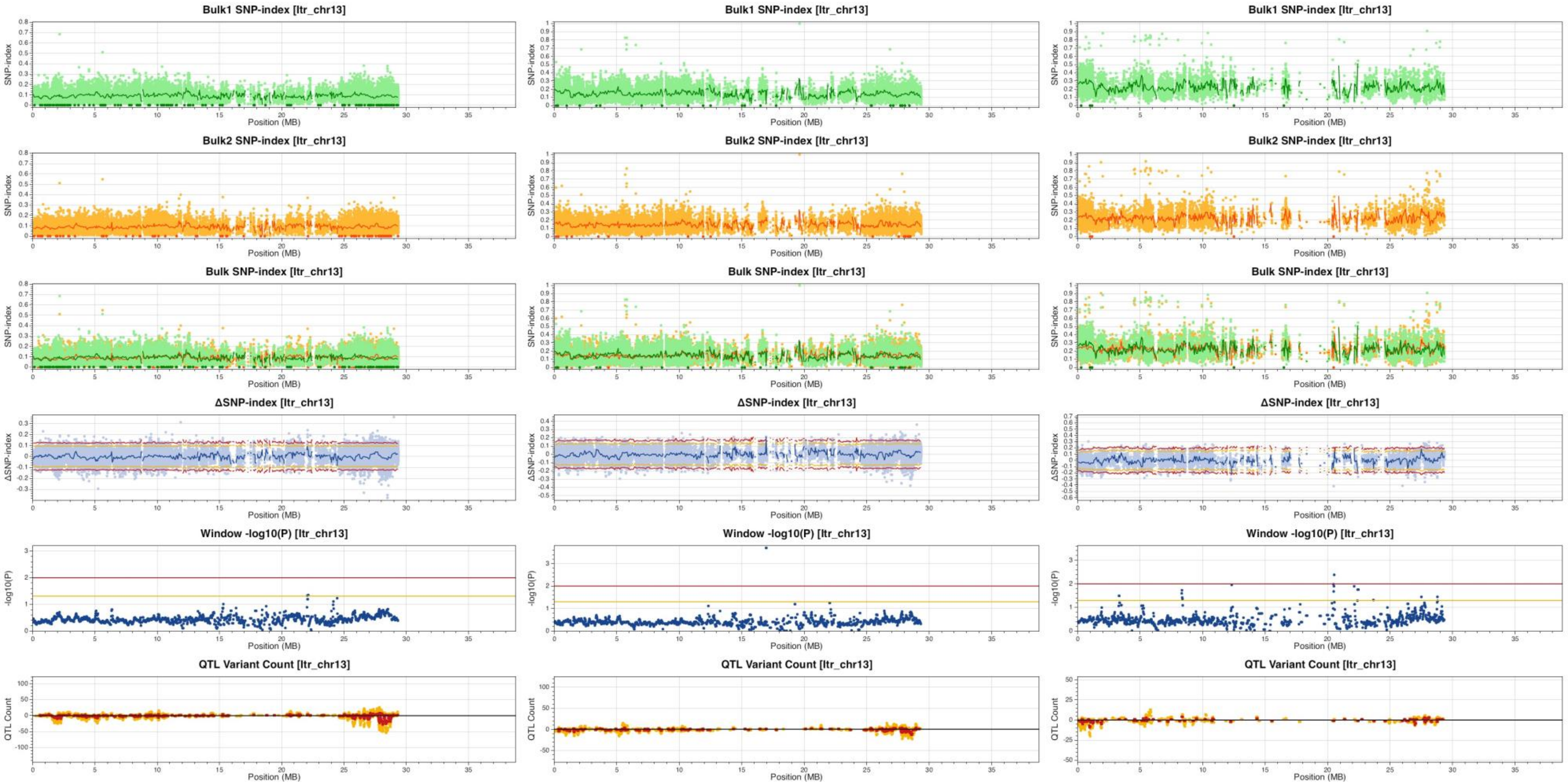

Supplemental Fig. 2. (continued)

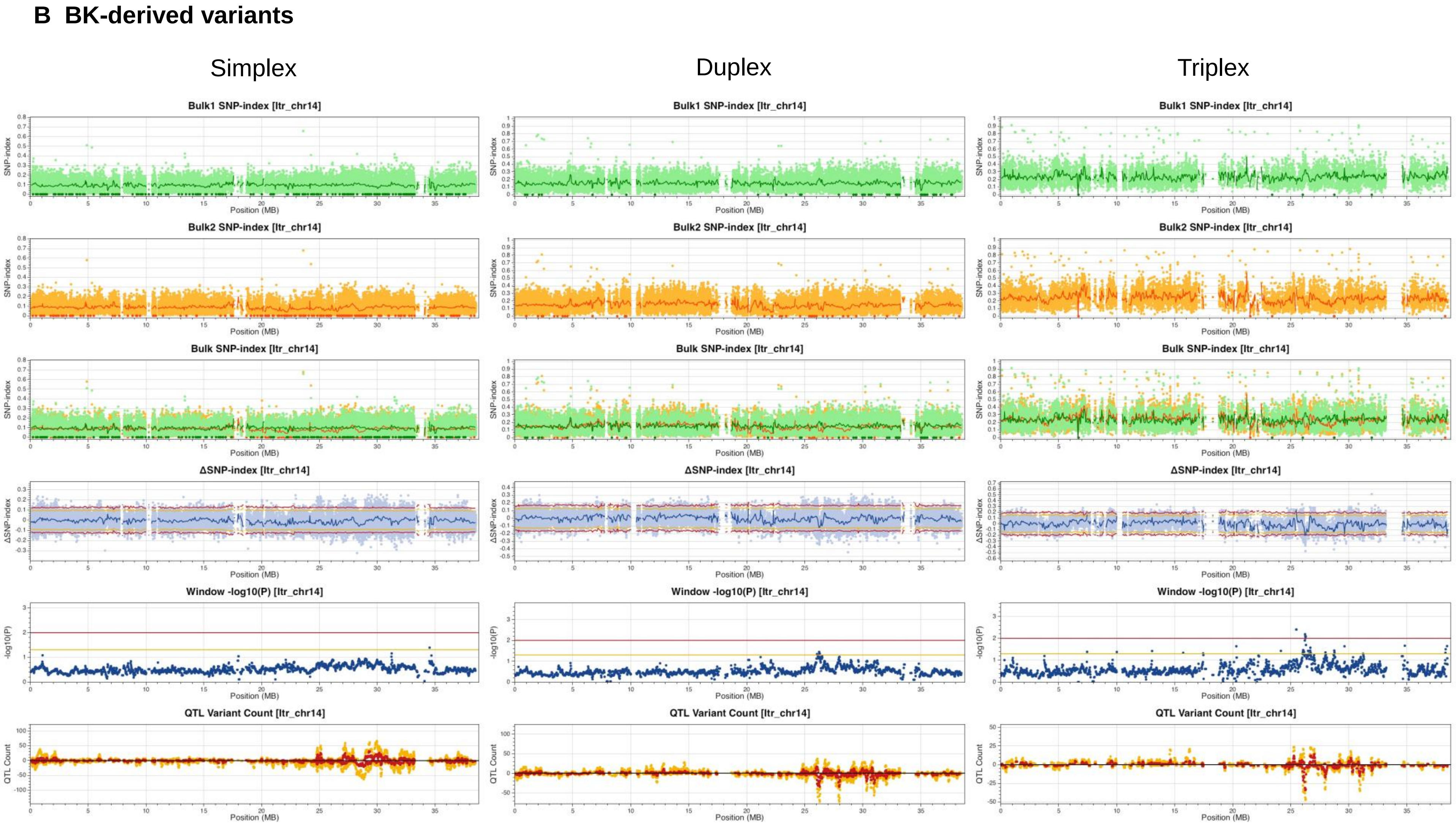

Supplemental Fig. 2. (continued)

B BK-derived variants

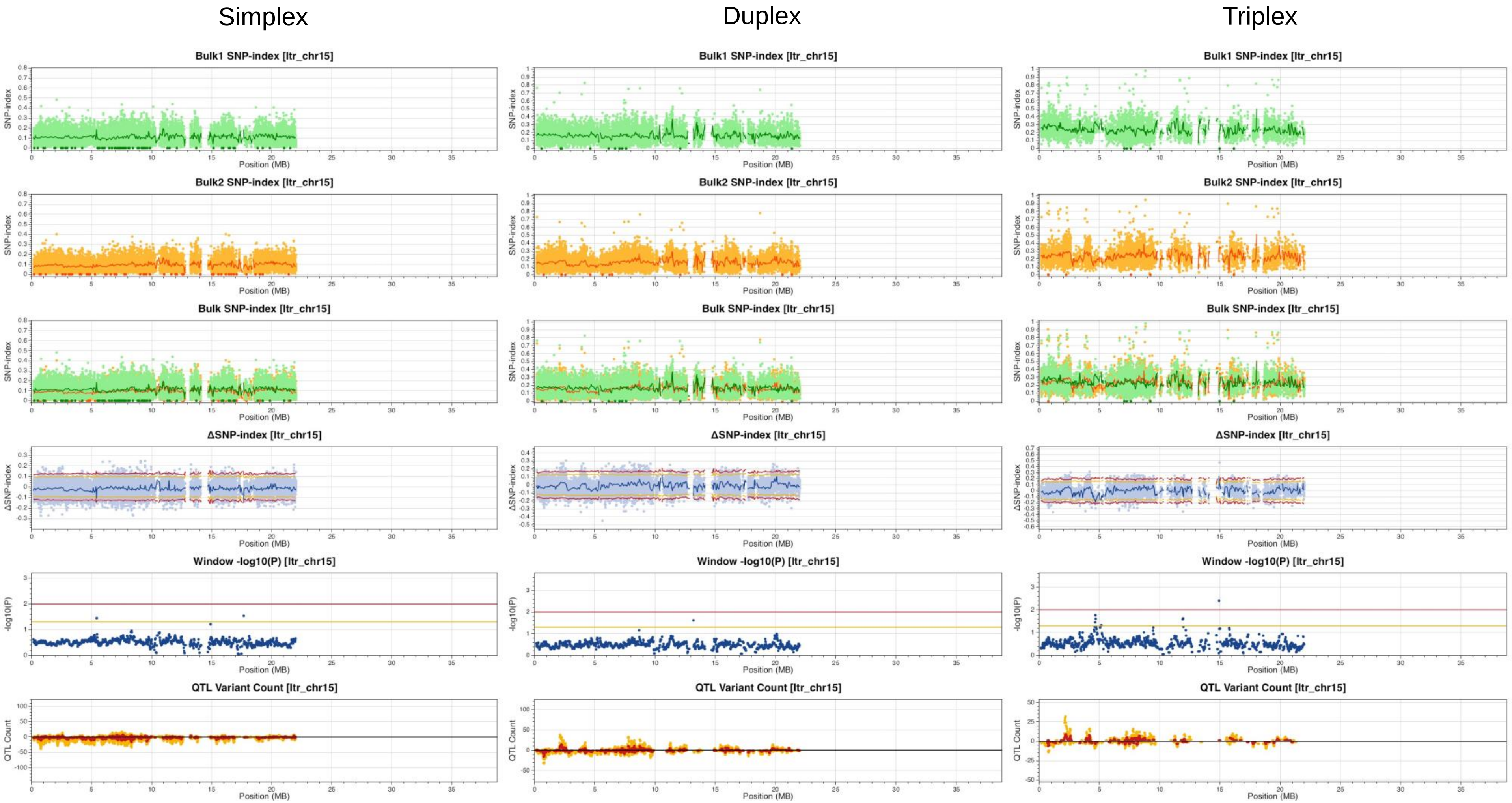

Supplemental Fig. 2. (continued)

A Wet Area Ratio

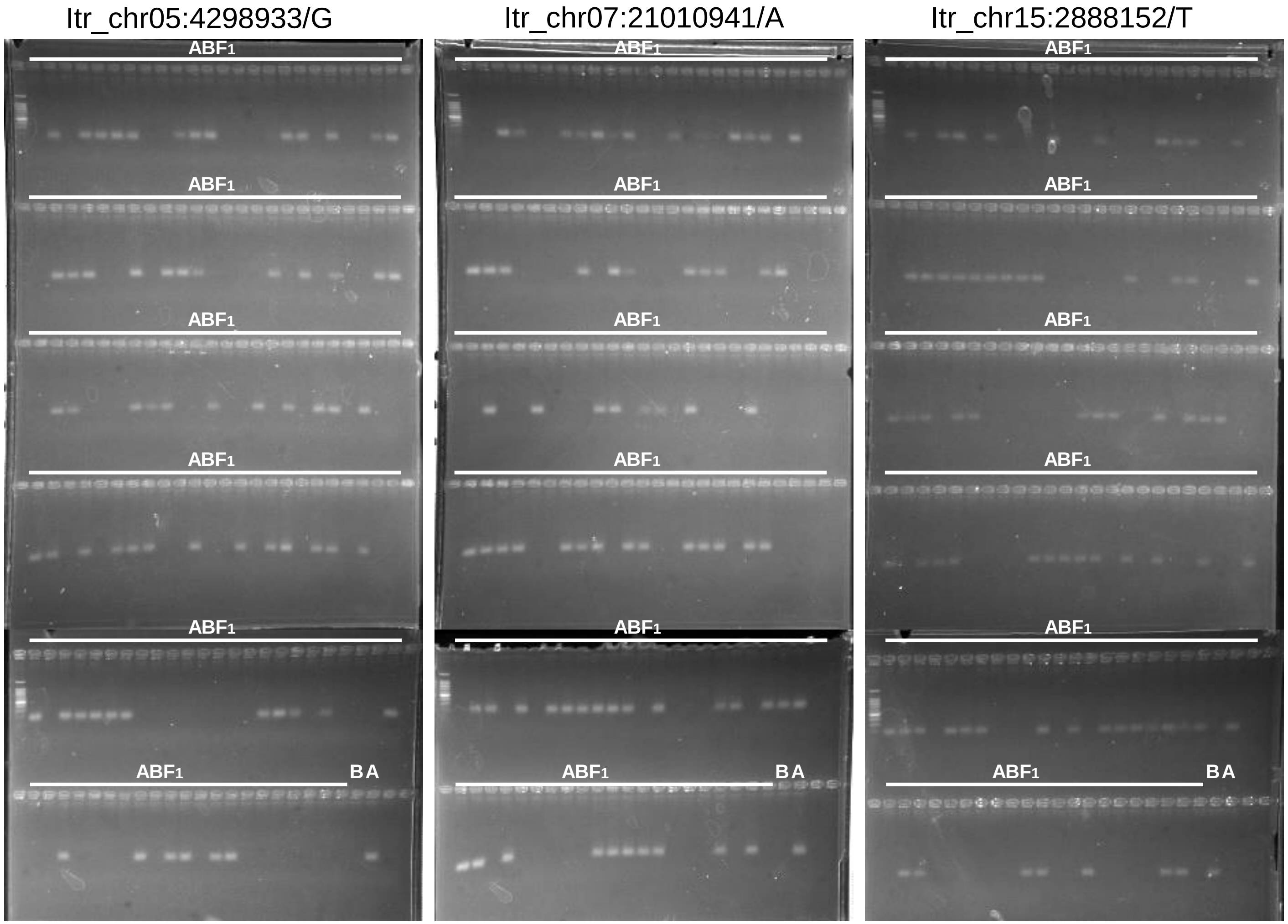

Supplemental Fig. 3. Agarose gel images of the DNA markers for genotyping the ABF1 progenies. (A) Markers for the QTL candidates of WAR. (B) Markers for the QTL candidates of GT. (C) *SSII* control. Asterisks denote the lines without amplification by the control primer set, that were eliminated from the genotyping analyses. ABF1: ABF1 progenies, A: AH, B: BK.

B Starch Gelatinization Temperature

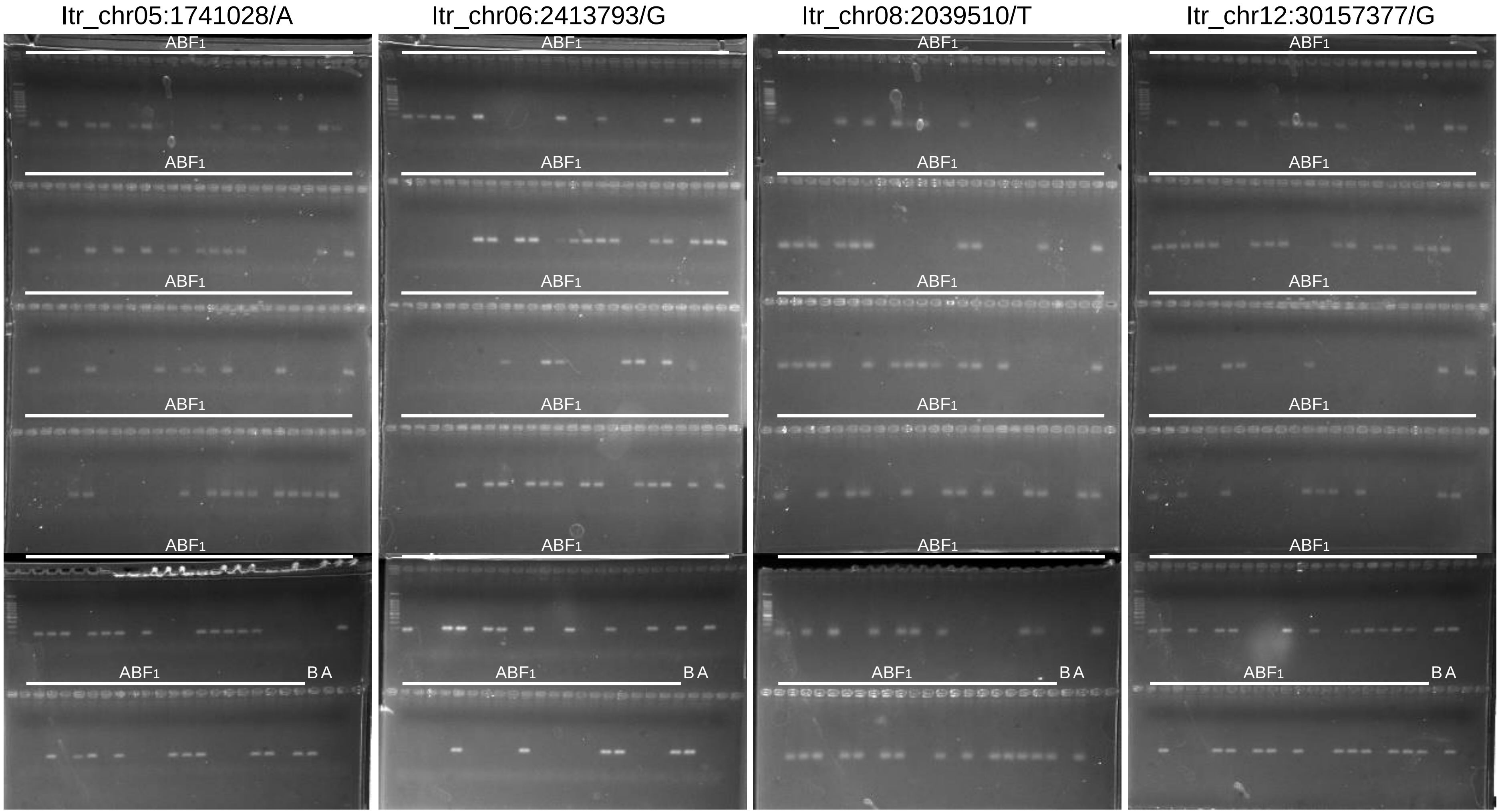

Supplemental Fig. 3. (continued)

C *SSII* (Control)

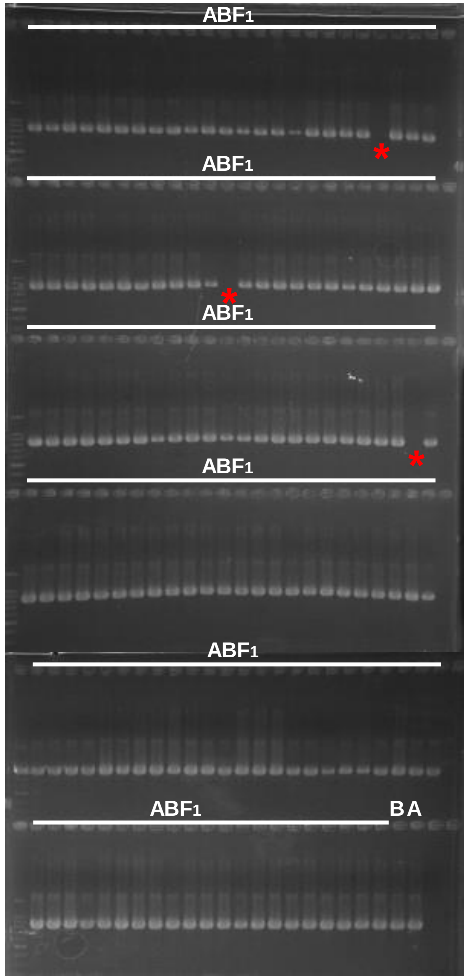

Supplemental Fig. 3. (continued)

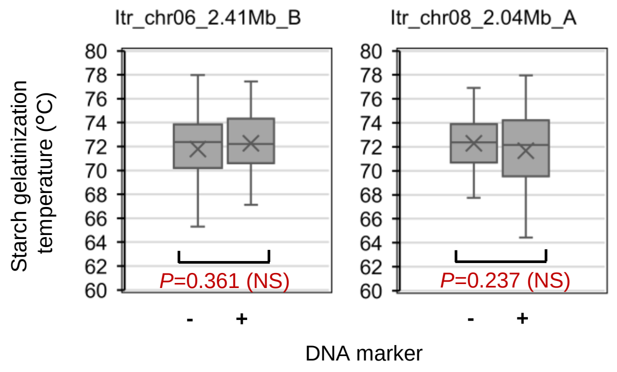

Supplemental Fig. 4. Genotyping using developed DNA markers. Evaluation of starch GT-linked SNP markers from BK (Itr_chr06_2.41Mb_B) and AH (Itr_chr08_2.04Mb_A) simplex variants. Relationships between marker genotypes (+; presence or −; absence) and starch GT are presented. The box plots are depicted similarly to Fig. 7. NS: not significant.

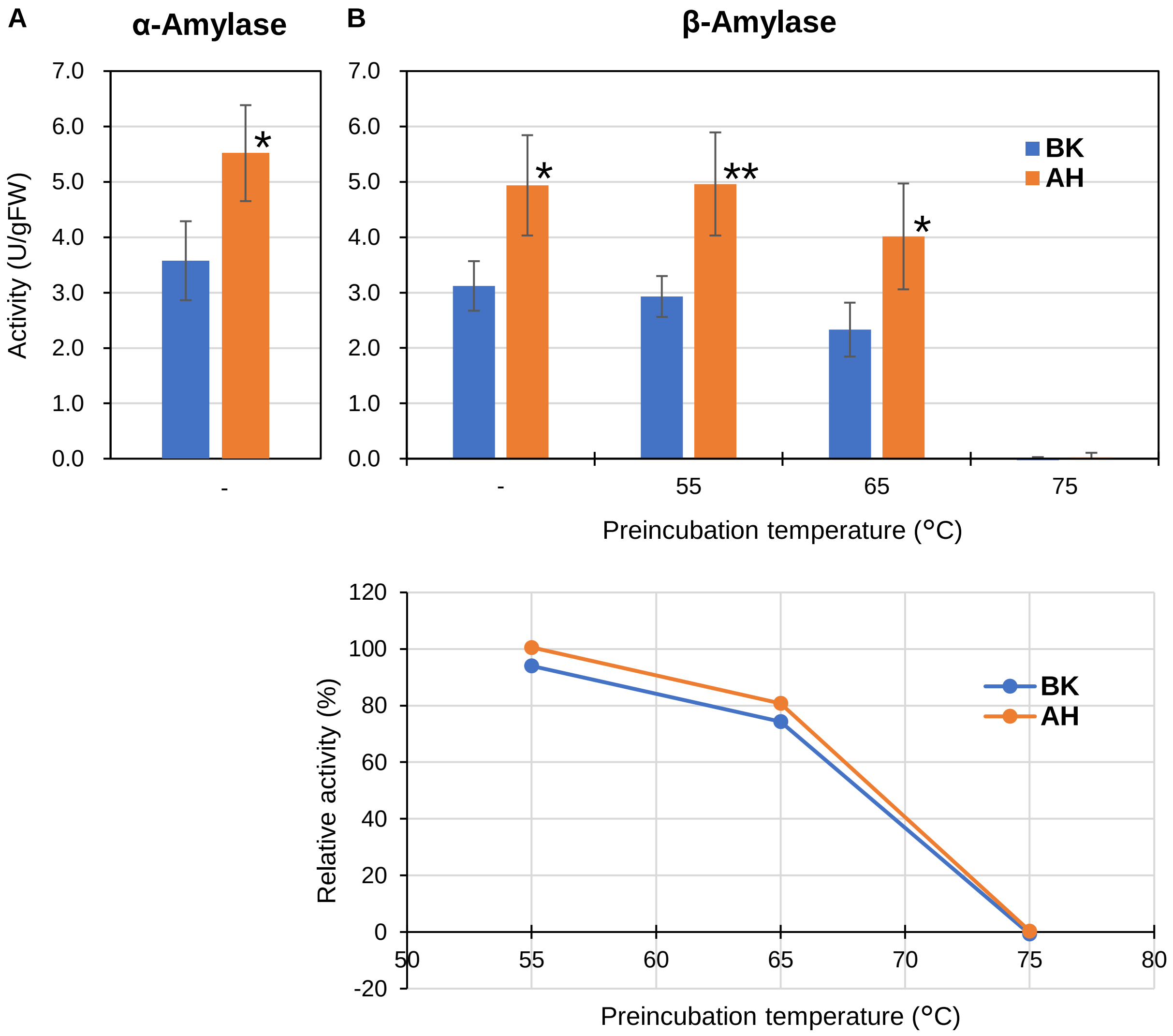

Supplemental Fig. 5. Starch-lytic enzyme activities in BK and AH tubers. (A) α-Amylase activity. (B) β-Amylase activity and thermostability. Activities before (-) and after incubation at the indicated temperatures were measured (upper panel). The relative activities were shown by setting the activities of the respective cultivars prior to the heat treatment (3.12 and 4.94 U/g flesh weight, respectively) to 100% (lower panel). Asterisks denote significant differences (Student's *t*-test, **P* < 0.05, ***P* < 0.01).

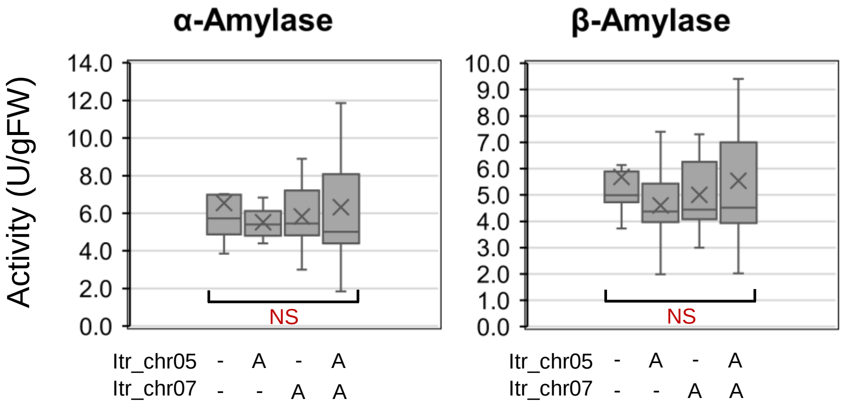

Supplemental Fig. 6. Effects of combined WAR-linked alleles on starch-lytic enzyme activities. Activities of α-amylase and β-amylase measured in tubers harboring different combinations of AH-derived markers. Ten lines each for harboring the indicated combination of presence (A) or absence (-) of Itr_chr05_4.30Mb_A and Itr_chr07_21.01Mb_A were measured for the activities of the starch-lytic enzyme. The box plots are depicted similarly to Fig. 7. Groups did not differ significantly (NS), as examined by ANOVA.
